# Prematurity and respiratory outcomes program (PROP): study protocol of a prospective multicenter study of respiratory outcomes of preterm infants in the United States

**DOI:** 10.1186/s12887-015-0346-3

**Published:** 2015-04-10

**Authors:** Gloria S Pryhuber, Nathalie L Maitre, Roberta A Ballard, Denise Cifelli, Stephanie D Davis, Jonas H Ellenberg, James M Greenberg, James Kemp, Thomas J Mariani, Howard Panitch, Clement Ren, Pamela Shaw, Lynn M Taussig, Aaron Hamvas, The Prematurity and Respiratory Outcomes Program Investigators

**Affiliations:** Department of Pediatrics, University of Rochester School of Medicine and Dentistry, Rochester, NY USA; Monroe Carrell Department of Pediatrics, Vanderbilt University School of Medicine, Nashville, TN USA; Department of Pediatrics, University of California, San Francisco School of Medicine, San Francisco, CA USA; Department of Biostatistics and Epidemiology, Perelman School of Medicine, University of Pennsylvania, Philadelphia, PA USA; Department of Pediatrics, Indiana University School of Medicine, Indianapolis, IN USA; Department of Pediatrics, Cincinnati Children’s Hospital Medical Center, Cincinnati, OH USA; Edward Mallinckrodt Department of Pediatrics, Washington University School of Medicine, St. Louis, MO USA; Department of Pediatrics and Pediatric Molecular and Personalized Medicine Program, University of Rochester School of Medicine and Dentistry, Rochester, NY USA; Department of Pediatrics, Perelman School of Medicine at the University of Pennsylvania, Philadelphia, PA USA; Department of Pediatrics, University of Colorado, Provost’s Office, University of Denver, Denver, CO USA; Department of Pediatrics, Ann & Robert H. Lurie Children’s Hospital, Northwestern University Feinberg School of Medicine, Chicago, IL USA

**Keywords:** Prematurity, Infant, Preterm, Chronic lung disease, Bronchopulmonary dysplasia

## Abstract

**Background:**

With improved survival rates, short- and long-term respiratory complications of premature birth are increasing, adding significantly to financial and health burdens in the United States. In response, in May 2010, the National Institutes of Health (NIH) and the National Heart, Lung, and Blood Institute (NHLBI) funded a 5-year $18.5 million research initiative to ultimately improve strategies for managing the respiratory complications of preterm and low birth weight infants. Using a collaborative, multi-disciplinary structure, the resulting Prematurity and Respiratory Outcomes Program (PROP) seeks to understand factors that correlate with future risk for respiratory morbidity.

**Methods/Design:**

The PROP is an observational prospective cohort study performed by a consortium of six clinical centers (incorporating tertiary neonatal intensive care units [NICU] at 13 sites) and a data-coordinating center working in collaboration with the NHLBI. Each clinical center contributes subjects to the study, enrolling infants with gestational ages 23 0/7 to 28 6/7 weeks with an anticipated target of 750 survivors at 36 weeks post-menstrual age. In addition, each center brings specific areas of scientific focus to the Program. The primary study hypothesis is that in survivors of extreme prematurity specific biologic, physiologic and clinical data predicts respiratory morbidity between discharge and 1 year corrected age. Analytic statistical methodology includes model-based and non-model-based analyses, descriptive analyses and generalized linear mixed models.

**Discussion:**

PROP incorporates aspects of NICU care to develop objective biomarkers and outcome measures of respiratory morbidity in the <29 week gestation population beyond just the NICU hospitalization, thereby leading to novel understanding of the nature and natural history of neonatal lung disease and of potential mechanistic and therapeutic targets in at-risk subjects.

**Trial registration:**

Clinical Trials.gov NCT01435187.

**Electronic supplementary material:**

The online version of this article (doi:10.1186/s12887-015-0346-3) contains supplementary material, which is available to authorized users.

## Background

Approximately 1 out of every 9 live births in the United States occurs prematurely. Preterm birth is associated with serious respiratory illnesses that are especially problematic in the first two years of life. Better understanding of the etiologies and risk factors for respiratory disease of prematurity is essential to effectively prevent and treat these disorders. The risks of developing respiratory disease in preterm infants are inversely related to their gestational age at birth (GA), with a diagnosis of bronchopulmonary dysplasia further increasing this risk. Yet, at any given GA, reliable clinical markers to quantify severity of future disease or predict which infants will develop long-term respiratory complications are lacking. Objective biochemical or physiologic measures for either clinical or research purposes are also rare. In recognition of the gaps in definitional, operational and mechanistic understanding, the Prematurity and Respiratory Outcomes Program (PROP) was created to characterize and develop a means of predicting clinically meaningful and persistent pulmonary disease of prematurity, in the context of current neonatal intensive care practices [1]. It is a multi-disciplinary, six-center, 13-site organization (Table 1) fostering the collaboration of neonatologists, pulmonologists, and basic scientists working to identify biomarkers of one-year respiratory morbidity and mortality in a cohort of more than 750 extremely preterm infants. The data gathered through this project will also be used to investigate mechanisms contributing to respiratory disease of the preterm newborns and to provide high-resolution phenotyping of disease severity for use in clinical applications and future trials.

**Table 1:** **Single center programs and specific projects**

**Program center**	**# Clinical sites**	**Project objectives**	**# Enrolled**
University of Pennsylvania	0	Data Coordinating Center for the PROP	N/A
Duke University/Indiana University	2	Gastrin-releasing peptide and bronchopulmonary dysplasia	105
University of Rochester/University at Buffalo	2	Functional and lymphocytic markers of respiratory morbidity in hyperoxic preterm infants	142
Cincinnati Children’s Hospital Medical Center	3	Biomarkers of immunologic function and preterm respiratory outcomes	111
University of California, San Francisco	3	Influence of the nitric oxide pathway and inflammation on preterm respiratory outcomes	161
Vanderbilt University	2	Immaturity and genetic variation in urea cycle-nitric oxide and glutathione pathways modulation of BPD phenotype	184
Washington University	1	Influence of the enteric microbiome on the genesis of bronchopulmonary dysplasia	132

This report of the study protocol details the design of the PROP study and illustrates the breadth of data and biospecimens that will be available at the end of the one-year follow-up period. It also suggests a multiplicity of future studies investigating complications and therapeutic interventions for the respiratory complications of prematurity.

## Methods/Design

The PROP required each Center to submit a single-site biomarker proposal along with a potential multi-site shared protocol for measuring respiratory phenotypes and outcomes of extremely preterm infants. The center-specific studies are reflected in the project objectives in Table 1, while the multicenter protocol was a collaborative effort.

**Figure 1:**
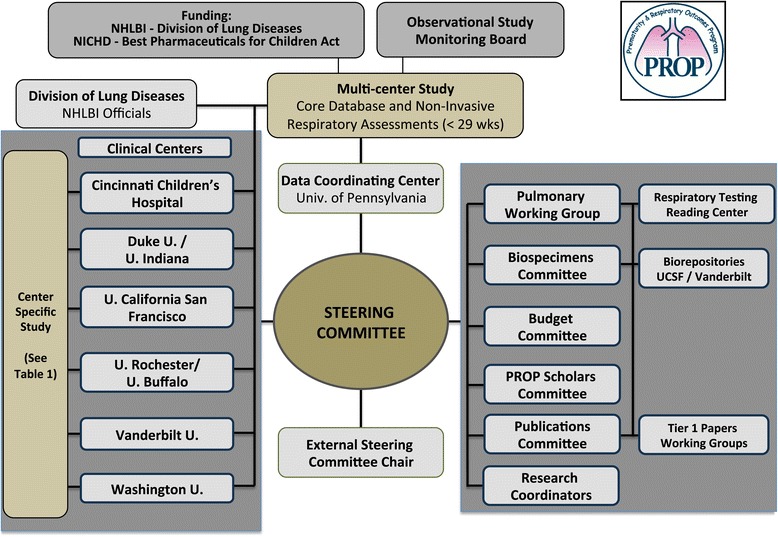
**Prematurity and Respiratory Outcomes Program (PROP) Structure and Logo.**

The PROP structure is depicted in Figure 1. In October 2011, an additional center composed of 2 clinical sites (Indiana University and Duke University) was added to the program based on the alignment of their research objectives. Each clinical center and the Data Coordinating Center (DCC) are represented on the Steering Committee, with each center contributing to the data collection, coordination and oversight of the multicenter components. The DCC manages clinical report forms, provides support for standardization of definitions, data collection, quality monitoring and analysis. Oversight is provided by an NHLBI appointed steering committee chair, NIH officials, and an observational and safety monitoring board (OSMB) with representatives from neonatology, pediatric pulmonology and biostatistics. The steering committee holds a conference call every 2 weeks, meets in-person twice yearly and in addition, holds working meetings at the American Thoracic Society and Pediatric Academic Society conferences. The committee identifies and resolves issues, encourages the centers to present updates of their projects, and determines future directions for the consortium. Working groups developed the initial protocols for biospecimen acquisition (Additional file 1), maternal and neonatal database elements (Additional files 2, 3, 4, 5 and 6) and respiratory measurements (Additional files 7 and 8: Table S1); these committees also provided regular monitoring of the standardization and quality of the measurements. The publications committee developed guidelines for authorship and a process for review and approval of manuscripts and abstracts prior to submission for publication or presentation.

Additional funding from the National Institute of Child Health and Human Development (NICHD) through the Best Pharmaceuticals for Children Act (BPCA) allowed expansion of the database collection to include comprehensive medication administration data (in hospital and post-discharge) and the creation of a “PROP Scholars” program to fund competitive and innovative PROP-related subprojects for trainees and junior faculty.

### Multicenter protocol development

#### Primary and secondary outcomes

A key scientific aim of PROP is to identify early clinical, physiologic, or biochemical biomarkers during the NICU hospitalization that can predict respiratory morbidity through 1 year of age. The primary outcome for PROP is the presence or absence of substantial post-prematurity respiratory disease, a composite obtained from longitudinal data in the first year post NICU discharge. Morbidity in four domains is examined: respiratory symptoms, medication use, hospitalizations and dependence on technology during the first year of life and results of infant pulmonary function testing at 1 year of age in a subset of participants. Mortality from cardiorespiratory cause is incorporated as well.

Secondary outcomes include death, near-term “BPD” status, the PROP Physical Exam Score and a Respiratory Morbidity Severity Score at one year, summarizing severity across the morbidity domains.

#### Protocol

**Figure 2:**
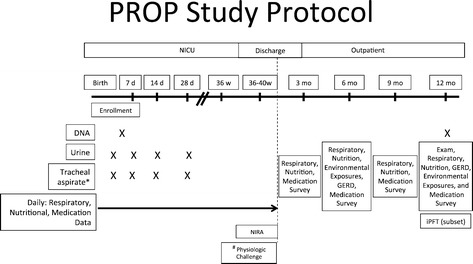
**PROP Study Protocol Time Line spans from birth to one year of corrected age collecting health data and biospecimens.** *Tracheal aspirate samples were collected if the infant was intubated and clinically required suctioning. ^#^Physiologic challenge testing was either from oxygen to room air (21% oxygen, the “Room Air Challenge”) or from room air to 15% oxygen (“hypoxia challenge”) depending on status. NIRA: Non-invasive respiratory assessment; GERD: gastroesophageal reflux disease; iPFT: infant pulmonary function testing.

The inclusion and exclusion criteria are listed in Table 2 and the protocol is outlined in Figure 2. The protocol is designed to provide the highest resolution phenotyping possible with the mandate to use standardized, widely applicable, non-invasive methods for data and biospecimen collections that will reflect a continuum of disease and care.

**Table 2:** **Inclusion/Exclusion criteria**

	**Inclusion criteria**
-	23 0/7 to 28 6/7 weeks using best obstetrical estimate
-	7 days of age or less at enrollment
	**Exclusion criteria**
-	Concern for viability
-	Structurally significant congenital heart disease
-	Structural abnormalities of the upper airway, lungs or chest wall
-	Congenital malformations or syndromes that adversely affect life expectancy or cardio-pulmonary development
-	Family unlikely to be available for long-term follow-up

The sample size for the multicenter study was pre-specified to include 750 surviving infants at a 36-week postmenstrual age (PMA) time-point. Power calculations consider a simplified binary outcome of persistent respiratory disease correlated with a continuous clinical, physiologic, or other biomarker. The sample size of 750, assuming a missing rate of 10% (5% due to late deaths and 5% due to loss to follow up), allows a power of >80% to detect a significant association at an odds ratio of 1.25 or higher for each one standard deviation increase in the biomarker associated with respiratory disease presence. This calculation assumes no measurement errors and no correlation between the biomarker and other covariates, and a conservative 40% rate of persistent respiratory disease.

#### Clinical data collection

All data are prospectively collected from birth using medical record review and family interviews and include: maternal and infant demographics, clinical data and co-morbidities, daily infant respiratory, nutritional, and medication data throughout the NICU stay. Mothers also provide family history of atopy and asthma (See data collection forms in Additional files 2, 3, 4, 5 and 6). After discharge, a series of telephone questionnaires, based on the Tucson Children’s Respiratory Study [2-6] and the Breathing Outcomes Study (a secondary study to the NICHD Neonatal Research Network Surfactant Positive Airway Pressure and Pulse Oximetry Trial) [7-9] are conducted. The questionnaires assess domains of respiratory morbidity at 3, 6, 9 and 12 months corrected age. At 6 and 12 months, a survey of environmental respiratory irritant exposures and an assessment for gastroesophageal reflux disease, using the modified Infant Gastroesophageal Reflux Questionnaire Revised, are completed [10]. An in-person visit for a physical exam and history is performed at 1 year corrected age. Families are also consented for continuing contact for anticipated longer-term studies.

#### Biospecimen archive

Gaps in our understanding of chronic and long-term respiratory sequelae of prematurity are widened by a general lack of clinically derived biospecimens to be used to identify biomarkers and mechanisms of disease. A sub-committee established standardized procedures for sample collection and central processing, and protocols for accessing the resulting biorepositories (Additional files 1 and 9). Samples collected are cross-sectional or longitudinal (Figure 2). Saliva specimens from infants and parents are collected at study entry for future DNA extraction. Saliva/mouth swabs obtain high quality infant DNA, but may require re-collection to achieve sufficient quantity for exome or genome-wide analyses (>5 micrograms of DNA). Tracheal fluid samples are collected if the infant is intubated and clinically requires suctioning. Tracheal aspirates and urine specimens are obtained on enrollment, 3 days after enrollment, and at 14 and 28 postnatal days. Early (≤1 week) specimens may reflect initial injury, developmental and genetic biosynthetic capacity and present the opportunity to intervene with a targeted therapy, while the later time points (>1 week) may reflect responses to oxidative stress, infection, inflammation, nutritional state, and tissue repair [11]. The DCC maintains details about the biospecimens for quality control and to assist in their identification for distribution. The Steering Committee formally defined a mechanism to submit and evaluate biospecimen access request proposals, with PROP-related investigative teams receiving short-term priority. Applications for access to the specimens will be reviewed for justification and feasibility of the proposed assays, and are expected to demonstrate independent funding to generate and analyze resulting data.

#### Assessments of respiratory function (physiologic biomarkers)

**Figure 3:**
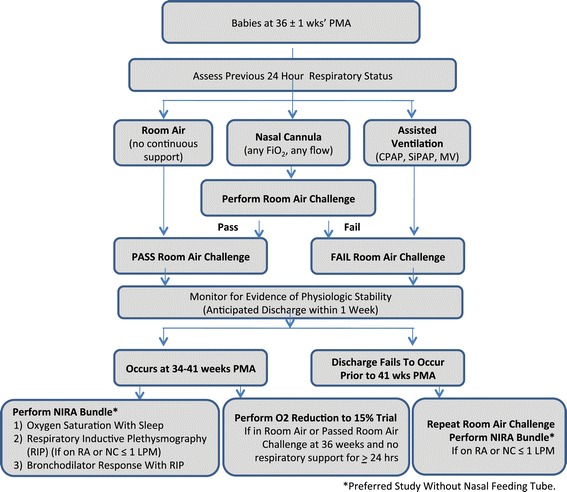
**Non-Invasive Respiratory Assessment (NIRAs) Decision Diagram.** The indicated oxygen reduction tests and respiratory inductive plethysmography (RIP) (with associated tests of oxygen saturations during sleep and oral feeding) were preformed on individual days determined by corrected age, degree of respiratory support required and anticipation of hospital discharge.

A unique feature of the PROP protocol is the inclusion of physiologic biomarkers as potential predictors of respiratory morbidity, based on the established value of premorbid respiratory function as an independent risk factor for wheezing and asthma in later life for term infants [2,5]. Standard infant pulmonary functions tests (PFTs) simulating adult-like spirometry can be performed during the first postnatal year but are not feasible in NICU. Additionally, these tests are time and labor intensive, and not easily performed on a large scale. As alternative measures for the entire cohort, a set of non-invasive respiratory assessments (NIRAs) was selected, including respiratory inductance plethysmography (RIP), pulse oximetry recordings during sleep, bronchodilator response, and oxygen reduction challenges (Figure 3, Table 3). The NIRAs are performed at 34–41 weeks PMA if the subject is not mechanically ventilated or receiving non-invasive positive pressure ventilation. Studies are performed within one week of anticipated discharge and, if possible, without a nasal feeding tube in place. The timing for pre-discharge testing is purposefully linked to anticipated discharge instead of a given gestational age as a reflection of achieved physiologic stability.

**Table 3:** **PROP Noninvasive respiratory assessments (NIRA) and relationship to physiologic measures**

**NIRA**	**Measurements**	**Potential mechanism**
**Respiratory Inductance Plethysmography**	Tidal breathing analysis	Altered lung compliance and airway obstruction
	- Phase angle	
	- Tpef/Te	
	Desaturations after short apnea	Reduced functional residual capacity
	Response to inhaled albuterol	Increased smooth muscle tone
	- Phase angle	
	- Tpef/Te	
**Physiologic Challenges**	Oxygen and flow reduction to ambient air	Alveolar-arterial oxygen gradient, control of breathing, standard definition of “BPD”
	Hypoxic challenge test with 0.15 FiO_2_	V/Q mismatch, control of breathing, “respiratory reserve”

Specific NIRAs performed depend on the clinical status at the anticipated discharge date and include measurements of thoracoabdominal asynchrony and the ratio of time to peak tidal expiratory flow to expiratory time (Tpef/Te) using RIP [12-19]. To assess potential airway reactivity, RIP is performed before and after inhaled albuterol [12,19]. Continuous pulse oximetry data are obtained from quiet sleep during the RIP study and analyzed for the number and pattern of desaturations associated with spontaneous short (<20 sec) apneas. This non-invasive test indirectly infers that lung volume is adequate and can be maintained. Increased frequency of mild oxyhemoglobin desaturations with short apneas can reflect reduced functional residual capacity [19]. The PROP analysis assesses the number of episodes per minute of quiet sleep in which the oxygen saturation (SpO_2_) decreases 4% or more, the fall in SpO_2_ per second of apnea, and the lowest SpO_2_.

For comparison purposes with existing classifications of BPD [19-24], data regarding use of supplemental oxygen at 36 ± 1 weeks PMA and results of a standardized oxygen requirement challenge test are collected. If the infant fails the challenge test or is not eligible based on degree of respiratory support or clinical care team assessment of instability, and if the infant is still in the hospital, another assessment and challenge is attempted at 40 ± 1 weeks (Figure 3). The protocol developed by Walsh, *et al.* was modified to discriminate between effects of FiO_2_ and flow (the latter potentially generating stimulation or positive distending pressure, thus affecting oxygenation [23]). The infants are studied in quiet sleep at least 30 minutes after feeding. If oxygen saturations are maintained ≥90% for at least 15 minutes on the prescribed nasal cannula support, the FiO_2_ is weaned to 0.21 in decrements of 0.2 at 5-minute intervals. The flow is then reduced in 1 liter/min (LPM) decrements at 10-minute intervals until it is less than 1.5 LPM, followed by 50% decrements to a minimum of 0.125 LPM. After a 10-minute observation period, the cannula is removed and the infant is monitored in room air for 1 hour. Failure at any point in the testing is defined as SpO_2_ < 90% for 5 continuous minutes, SpO_2_ < 80% for 15 seconds, or apnea for >20 seconds. The latter, or bradycardia of <80 beats/min for >10 seconds, are recorded as adverse events. The infants are returned to the original support at the end of testing.

To further characterize the respiratory reserve of infants who are breathing ambient air or who pass a room air challenge test at 36 weeks, a 15-minute trial of FiO_2_ of 0.15 (the equivalent of the O_2_ partial pressure at 8000 feet altitude) was originally performed prior to discharge. This “hypoxia challenge” is recommended for individuals with cardiopulmonary conditions in anticipation of air travel [25,26]. After baseline data collection, if SpO_2_ was continuously ≥90%, a monitored respiratory oxyhood is placed over the sleeping infant with either a commercially available mixture of 15% oxygen with nitrogen or an on-site blended mixture of medical oxygen and nitrogen resulting in an FiO_2_ of 0.15. Criteria for failure of this test included SpO_2_ < 85% for 60 consecutive seconds, SpO_2_ < 80% for 15 seconds, bradycardia (HR <80 beats/min for 10 sec) or apnea persistent despite mild tactile stimulation. Interim review of results performed in 2013, demonstrated a test rate of 34% (221 of 643 discharged to home) and a failure rate of 78% with a median age of 36 weeks PMA at testing. Although no significant adverse events occurred, the high failure rate raised concerns about the ability of this challenge to provide additional insight into respiratory physiology and prompted the Observational Safety Monitoring Board to discontinue the hypoxia test in December 2013.

No severe adverse events (need for CPR or mechanical ventilation or increase in FiO_2_ by 0.1 above baseline) have occurred during any of the NIRA assessments. Four mild adverse events occurred during the study: one apnea (>20 seconds) with a RIP, and three bradycardia events, one with the hypoxia challenge and two with the room air challenge. No tachycardia (>200 beats per minute for >1 hour), or need for increase flow or FiO2 above baseline have occurred after any test.

#### Standardized physical examination

The Respiratory Distress Assessment Instrument (RDAI) and the Respiratory Assessment Change Score quantify responses to interventions in infants with wheezing [25-30] with good inter-observer reliability [31]. The PROP Physical Examination Score uses some elements of the RDAI and incorporates other elements that reflect the effects of chronic respiratory impairment, such as growth and the development of digital clubbing. This standardized physical exam focuses on anthropometrics, vital signs, and respiratory system signs and is performed coincident with RIP (36–40 weeks) and again at 1 year corrected age.

Examiners are all trained and certified to perform the standardized exam. With the infant in the supine position and awake, heart rate, respiratory rate and highest sustained oxyhemoglobin saturation are recorded over one minute. If the infant is still requiring supplemental oxygen (≤1.5 LPM) at the 12-month visit, the child is placed in room air for 2 minutes for measurement purposes only. End-inspiratory chest circumference, presence of suprasternal, intercostal or subcostal retractions, thoraco-abdominal asynchrony and accessory muscle use are recorded. Chest auscultation determines the presence, localization, and characteristics of crackles or wheezes. The cardiac point of maximal ventricular impulse is determined by palpation and the presence of digital clubbing is noted. Physical exam components are entered into single point and longitudinal algorithms as a composite score for later analysis.

#### Infant pulmonary function testing (iPFT)

Prematurity is associated with poorer lung function in infancy and childhood: even infants with minimal or no oxygen requirement at discharge demonstrate airflow obstruction and gas trapping compared to full-term controls [32-35]. To evaluate lung growth and respiratory dysfunction, six sites trained and certified to perform iPFTs will test 180 PROP infants. Infants eligible for iPFT receive chloral hydrate sedation to perform tidal breathing, single occlusion resistance/compliance measures, plethysmography and spirometry by the raised volume rapid thoracoabdominal compression (RVRTC) technique using the nSpire Infant Pulmonary Lab (nSpire, Inc, Longmont, CO) or BabyBox device (Carefusion Respiratory Diagnostics,Yoma Linda, CA) [36]. The raised volume technique is repeated after albuterol administration [37]. Measurements are listed in Additional file 8: Table S1. All techniques meet American Thoracic Society/European Respiratory Society standards [36,38-40]. Expert reading of the iPFT recordings is performed at two sites (Indiana University, University of North Carolina at Chapel Hill).

#### Data collection, management and storage systems

A centralized Oracle database system (Oracle Corporation, Redwood Shores, CA) is maintained by the DCC. Local sites contribute data using a web-based interface. Individual centers retain access to their own data through customized downloads from Oracle that are transferred systematically to a REDCap (Research Electronic Data Capture) web-based database management tool [41]. In addition, some centers maintain an independent, customized, local REDCap database that is specific to their single site data acquisition. These data management options provide flexibility, separate layers of data quality control and simple and complete local access to Center specific data. The combination of a centralized database and local tools and storage result in a balance between the needs for a stable and secure core structure and flexible end-user applications.

#### Analytic approaches and considerations

The primary outcome of “post-prematurity respiratory disease” requires that infants demonstrate morbidity in one of the four post NICU discharge domains (respiratory medications; hospitalizations for cardiopulmonary cause; coughing, wheezing, or other respiratory symptoms; or home technology dependence) in at least 2 time frames (at approximately 3 month intervals). Death from a respiratory cause is also included. Secondary analyses will allow the modification of these criteria to assess the sensitivity of the chosen definition for the outcome. Alternate analyses will also consider a repeated measures analysis for the individual-visit level data using mixed effect logistic regression and generalized estimating equations.

Analysis approaches will include multiple individual statistical tests at the univariate and multivariate levels utilizing the accumulated clinical and physiologic data to evaluate potentially complex associations, to control for confounding factors, and to assess contributions to outcomes. Since the PROP is composed of 6 designated clinical research centers affiliated with a total of 13 NICUs, the analysis will be adjusted for clinical sites. The impact of significant missing data on outcomes will be assessed and reported in secondary analyses.

While in some cases it will be appropriate to provide some protection against detecting false positive results, adjustments of significance levels using multiple comparison techniques, which would lessen the chance of detecting potentially important biomarkers and other risk factors for severity of respiratory disease will be used sparingly [42]. These more liberal approaches to dealing with multiple comparisons were considered for hypothesis generating rather than hypothesis testing aims. Therefore, significance values related to odds ratios for biomarker prediction of the one-year outcome will generally be used as indicators for postulated risk factors.

Although PROP clinical sites are dispersed across the country, they may not be representative of NICUs in any defined geographic area. Known and measured factors that might impact the results will be assessed to examine factor imbalances within the PROP cohort and whether factor variations reflect the general population. The sensitivity of our approaches to changes in these factors will allow us to adjust or acknowledge discrepancies in any statements or inferences from PROP.

#### Study approval and oversight

The multi-center PROP protocol and consent forms were evaluated by the University of Pennsylvania IRB (initial approval date July 20, 2011) as a greater than minimal risk protocol due to the oxygen reduction testing and albuterol administration. In addition IRBs at each PROP center determined level of risk, based on their local interpretation. The IRBs further addressed issues of the future use of archived biospecimens, inclusion of an opt-in/opt-out approach for separate components of the protocol, including for DNA collection, and for future research, and methods to ensure subject privacy and protection. The program follows a strict monitoring plan for reporting adverse events and is monitored by an independent Observational and Safety Monitoring Board (OSMB) appointed by the Director of NHLBI. The complete list of IRB approvals and protocol numbers can be found in Appendix 2.

#### Training and quality control

Since study inception, the DCC has held bi-weekly training webinars with the research team from each site to ensure uniform approaches to data and specimen collection and respiratory assessments. Regular survey of the data by the DCC also permits targeted intervention at sites that may be facing challenges in executing some aspects of the protocol. In the early phases of enrollment, site visits by a team that included a PROP Principal Investigator, a consortium-identified lead research coordinator and lead respiratory therapist, pulmonologist, and representatives from NHLBI and the DCC reinforced these procedures and developed best practices to disseminate and maximize uniformity across the consortium, similar to that done with other complex multicenter protocols [43-45].

### Interactions of single center and multicenter protocols

Each of the six non-DCC PROP Study Centers is responsible for a “single center” peer-reviewed project (Table 1). These projects share a common goal of identifying biomarkers of respiratory morbidity over the first year of life and an emphasis on preterm infants <29 weeks gestation. The common database of demographics, clinical NICU events, and respiratory examination and survey results reflecting 1-year outcomes can be used by each single center in the evaluation of their biomarker(s) of choice. Single center projects can recruit additional infants, including some born at >29 weeks and can obtain additional history or biospecimens not collected by the multicenter consortium. The Steering Committee decided that data and specimens relevant to a single center’s studies only would be entered and maintained locally.

### Challenges and resolution

Several questions arose in the development and implementation of the study, all of which were brought to the Steering Committee for discussion and resolution. For example, delays and inconsistency in enrollment across sites prompted extensive examination of consent and enrollment procedures through webinars and site visits. Approaches based on the more successful centers’ practices were implemented and resulted in the targeted enrollment across the consortium.

As PROP investigators continue to formulate new ideas, the issue of who “owns” the multicenter data and specimens has also created tension regarding allocation of resources between the current needs of the PROP, such as analysis of existing data and publication of major outcomes, versus the need for preliminary data to develop funding proposals to pursue these new hypotheses. The Steering Committee decides on an individual basis how to balance these priorities. Access to biospecimens and linked data undergoes a similar review and prioritization process.

The maintenance of data and the biorepository beyond the PROP funding period are currently being resolved. As new funding proposals are developed that utilize collected biospecimens, support for personnel, specimen retrieval, and quality control procedures are being incorporated into the budgets and scientific plans.

## Summary and progress through enrollment

**Figure 4:**
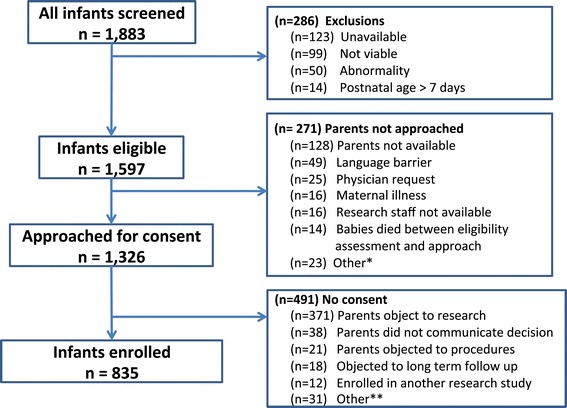
**PROP Consort Diagram.** * ‘Other’ reasons for not approaching parents for consent included maternal age and comprehension (9), parents objected to being approached for research (8), baby transferred less than 7 days (3), and screening oversight (3). ** ‘Other’ reasons for failure to consent included: language barrier (9), parent(s) overwhelmed (8), maternal illness (5), aged out prior to consent (4), baby died between consent discussion and decision (2), maternal comprehension (2), baby Illness (1).

Enrollment began August 3, 2011 and concluded November 1, 2013 (Figure 4); the follow-up to one year corrected age is expected to continue through mid-2015. Consent rates ranged from 42.4% to 80.9% by center, or 63.0% for the consortium, for a total enrollment of 835 participants. The most frequent reasons for exclusion or not approaching parents are highlighted in Figure 4. The demographics of the enrolled cohort are depicted in Table 4 and do not differ significantly from those not enrolled (data not shown).

**Table 4:** **Demographics of enrolled PROP multicenter cohort**

	**Enrolled** ^**a**^ **(N = 835)**
**Birth weight, grams (mean +/− SD)**	900 +/− 240
**Gestational age, weeks (mean +/− SD)**	26.6 +/− 1.5
**- 23 weeks, 0–6 days**	34 (4%)
**- 24 weeks, 0–6 days**	108 (13%)
**- 25 weeks, 0–6 days**	129 (15%)
**- 26 weeks, 0–6 days**	170 (20%)
**- 27 weeks, 0–6 days**	201 (24%)
**- 28 weeks, 0–6 days**	193 (23%)
**Multiple gestation**	210 (25.1%)
**- Twins**	171 (81%)
**- Triplets**	35 (17%)
**- Quadruplets**	4 (2%)
**Male**	427 (51%)
**Race** ^**b**^	
**- Caucasian**	486 (58.2%)
**- African American**	307 (36.8%)
**- N. American Indian/Native Alaskan**	1 (0.1%)
**- Asian**	20 (2.4%)
**- Native Hawaiian/other Pacific islander**	1 (0.1%)
**- Multi-race**	15 (1.8%)
**- Other**	5 (0.6%)
**Ethnicity** ^**b**^	
**- Hispanic/Latino**	92 (11%)
**Inborn**	728 (87.2%)
**Outborn**	105 (12.6%)

^a^Percentages may not add to 100 percent due to missing data for two babies who were withdrawn four days after enrollment.

^b^Race and ethnicity uses a mutually exclusive definition.

The biospecimen archive of DNA, tracheal aspirate and urine specimens, the physiologic testing, and breadth of the investigative teams prompted several ancillary studies that have added dimensions to the original PROP design (Table 5). Most notable was the development of the PROP Scholars’ program through support obtained from the NICHD BPCA program. This program’s major objective is to attract and retain pediatric physician scientists who are interested in pulmonary investigation. Through small competitive grants, trainees and non-NIH-funded junior faculty have access to learning opportunities otherwise not available and develop a time-limited research project that augments PROP single site studies (Table 5). Over 3 years, 12 individuals have been funded and afforded the opportunity to present their work at in-person steering committee meetings, as well as at national pediatric and pulmonary research meetings.

**Table 5:** **Ancillary projects arising from PROP**

**Project**	**Center**	**Publications**
**PROP Scholars**		
Preventing attrition in follow-up	Penn	
Inter-rater reliability in physical exam	Penn	
Tracheal aspirate connective tissue mast cells in BPD	Rochester	[45]
Training in DLCO measurements	Rochester	
Treg impairment in preterm infants exposed to chorioamnionitis	Cincinnati	
Glutathionated hemoglobin as a biomarker for oxidant stress	Vanderbilt	[46]
RAGE signaling in BPD	Vanderbilt	
Periostin levels as a biomarker for BPD	Indiana	
Right ventricular strain measurements in evolving BPD	Washington U	[47-50]
Genetic contributions to BPD	UCSF	
Longitudinal lung clearance index measurements in premature infants	UCSF	
Oxidant stress and neurodevelopmental outcomes	Vanderbilt	
Variability in RIP	Washington U	[51]
Relationship between feeding desaturation, respiratory function and one year respiratory outcome in preterm infants	Rochester	
Respiratory pattern during physiologic challenges	Washington U	
RIP late preterm and term newborns	Rochester, Washington U	
Genetic Variants and Bronchopulmonary Dysplasia in Premature Infants	Multi-center	

## Discussion

In summary, the current dissociation between the diagnosis of BPD and respiratory outcomes/pathogenesis of disease is due in part to a focus on hospitalization characteristics rather than longer-term outcomes. In reality, the continuum of disease and outcomes extend far beyond the neonatal period, and it is in this context that the respiratory consequences of prematurity need to be defined and evaluated. The PROP represents a significant investment by NHLBI to combine the multidisciplinary expertise of neonatologists, pediatric pulmonologists and basic scientists to provide a balanced analysis of disease and care of pulmonary disease of prematurity throughout the first year of life. The PROP will provide an understanding of mechanisms, evolution and consequences of lung disease in these preterm infants. It will also create a repository of genetic and biological samples that will provide the seeds of new hypotheses and future research in the complexity of gene-environment interactions.

## Appendix 1 Investigators and Research Staff

### Cincinnati Children’s Hospital Medical Center

#### Investigators

Claire Chougnet, PhD

Robert Frenk, MD

James M. Greenberg, MD

William Hardie, MD

Alan H. Jobe MD, PhD

Karen McDowell, MD

#### Research staff

Barbara Alexander, RN

Tari Gratton, PA

Cathy Grigsby, BSN, CCRC

Beth Koch, BHS, RRT, RPFT

Kelly Thornton BS

### Washington University School of Medicine site

#### Investigators

Thomas Ferkol, MD

Aaron Hamvas, MD^2^

Mark R. Holland, PhD

James Kemp, MD

Philip T. Levy, MD

Phillip Tarr, MD

Gautam K. Singh, MD

Barbara Warner, MD

#### Research staff

Pamela Bates, CRT, RPFT, RPSGT

Claudia Cleveland, RRT

Julie Hoffmann, RN

Laura Linneman, RN

Jayne Sicard-Su, RN

Gina Simpson, RRT, CPFT

^2^Northwestern University Feinberg School of Medicine

### University of California San Francisco site

#### Investigators

Philip L. Ballard MD, PhD

Roberta A. Ballard MD

David J. Durand MD^2^

Eric C. Eichenwald MD ^4^

Roberta L. Keller MD

Amir M. Khan MD^4^

Leslie Lusk MD

Jeffrey D. Merrill MD ^3^

Dennis W. Nielson MD, PhD

Elizabeth E. Rogers MD

#### Research staff

Jeanette M. Asselin MS RRT-NPS^2^

Samantha Balan

Katrina Burson RN, BSN ^4^

Cheryl Chapin

Erna Josiah-Davis RN, NP^3^

Carmen Garcia RN, CCRP ^4^

Hart Horneman

Rick Hinojosa BSRT, RRT, CPFT-NPS ^4^

Christopher Johnson MBA, RRT ^4^

Susan Kelley RRT

Karin L. Knowles

M. Layne Lillie, RN, BSN ^4^

Karen Martin RN ^4^

Sarah Martin RN, BSN;

Julie Arldt-McAlister RN, BSN ^4^

Georgia E. McDavid RN ^4^

Lori Pacello RCP^2^

Shawna Rodgers RN, BSN ^4^

Daniel K. Sperry RN ^4^

^2^Children’s Hospital and Research Center Oakland, Oakland CA

^3^Alta Bates Summit Medical Center, Berkeley CA

^4^University of Texas Health Science Center- Houston, Houston TX

### Vanderbilt University Medical Center site

#### Investigators

Judy Aschner, MD^2^

Candice Fike, MD

Scott Guthrie, MD^3^

Tina Hartert, MD

Nathalie Maitre, MD

Paul Moore, MD

Marshall Summar, MD^4^

#### Research Staff

Amy B Beller BSN

Mark O’ Hunt

Theresa J. Rogers, RN

Odessa L. Settles, RN, MSN, CM

Steven Steele, RN

Sharon Wadley, BSN, RN, CLS^3^

^2^Albert Einstein College of Medicine, Bronx NY

^3^Jackson-Madison County General Hospital, Jackson TN

^4^Children’s National Health System, Washington DC

### University of Rochester Medical Center/University at Buffalo NY site

#### Investigators

Carl D’Angio, MD

Vasanth Kumar, MD

Tom Mariani, PhD

Gloria Pryhuber, MD

Clement Ren, MD

Anne Marie Reynolds, MD, MPH

Rita M. Ryan, MD^2^

Kristin Scheible, MD

Timothy Stevens, MD, MPH

#### Research staff

Shannon Castiglione, RN

Aimee Horan, LPN

Deanna Maffet, RN

Jane O’Donnell, PNP

Michael Sacilowski, MAT

Tanya Scalise, RN, BSN

Elizabeth Werner, MPH

Jason Zayac, BS

Heidie Huyck, BS

Valerie Lunger, MS

Kim Bordeaux, RRT

Pam Brown, RRT

Julia Epping, AAS, RT

Lisa Flattery-Walsh, RRT

Donna Germuga, RRT, CPFT

Nancy Jenks, RN

Mary Platt, RN

Eileen Popplewell, RRT

Sandra Prentice, CRT

^2^Medical University of South Carolina, Charleston SC

### Duke

#### Investigators

C. Michael Cotten, M.D.

Kim Fisher, Ph.D.

Jack Sharp, M.D.

Judith A. Voynow, M.D. ^2^

#### Research staff

Kim Ciccio, RN

^2^Virginia Commonwealth University

### Indiana

#### Investigators

Stephanie Davis, MD

Brenda Poindexter, MD, MS ^2^

#### Research staff

Charles Clem, RRT

Susan Gunn, NNP, CCRC

Lauren Jewett, RN, CCRC

^2^Cincinnati Children’s Hospital Medical Center

### University of Pennsylvania, Perelman School of Medicine, DCC site

#### Investigators

Jonas Ellenberg, PhD

Rui Feng, PhD

Howard Panitch, MD

Barbara Schmidt MD, MSc

Pamela Shaw, PhD

#### Research staff

Maria Blanco, BS

Denise Cifelli, MS

Sara DeMauro, MD

Melissa Fernando, MPH

Ann Tierney, BA, MS

### University of Denver, Steering Committee Chair

Lynn M. Taussig, MD

#### NHLBI program officer

Carol J. Blaisdell, MD

## Appendix 2

### Institutional Review Board Protocols at individual PROP sites

1. The University of Pennsylvania Data Coordinating Center – 813839
2. Cincinnati University Hospital (11) - 2011-1664
3. Cincinnati Children’s Hospital Medical Center (12) – 2011-1664
4. Cincinnati Good Samaritan’s Hospital (13) – 11139-11-084
5. Washington University (21) – 201104267
6. University of California, San Francisco (31) – 10-04838
  - Alta Bates (32) – 11-08-03
  - CHO (32) –2011-050
7. University of Texas, Houston (33) – HSC-MC-11-0499
8. Vanderbilt University Monroe Caroll (41) - 110833
9. Jackson Madison County General Hospital (42) – 542
10. University of Rochester (51) – RSRB00037933
11. University of Buffalo (52) – 386479-1 DB # 2707
12. Duke University (61) – Pro 00025462
13. Indiana University (62) - 1201007831

## Additional files

Additional file 1:
**PROP biorepository lab specimen collection guideline.**
Additional file 2:
**Prematurity and respiratory outcomes program.** Eligibility and baseline data. Additional file 3:
**Prematurity and respiratory outcomes program.** Daily growth and nutrition/daily medication data. Additional file 4:
**Prematurity and respiratory outcomes program.** Comorbidities of prematurity. Additional file 5:
**Prematurity and respiratory outcomes program.** Standard visit and follow up surveys. Additional file 6:
**Prematurity and respiratory outcomes program.** Additional medication log and miscellaneous forms. Additional file 7:
**Prematurity and respiratory outcomes program.** Noninvasive respiratory assessments [NIRA]. Additional file 8: Table S1. Infant pulmonary function test measurements. Additional file 9:
**PROP biospecimen access policy.**

## Abbreviations

BPD: Bronchopulmonary dysplasia
DCC: Data coordinating center
FiO_2_: Fraction of inspired oxygen
iPFT: Infant pulmonary functions tests
IRB: Institutional review board
LPM: Liters per minute
NHLBI: National Heart, Lung, and Blood Institute
NICHD: National Institute of Child Health and Human Development
NICU: Neonatal Intensive Care Unit
NIH: National Institutes of Health
NIRA: Non-invasive respiratory assessments
OR: Odds ratio
OSMB: Observational safety monitoring board
PI: Principal investigator
PMA: Post menstrual age
PMI: Point of maximal ventricular impulse
PROP: Prematurity and respiratory outcomes program
RDAI: Respiratory distress assessment instrument
REDCap: Research electronic data capture
RIP: Respiratory inductance plethysmography
RVRTC: Raised volume rapid thoracoabdominal compression
SC: Steering committee
SpO_2_: Oxygen saturation
Tpef/Te: Ratio of time to peak tidal expiratory flow to expiratory time
