# Supplementary material for: Prematurity and respiratory outcomes program (PROP): study protocol of a prospective multicenter study of respiratory outcomes of preterm infants in the United States
Source: BMC Pediatr. 2015 Apr 10;15:37. doi: 10.1186/s12887-015-0346-3 (PMC4407843; doi:10.1186/s12887-015-0346-3)
Supplement: Additional file 1: — PROP biorepository lab specimen collection guideline. [file 12887_2015_346_MOESM1_ESM.pdf]

PROP BIOREPOSITORY LAB SPECIMEN COLLECTION GUIDELINE

Revised 2/4/13

SALIVA for DNA

Specimen & Schedule

- Collect saliva from infant, mother and, if possible, the father.
- The collection should be made within the first week after enrollment, if possible, or otherwise before discharge or expected demise.
- Use Oragene® kits provided.

Processing & Storage

- **For infants**, follow the instructions using the **OG250** kit and 5 swabs altogether: two at a time in each side of the mouth and then one final swab to soak up saliva. Obtain infant specimens just before feeding.
- **For parents**, follow the instructions in the **OG500** kit for collection of saliva by spitting.

1. Confirm that the top is screwed tightly to limit evaporation.
2. Fill out the label on the kit with "PROP", PID number, source of saliva (ie., infant, mother or father) and date using a water-resistant (permanent) Sharpie pen.
3. Enter the sample collection information into the CRF Biorepository Specimen Collection form.
4. Store the kit with the saliva sample at room temperature.

Shipping

1. Place all kits into a box and include in the box the packing list for saliva samples.
   Keep a copy of the form for your records.
   Ship to: **VUMC - DNA Resources Core**
   1161 21st Avenue South
   MCN T - 0102
   Nashville, TN 37232
   Phone: 615-322-4277; Cara Sutcliffe (cara.b.sutcliffe@vanderbilt.edu)
   This address is correct for UPS, FedEx or Airborne deliveries, but ***FedEx is recommended***.
2. Request a "**clinical overwrap**" from FedEx for the box.
3. ***Note:*** Ship monthly (or when a reasonable number of specimens is obtained) at **ambient** temperature on **Monday-Wednesday ONLY**. Record shipment date and FedEx # on Sample Shipment Log.
4. Contact the **DNA Resources Core** by phone, email or fax when you are shipping samples.
   Phone: **615-936-2744** (there is a voice mail option when you call outside of normal hours).
   Email: .dnacore@list.vanderbilt.edu or Fax: **615-343-8619 – Attention: DNA Resources Core**

URINE

Specimen & Schedule

Site-specific collection kits provided by UCSF. Obtain urine samples on all PROP infants.

#1) ASAPAE (as soon as possible after enrollment)	#3) day 14 (±1) of life
#2) 3 (±1) days later	#4) day 28 (±1) of life

Processing & Storage

1. Place 2 cotton balls in diaper.
2. After voiding, insert cotton balls into 10 mL syringe with plunger removed. (Do not use cotton balls contaminated with stool.)
3. Insert and depress syringe plunger and squeeze out as much urine as possible into the 4-ml tube. At least a 1 mL is needed; 2 ml is preferable. If you require multiple voids to collect ≥1 ml of urine, place labeled tube in specimen refrigerator between collections. For multiple voids, indicate the last collection date which is when the sample will be processed. Confirm that the collection tube top is screwed tightly to minimize evaporative loss.
4. Gently mix the urine by inversion and then aliquot 0.25 ml of urine into each of the 1-ml labeled cryo vials with final numbers of 01, 02, 03, 04. Transfer the remaining urine into the labeled vial with final number 05.
   (If there is residual urine after the aliquoting, discard or keep on-site as needed.)
5. Replace tops on vials---screw tightly to limit evaporation.
6. Place vials in the Biosafety bag and write the patient ID # and date on the label using a Sharpie pen.
7. Freeze specimens at -70° C (-20° C is acceptable until freezer space is available).
8. Enter sample collection information into the CRF Biorepository Specimen Collection form posted on the website.
   Verify that all information is recorded correctly. Enter box number at time of shipment
   (or when samples are transferred to freezer boxes).

Shipping

1. If your site is performing assays on urine, you may retain vial 05 and, if needed, vial 04 on-site.
2. Transfer sample tubes from sample bags to freezer storage boxes for shipping.
   Place freezer boxes in shipping container then add dry ice (approximately 1/3 container volume).
3. Include in the box the packing list for the enclosed samples and keep a copy of the form for your records. Ship the samples via Fedex priority overnight delivery to the address below. The prepaid **FedEx shipping number is 308893197**.
4. Notify Mark Hunt at address below prior to shipment and email him a copy of the revised shipping list which should contain Fedex tracking information, date of shipment, number of samples, etc.
3. Send all samples from a given infant in the same shipment. You may hold samples until the freezer box is full or nearly full.
4. Ship **Monday-Wednesday ONLY** to: **Judy Aschner, MD [ATTN: Mark Hunt] (mark.o.hunt@vanderbilt.edu)**
   Vanderbilt University Medical Center 1125 Light Hall, 2215 B Garland Avenue
   Nashville, TN 37232-0656
   Phone: 615-343-0443

TRACHEAL ASPIRATE (TA)

Specimen & Schedule

- Site-specific collection kits provided by UCSF. Obtain TA samples on all intubated PROP infants.
- Perform only if infant is stable and will tolerate suctioning.
- Obtain sample during routine suctioning using the standard protocol at your unit.

#1) ASAPAE (as soon as possible after enrollment) #3) day 14 (±1) of life
#2) 3 (±1) days later #4) day 28 (±1) of life

Processing & Storage

1. Use a new (sterile) suction catheter and the Leukens trap provided in the collection kit.
2. Draw 2.0 ml of NSS into sterile syringe.
3. Instill 0.5 ml of NSS into the ET.
4. Ventilate lungs as per usual protocol at your unit prior to suctioning.
5. Suction the effluent into the Leukens trap. Be sure that the catheter tip does not extend beyond the tip of the ET.
6. Stabilize the infant and then repeat steps 3, 4, and 5.
7. Rinse the suction catheter with remaining NSS in the syringe. This represents a single sample, which should be ~1.5 -2.5 ml.
   NOTE - If preferred, suctioning may be done without NSS instillation. In that case, rinse catheter into trap with 2 ml of NSS after suctioning.
8. Attach ends of Leukens tubing together to keep sample clean. Write patient ID # and date on trap label.
9. If more than 5 minutes will elapse before processing, place the trap on ice or in refrigerator until processing.
   Do not freeze before processing. Process within 6 hours if possible.
10. Apply suction to the trap to completely empty the tubing. Pour entire contents of the Leukens trap into the 4-ml “CL” tube provided in the kit. Discard the empty trap.
12. Centrifuge sample at ~3000 rpm for 5 min.
13. Separate the supernatant from the cells by decanting supernatant into the other 4-ml tube (supernatant), obtaining last drop by touching edges of two tubes.
14. Using the plastic disposable pipette, remove and discard any clumped, mucous material from the supernatant. (If needed, cut off tip of pipette for a wider opening.) Remaining volume should be > 1 ml.
15. Gently mix the supernatant by inversion and then aliquot 0.25 ml into each of the 1-ml labeled cryo vials with final numbers of 01, 02, 03, 04. Transfer the remaining supernatant into the cryo vial with final number 05.
16. Replace tops on vials---screw tightly to limit evaporation. Place vials and CL tube in the Biosafety bag and write the patient ID # and date on the label using a Sharpie pen.
17. Freeze specimens at –70 °C (preferable), -20° C (if necessary) in the plastic bag provided with the vials in the upright position if possible. ***Discard the Leukens trap*** and the empty 4-ml tube.
18. Enter sample collection information into CRF Biorepository Specimen Collection form provided from the website.

Shipping

1. If your site is performing assays on TA, you may retain vial 05 and, if needed, vial 04 on-site.
2. Place bags with samples in a foam box (ThermoSafe* Multipurpose Insulated Shippers, see below) on a layer of dry ice. Fill box to the top with more dry ice. The total amount of dry ice should be about half the volume for a smaller box and 1/3 of the volume for a larger box.
3. Ship by overnight express to the address below. Include in the box the packing list for the enclosed samples and keep a copy of the form for your records. ***Do not send Leukens trap***.
4. Email **Cheryl Chapin** (**Cheryl.chapin@ucsf.edu**) to obtain prepaid FedEx shipping number.
   1. Record shipment date and FedEx number on your Sample Shipment Log.

Note: Ship samples (at least 6 infants) to UCSF, **Monday-Wednesday ONLY**.

Address to: **Cheryl Chapin**
3333 California St., Suite 150
San Francisco, CA. 94118
415-476-2535

ADDITIONAL INFORMATION	UCSF Contact (TA)	Vanderbilt Contact
Enter sample collection information into SPEC form posted on the website.	Cheryl Chapin (Cheryl.chapin@ucsf.edu)	**(Urine)** Mark Hunt (mark.o.hunt@vanderbilt.edu)<br>**(DNA)** dnacore@chgr.mc.vanderbilt.edu.

Supplies and Equipment Needed:

NEW FREEZER BOXES (Urine shipment) :

Fisher Scientific .... (1-800-766-7000)....... Catalog # 03-395-464
Denville Scientific ...... (1-800-453-0385)....... Catalog # R3030-81
VWR .... (1-800-932-5000)....... Catalog # 82007-162
USA Scientific .... (1-800-522-8477)....... Catalog # 9023-4981

- ThermoSafe* Multipurpose Insulated Shippers
- Fisher Scientific #03-530-17 (urine and TA)
- cardboard box (saliva) for shipping
- Pipette and tips for 250µl aliquoting
- Test tube rack
- Clinical centrifuge for 4 ml tubes
