# Supplementary material for: Prematurity and respiratory outcomes program (PROP): study protocol of a prospective multicenter study of respiratory outcomes of preterm infants in the United States
Source: BMC Pediatr. 2015 Apr 10;15:37. doi: 10.1186/s12887-015-0346-3 (PMC4407843; doi:10.1186/s12887-015-0346-3)
Supplement: Additional file 2: — Prematurity and respiratory outcomes program. Eligibility and baseline data. [file 12887_2015_346_MOESM2_ESM.pdf]

1. What is the baby's gender? ☐1 Male ☐2 Female
2. What is the baby's birth weight? __ __ __ __ gms
3. What is the baby's head circumference? __ __ . __ cm
4. What was the baby's birth location? ☐1 Born inside the study center ☐2 Born outside the study center
5. What was the baby's expected date of birth (EDC)? __ __ / __ __ / __ __ __ __ (Month / Day / Year)
6. Was this a multiple birth? ☐0 No ☐1 Yes
   If Yes, answer questions 6a and 6b.
   6a. Indicate the baby's birth order: __ Number of __ Number
   6b. Record the PID(s) of siblings enrolled in the PROP multicenter core database:
   PID 1: __ __ __ __ __ __ __
   PID 2: __ __ __ __ __ __ __
7. Were umbilical cord blood gas analyses performed? ☐0 No ☐1 Yes

   If **Yes**, record the result(s) of the umbilical cord blood gas analysis:
   7a. Sample pH: __ . __ __ | Base Deficit: __ __ . __
   Type of sample: ☐1 Venous ☐2 Arterial ☐3 Uncertain

   7b. Sample pH: __ . __ __ | Base Deficit: __ __ . __
   Type of sample: ☐1 Venous ☐2 Arterial ☐3 Uncertain
8. What was the Apgar Score? __ __ [0-10] at 1 minute __ __ [0-10] at 5 minutes
9. Were any stabilization procedures provided at birth? ☐0 No ☐1 Yes

   9a. If **Yes**, check all that apply:
   - ☐1 Supplemental O_2
   - ☐1 CPAP
   - ☐1 Non-invasive positive pressure ventilation with flow inflating or self inflating bag
   - ☐1 T-Piece resuscitator
   - ☐1 Intubation
   - ☐1 Chest compression
   - ☐1 Cardiac drugs (Epinephrine)
   - ☐1 Surfactant administration
10. What was the first temperature recorded at the first NICU admission? __ __ . __ ° C

    10a. Indicate how the temperature was obtained:
    - ☐1 Core temperature (e.g. rectal), or
    - ☐2 Peripheral / skin temperature (e.g. axillary)
11. Was Prophylactic Indomethacin given within the first 24-hours of life? ☐0 No ☐1 Yes

1. Indicate the baby's data set (check all that apply): ☐ PROP Core Database ☐ Single-Center Data Set

2. What is the baby's date of birth? __ __ / __ __ / __ __ __ __ (Month Day Year)

3. What is the baby's time of birth? __ __ __ __ (24 Hour Clock)

4. Gender: ☐1 Male ☐2 Female

5. Infant Ethnicity (reported by parent) ☐1 Hispanic/Latino ☐2 Not Hispanic/Latino

6. Infant Race (reported by parent- check all that apply)
   - ☐ North American Indian/Native Alaskan
   - ☐ Asian
   - ☐ Black / African American
   - ☐ Native Hawaiian / Other Pacific Islander
   - ☐ White / Caucasian
   - ☐ Other: ____________________________

Record all babies who fulfill the single center inclusion criteria and/or both of the PROP Inclusion Criteria below.

Inclusion Criteria:

7. Is the baby's Gestational Age (GA) between 23 weeks and 0/7 days and 28 weeks and 6/7 days? ☐0 No ☐1 Yes

7a. Indicate the number of completed weeks and the number of completed days: __ __ Weeks __ __ Day

7b. What method was used to determine the Gestational Age of the baby?
   - ☐1 Early dating ultrasound (<20 weeks)
   - ☐2 Certain LMP date (if early dating ultrasound is not available)
   - ☐3 Best clinical estimate (if certain LMP date is not available)

8. Is the baby's postnatal age less than or equal to 7 Days? ☐0 No ☐1 Yes

Exclusion Criteria:

9. Is the baby considered not to be viable (decision not to administer effective therapies)? ☐0 No ☐1 Yes

10. Does the baby have congenital heart disease (not including PDA and hemodynamically insignificant VSD or ASD)? ☐0 No ☐1 Yes

11. Does the baby have any structural abnormalities of the upper airway or lungs? ☐0 No ☐1 Yes

12. Does the baby have any other congenital malformations or syndromes that adversely affect life expectancy or development? ☐0 No ☐1 Yes

13. Is the baby unlikely to be available for long-term follow-up? ☐0 No ☐1 Yes

Eligibility:

14. Does the baby meet both of the inclusion criteria and none of the exclusion criteria and is therefore eligible for the Core Database? ☐0 No ☐1 Yes

Complete the Consent Section only for babies who are eligible for the PROP Multi-site Core Database.

Consent:

15. Were the parents of the baby approached about the study? ☐0 No ☐1 Yes

15a. If **No**, select the primary response that best explains why the parents were not approached:

- ☐1 Research staff was not available
- ☐2 Parents were not available
- ☐3 Screening oversight
- ☐4 On request of responsible physician
- ☐98 Other, specify: ________________________________

15b. If **Yes**, was parental consent obtained? ☐0 No ☐1 Yes

15c. If Question 15 b is **No**, select the primary response that best explains why consent was not obtained:

- ☐1 Parents object to participation in research studies
- ☐2 Baby was enrolled in another research study
- ☐3 Parents objected to long term follow-up
- ☐98 Other, specify: ________________________________

16. Was parental consent obtained for DNA sample collections? ☐0 No ☐1 Yes

16a. If **Yes**, indicate which samples were given consent:
(check all that apply)

- ☐ Infant
- ☐ Mother
- ☐ Father

Enrollment:

17. Was the baby enrolled into the study? ☐0 No ☐1 Yes

17a. If **Yes**, enter Date of Enrollment: ______ /______ /______ (Month / Day / Year)

The date of enrollment is the date on which the parent is notified that the baby has been enrolled, after documentation of parental consent and re-confirmation of the baby's eligibility for the multicenter PROP study.

MATERNAL BASELINE DATA

DATE:__ __ / __ __ / __ __ __ __

Maternal Demographic Data:

1. What is the mother's date of birth? __ __ / __ __ / __ __ __ __ (Month / Day / Year)

2. What is the mother's ethnicity?
   - ☐1 Hispanic / Latino
   - ☐2 Not Hispanic / Latino

3. What is the mother's race (check all that apply)?
   - ☐ North American Indian / Native Alaskan
   - ☐ Asian
   - ☐ Black / African American
   - ☐ Native Hawaiian / Other Pacific Islander
   - ☐ White / Caucasian
   - ☐ Other: ______________________________

4. What is the mother's highest level of education?
   - ☐1 Less than 7th grade
   - ☐2 7th – 9th grade
   - ☐3 10th – 12th grade
   - ☐4 High school degree
   - ☐5 Partial college
   - ☐6 College degree
   - ☐7 Graduate degree
   - ☐88 Unknown

5. What is the family arrangement?
   - ☐1 Single parent family
   - ☐2 Two parent family
   - ☐88 Unknown

Paternal Demographic Data:

6. Was demographic data about the **biological father** obtained? ☐0 No ☐1 Yes

If Yes, please complete questions #7-9, regarding the **biological father.**

7. What is the father's ethnicity?
   - ☐1 Hispanic / Latino
   - ☐2 Not Hispanic / Latino

8. What is the father's race (check all that apply)?
   - ☐ North American Indian / Native Alaskan
   - ☐ Asian
   - ☐ Black / African American
   - ☐ Native Hawaiian / Other Pacific Islander
   - ☐ White / Caucasian
   - ☐ Other: ______________________________

9. What is the father's highest level of education?
   - ☐1 Less than 7th grade
   - ☐2 7th – 9th grade
   - ☐3 10th – 12th grade
   - ☐4 High school degree
   - ☐5 Partial college
   - ☐6 College degree
   - ☐7 Graduate degree
   - ☐88 Unknown

Pregnancy History:

10. Did the mother have diabetes during pregnancy? ☐$_0$ No ☐$_1$ Yes
    10a. If Yes, did she receive insulin for her diabetes? ☐$_0$ No ☐$_1$ Yes

11. Did the mother have hypertension during pregnancy? ☐$_0$ No ☐$_1$ Yes
    11a. If Yes, did she receive medication to treat her hypertension? ☐$_0$ No ☐$_1$ Yes

12. Did the mother have asthma during pregnancy? ☐$_0$ No ☐$_1$ Yes
    12a. If Yes, did she take medication regularly to control her asthma? ☐$_0$ No ☐$_1$ Yes

13. Did the mother take any medications to prolong pregnancy? ☐$_0$ No ☐$_1$ Yes

    13a. If Yes, check all that apply:
    - ☐ Progesterone
    - ☐ Cyclooxygenase inhibitors (Indocin)
    - ☐ Oral betamemetics
    - ☐ Calcium Channel Blockers
    - ☐ Oxytocin receptor antagonist
    - ☐ Magnesium Sulfate
    - ☐ Other: ______________________

14. Was a maternal or neonatal toxicology screen performed at the time of birth? ☐$_0$ No ☐$_1$ Yes

    14a. If Yes, indicate the results: ☐$_0$ Negative ☐$_1$ Positive

    14b. If **Positive**, indicate the substances (check all that apply):
    - ☐ Amphetamines
    - ☐ Cocaine
    - ☐ Opiates
    - ☐ Barbiturates
    - ☐ Benzodiazepines
    - ☐ Phencyclidine (PCP)
    - ☐ Tetrahydrocannabinol (THC)
    - ☐ Ethanol
    - ☐ Methadone
    - ☐ Other: ______________________

15. Did the mother smoke tobacco products during pregnancy? ☐$_0$ No ☐$_1$ Yes

    15a. Did anyone else smoke tobacco regularly in the mother's home during her pregnancy? ☐$_0$ No ☐$_1$ Yes

16. Indicate the mother's height and weight at the time of delivery:
    Height: __ __ __ cm ☐$_{88}$ Unknown
    Weight: __ __ __ . __ kg ☐$_{88}$ Unknown

Labor and Delivery Data:

17. Was there placental abruption? ☐$_0$ No ☐$_1$ Yes

18. Did the membrane rupture > 18 hours before delivery? ☐$_0$ No ☐$_1$ Yes

    18a. If Question 18 is **Yes**, did the membrane rupture more than 7 days before delivery? ☐$_0$ No ☐$_1$ Yes ☐$_{88}$ Unknown

19. Was there any clinical chorioamnionitis? ☐$_0$ No ☐$_1$ Yes

    19a. If **Yes**, was placental pathology obtained? ☐$_0$ No ☐$_1$ Yes

    19b. If Question 19a is **Yes**, was there histologic evidence of chorioamnionitis? ☐$_0$ No ☐$_1$ Yes

20. Were antibiotics given? ☐$_0$ No ☐$_1$ Yes

    20a. If **Yes**, why were antibiotics given (check all that apply)?
    - ☐ Chorioamnionitis
    - ☐ Group B Streptococcus (GBS) prophylaxis
    - ☐ Preterm labor
    - ☐ Other: ______________________________

21. Were antenatal corticosteroids given? ☐$_0$ No ☐$_1$ Yes

    21a. If **Yes**, total number of completed courses:
    - ☐$_0$ None
    - ☐$_1$ One course
    - ☐$_2$ Two courses
    - ☐$_3$ Three courses
    - ☐$_4$ Four courses
    - ☐$_5$ Five courses

    21b. Number of incomplete courses:
    - ☐$_0$ None
    - ☐$_1$ One or more courses

22. Was magnesium sulfate given for any reasons other than tocolysis? ☐$_0$ No ☐$_1$ Yes

    22a. If **Yes**, check the primary indication:
    - ☐$_1$ Preeclampsia / eclampsia
    - ☐$_2$ Prevention of Cerebral Palsy

23. Was the onset of labor spontaneous? ☐$_0$ No ☐$_1$ Yes

24. What was the mode of delivery?
    - ☐$_1$ Vaginal Vertex
    - ☐$_2$ Vaginal Breech
    - ☐$_3$ Caesarian Section
