# Supplementary material for: Prematurity and respiratory outcomes program (PROP): study protocol of a prospective multicenter study of respiratory outcomes of preterm infants in the United States
Source: BMC Pediatr. 2015 Apr 10;15:37. doi: 10.1186/s12887-015-0346-3 (PMC4407843; doi:10.1186/s12887-015-0346-3)
Supplement: Additional file 3: — Prematurity and respiratory outcomes program. Daily growth and nutrition/daily medication data. [file 12887_2015_346_MOESM3_ESM.pdf]

Daily Growth and Nutrition Data

For question 1 and 2: If more than one measurement is taken on the same day, enter the first value obtained.

1. What was the baby's body weight? __ __ __ __ gms ☐99 Not done
2. What was the baby's head circumference? __ __ . __cm ☐99 Not done
3. How much milk did the baby receive today?
   - ☐0 None
   - ☐1 Partial Feed
   - ☐2 Full Feed (no parenteral nutrition on this day)

   3a. If **Partial** or **Full** milk feed, indicate the type of milk provided:
   - ☐1 Human milk
   - ☐2 Formula
   - ☐3 Both

Daily Medication Data

4. Were any drugs given today? ☐0 No ☐1 Yes

   If **Yes**, indicate which of the following drugs were given today:

5. Methylxanthine drugs: ☐0 No ☐1 Yes

   5a. If **Yes**, choose one:
   - ☐1 Caffeine Citrate* Dose: _____ mg Given every __ __ hour(s) Route: ☐1 PO (oral) ☐2 IV (intravenous)
   - ☐2 Aminophylline Dose: _____ mg Given every __ __ hour(s)
   - ☐3 Theophylline Dose: _____ mg Given every __ __ hour(s)
   - ☐98 Other (*record on the Additional Medication Log*)

   * If your site calculates the dose of Anhydrous Caffeine Base rather than Caffeine Citrate, multiply the Caffeine Base dose by 2.

6. Systemic Corticosteroid drugs: ☐0 No ☐1 Yes

6a. If **Yes**, check all that apply:
- ☐ Hydrocortisone Dose: _____ mg Given every __ __ hour(s) Route: ☐1 PO (oral) ☐2 IV (intravenous)
- ☐ Dexamethasone Dose: _____ mg Given every __ __ hour(s) Route: ☐1 PO (oral) ☐2 IV (intravenous)
- ☐ Prednisone / Prednisolone Dose: _____ mg Given every __ __ hour(s)
- ☐ Methylprednisolone Dose: _____ mg Given every __ __ hour(s)
- ☐ Other (*record on the Additional Medication Log*)

NOTE: If utilizing a combination therapy drug, record the individual components in their respective drug categories. For example Symbicort (budesonide and formoterol) would be recorded as budesonide – Inhaled Steroid and formoterol – Inhaled Bronchodilator.

7. Inhaled Steroid drugs: ☐0 No ☐1 Yes

7a. If **Yes**, check all that apply:

- ☐ Budesonide (Nebulized or MDI) Dose: _____ ☐ mcg ☐ mg Given every __ __ hour(s)
- ☐ Beclomethasone (MDI) Dose: _____ mcg Given every __ __ hour(s)
- ☐ Ciclesonide (MDI) Dose: _____ mcg Given every __ __ hour(s)
- ☐ Flunisolide (MDI) Dose: _____ mcg Given every __ __ hour(s)
- ☐ Fluticasone (MDI) Dose: _____ mcg Given every __ __ hour(s)
- ☐ Mometasone (MDI) Dose: _____ mcg Given every __ __ hour(s)
- ☐ Triamcinolone (MDI) Dose: _____ mcg Given every __ __ hour(s)
- ☐ Dexamethasone (Nebulized) Dose: _____ mg Given every __ __ hour(s)
- ☐ Other (*record on the Additional Medication Log*)

If route of administration is Metered Dose Inhalator (MDI), calculate dose by multiplying the number of puffs by strength per puff.

8. Inhaled Bronchodilator drugs: ☐0 No ☐1 Yes

8a. If **Yes**, check all that apply:

- ☐ Albuterol (Nebulized or MDI) Dose: _____ ☐ mcg ☐ mg Given every __ __ hour(s)
- ☐ Levalbuterol (Nebulized or MDI) Dose: _____ ☐ mcg ☐ mg Given every __ __ hour(s)
- ☐ Ipratropium bromide (Nebulized or MDI) Dose: _____ ☐ mcg ☐ mg Given every __ __ hour(s)
- ☐ Formoterol Dose: _____ mcg Given every __ __ hour(s)
- ☐ Racemic epinephrine (Nebulized) Dose: _____ mg Given every __ __ hour(s)
- ☐ Other (*record on the Additional Medication Log*)

NOTE: If utilizing a combination therapy drug, record the individual components in their respective drug categories. For example Aldactazide (Spironolactone and Hydrochlorothiazide) would be recorded as Spironolactone and Hydrochlorothiazide.

9. Diuretic drugs: ☐0 No ☐1 Yes

9a. If **Yes**, check all that apply:

☐ Furosemide Dose: _____ mg Given every __ __ hour(s) Route: ☐1 PO (oral) ☐2 IV (intravenous)

☐ Bumetanide Dose: _____ mg Given every __ __ hour(s) Route: ☐1 PO (oral) ☐2 IV (intravenous)

☐ Chlorothiazide Dose: _____ mg Given every __ __ hour(s) Route: ☐1 PO (oral) ☐2 IV (intravenous)

☐ Hydrochlorothiazide Dose: _____ mg Given every __ __ hour(s)

☐ Spironolactone Dose: _____ mg Given every __ __ hour(s)

☐ Metolazone Dose: _____ mg Given every __ __ hour(s)

☐ Other (*record on the Additional Medication Log*)

10. Cardiovascular drugs: ☐0 No ☐1 Yes

10a. If **Yes**, check all that apply:

☐ Epinephrine Infusion
☐ Dopamine Infusion
☐ Dobutamine Infusion
☐ Vasopressin
☐ Norepinephrine

11. Other Cardio/Respiratory drugs: ☐0 No ☐1 Yes

11a. If **Yes**, check all that apply:

☐ Vitamin A IM or Oral (for prevention of BPD)
☐ Surfactant
☐ Sildenafil
☐ Milrinone
☐ Other pulmonary vasodilators for treatment of pulmonary hypertension other than Nitric Oxide
☐ Mucolytic
☐ Dornase alfa

12. Neuro-Muscular Blocking Agent: ☐0 No ☐1 Yes

13. Antimicrobial drugs and other agents to prevent infections: ☐0 No ☐1 Yes

13a. If **Yes**, check all that apply:

☐ Antibacterial
☐ Antifungal

☐ Antiviral
☐ Palivizumab
☐ Probiotic

14. Anxiolytic, Anticonvulsant, and Narcotic Analgesic drugs: ☐$_0$ No ☐$_1$ Yes

14a. If **Yes**, check all that apply:
☐ Benzodiazepines
☐ Phenobarbital
☐ Phenytoin
☐ Levetiracetam
☐ Morphine
☐ Fentanyl
☐ Methadone
☐ Dexmedetomidine

15. Anti-Gastroesophageal Reflux drugs: ☐$_0$ No ☐$_1$ Yes

15a. If **Yes**, check all that apply:
☐ Proton Pump Inhibitors
☐ H_2 Receptor Antagonists
☐ Motility agents
☐ Other

16. Use of blood products and Hematologic Supplements: ☐$_0$ No ☐$_1$ Yes

16a. If **Yes**, check all that apply:
☐ Erythropoietin
☐ Red Blood Cell Transfusion
☐ Platelets
☐ Iron Supplements

17. Oral Vitamins and Electrolyte Supplements: ☐$_0$ No ☐$_1$ Yes

17a. If **Yes**, check all that apply:
☐ Vitamin D
☐ Vitamin E
☐ Multivitamin
☐ Potassium Supplement
☐ Sodium Supplement

DAILY RESPIRATORY DATA DATE:__ __ / __ __ / __ __ __ __

WEEK __ __ | *DAY* 0 1

1. Did the baby receive any supplemental oxygen today? ☐0 No ☐1 Yes

   1a. If **Yes**, how long was supplemental oxygen used for? ☐1 12 hours or less ☐2 More than 12 hours

   1b. What was the concentration of supplemental oxygen at 1200 (Noon) today? __ __ __ %

2. Did the baby receive any other respiratory support today? ☐0 No ☐1 Yes

   If Questions 1 or 2 are **Yes**, answer questions 3 – 6.

3. Was Positive Airway Pressure with Endotracheal Tube used today? ☐0 No ☐1 Yes

   3a. If **Yes**, select ventilation mode and record the associated values at 1200 (Noon) or closest recorded data to 12 (Noon) if noon data are not available.

   ☐ Conventional Mechanical Ventilation (CMV):
   Mean Airway Pressure (MAP): __ __ . __ cmH_2O
   Positive End Expiratory Pressure (PEEP): __ __ cmH_2O

   ☐ High Frequency Oscillation:
   MAP: __ __ . __ cmH_2O

   ☐ High Frequency Jet Ventilation:
   MAP: __ __ . _ cmH_2O

4. Was Respiratory support without Endotracheal Tube used today? ☐0 No ☐1 Yes

   4a. If **Yes**, select ventilation mode and record the associated values at 1200 (Noon) or closest recorded data to 12 (Noon) if noon data are not available.

   ☐ Nasal Intermittent Mandatory Ventilation (NIMV):
   MAP: __ __ . __cmH_2O
   PEEP: __ __ cmH_2O

   ☐ Continuous Positive Airway Pressure (CPAP):
   CPAP: __ __ cmH_2O

   ☐ Nasal Cannula with flow rate:
   Nasal Cannula Flow: __ __ . __ __ __ __ Lpm

5. Was inhaled nitric oxide given today? ☐0 No ☐1 Yes

   5a. If **Yes**, record the concentration at 1200 (Noon) or closest recorded data to 12 (Noon) if noon data are not available. __ __ ppm

6. Was the baby reintubated today? ☐0 No ☐1 Yes

   6a. If **Yes**, indicate the primary reason why the baby was reintubated:
   ☐1 Increasing respiratory distress
   ☐2 Stridor
   ☐3 Apnea and Bradycardia
   ☐4 Suspected infection
   ☐5 For diagnostic or therapeutic procedures, including surgery
   ☐6 Unplanned extubation(s), indicate the number of occurrences this day: __
   ☐98 Other, specify: ________________________

Daily Growth and Nutrition Data

For question 1 and 2: If more than one measurement is taken on the same day, enter the first value obtained.

1. What was the baby's body weight? __ __ __ __ gms ☐99 Not done
2. What was the baby's head circumference? __ __ . __cm ☐99 Not done
3. How much milk did the baby receive today?
   - ☐0 None
   - ☐1 Partial Feed
   - ☐2 Full Feed (no parenteral nutrition on this day)

   3a. If **Partial** or **Full** milk feed, indicate the type of milk provided:
   - ☐1 Human milk
   - ☐2 Formula
   - ☐3 Both

Daily Medication Data

4. Were any drugs given today? ☐0 No ☐1 Yes

   If **Yes**, indicate which of the following drugs were given today:

5. Methylxanthine drugs: ☐0 No ☐1 Yes

   5a. If **Yes**, choose one:
   - ☐1 Caffeine Citrate* Dose: _____ mg Given every __ __ hour(s) Route: ☐1 PO (oral) ☐2 IV (intravenous)
   - ☐2 Aminophylline Dose: _____ mg Given every __ __ hour(s)
   - ☐3 Theophylline Dose: _____ mg Given every __ __ hour(s)
   - ☐98 Other (*record on the Additional Medication Log*)

   * If your site calculates the dose of Anhydrous Caffeine Base rather than Caffeine Citrate, multiply the Caffeine Base dose by 2.

6. Systemic Corticosteroid drugs: ☐0 No ☐1 Yes

6a. If **Yes**, check all that apply:
- ☐ Hydrocortisone Dose: _____ mg Given every __ __ hour(s) Route: ☐1 PO (oral) ☐2 IV (intravenous)
- ☐ Dexamethasone Dose: _____ mg Given every __ __ hour(s) Route: ☐1 PO (oral) ☐2 IV (intravenous)
- ☐ Prednisone / Prednisolone Dose: _____ mg Given every __ __ hour(s)
- ☐ Methylprednisolone Dose: _____ mg Given every __ __ hour(s)
- ☐ Other (*record on the Additional Medication Log*)

> **NOTE**: If utilizing a combination therapy drug, record the individual components in their respective drug categories. For example Symbicort (budesonide and formoterol) would be recorded as budesonide – Inhaled Steroid and formoterol – Inhaled Bronchodilator.

7. Inhaled Steroid drugs: ☐0 No ☐1 Yes

7a. If **Yes**, check all that apply:

☐ Budesonide (Nebulized or MDI)	Dose: _____ ☐ mcg ☐ mg	Given every __ __ hour(s)
☐ Beclomethasone (MDI)	Dose: _____ mcg	Given every __ __ hour(s)
☐ Ciclesonide (MDI)	Dose: _____ mcg	Given every __ __ hour(s)
☐ Flunisolide (MDI)	Dose: _____ mcg	Given every __ __ hour(s)
☐ Fluticasone (MDI)	Dose: _____ mcg	Given every __ __ hour(s)
☐ Mometasone (MDI)	Dose: _____ mcg	Given every __ __ hour(s)
☐ Triamcinolone (MDI)	Dose: _____ mcg	Given every __ __ hour(s)
☐ Dexamethasone (Nebulized)	Dose: _____ mg	Given every __ __ hour(s)
☐ Other (*record on the Additional Medication Log*)		

> If route of administration is Metered Dose Inhalator (MDI), calculate dose by multiplying the number of puffs by strength per puff.

8. Inhaled Bronchodilator drugs: ☐0 No ☐1 Yes

8a. If **Yes**, check all that apply:

☐ Albuterol (Nebulized or MDI)	Dose: _____ ☐ mcg ☐ mg	Given every __ __ hour(s)
☐ Levalbuterol (Nebulized or MDI)	Dose: _____ ☐ mcg ☐ mg	Given every __ __ hour(s)
☐ Ipratropium bromide (Nebulized or MDI)	Dose: _____ ☐ mcg ☐ mg	Given every __ __ hour(s)
☐ Formoterol	Dose: _____ mcg	Given every __ __ hour(s)
☐ Racemic epinephrine (Nebulized)	Dose: _____ mg	Given every __ __ hour(s)
☐ Other (*record on the Additional Medication Log*)		

NOTE: If utilizing a combination therapy drug, record the individual components in their respective drug categories. For example Aldactazide (Spironolactone and Hydrochlorothiazide) would be recorded as Spironolactone and Hydrochlorothiazide.

9. Diuretic drugs: ☐0 No ☐1 Yes

9a. If **Yes**, check all that apply:

☐ Furosemide Dose: _____ mg Given every __ __ hour(s) Route: ☐1 PO (oral) ☐2 IV (intravenous)

☐ Bumetanide Dose: _____ mg Given every __ __ hour(s) Route: ☐1 PO (oral) ☐2 IV (intravenous)

☐ Chlorothiazide Dose: _____ mg Given every __ __ hour(s) Route: ☐1 PO (oral) ☐2 IV (intravenous)

☐ Hydrochlorothiazide Dose: _____ mg Given every __ __ hour(s)

☐ Spironolactone Dose: _____ mg Given every __ __ hour(s)

☐ Metolazone Dose: _____ mg Given every __ __ hour(s)

☐ Other (*record on the Additional Medication Log*)

10. Cardiovascular drugs: ☐0 No ☐1 Yes

10a. If **Yes**, check all that apply:

☐ Epinephrine Infusion ☐ Vasopressin

☐ Dopamine Infusion ☐ Norepinephrine

☐ Dobutamine Infusion

11. Other Cardio/Respiratory drugs: ☐0 No ☐1 Yes

11a. If **Yes**, check all that apply:

☐ Vitamin A IM or Oral (for prevention of BPD)

☐ Other pulmonary vasodilators for treatment of pulmonary hypertension other than Nitric Oxide

☐ Surfactant ☐ Mucolytic

☐ Sildenafil ☐ Dornase alfa

☐ Milrinone

12. Neuro-Muscular Blocking Agent: ☐0 No ☐1 Yes

13. Antimicrobial drugs and other agents to prevent infections: ☐0 No ☐1 Yes

13a. If **Yes**, check all that apply:

☐ Antibacterial

☐ Antifungal

☐ Antiviral
☐ Palivizumab
☐ Probiotic

14. Anxiolytic, Anticonvulsant, and Narcotic Analgesic drugs: ☐0 No ☐1 Yes

14a. If **Yes**, check all that apply:
☐ Benzodiazepines
☐ Phenobarbital
☐ Phenytoin
☐ Levetiracetam
☐ Morphine
☐ Fentanyl
☐ Methadone
☐ Dexmedetomidine

15. Anti-Gastroesophageal Reflux drugs: ☐0 No ☐1 Yes

15a. If **Yes**, check all that apply:
☐ Proton Pump Inhibitors
☐ H_2 Receptor Antagonists
☐ Motility agents
☐ Other

16. Use of blood products and Hematologic Supplements: ☐0 No ☐1 Yes

16a. If **Yes**, check all that apply:
☐ Erythropoietin
☐ Red Blood Cell Transfusion
☐ Platelets
☐ Iron Supplements

17. Oral Vitamins and Electrolyte Supplements: ☐0 No ☐1 Yes

17a. If **Yes**, check all that apply:
☐ Vitamin D
☐ Vitamin E
☐ Multivitamin
☐ Potassium Supplement
☐ Sodium Supplement

PREMATURITY AND RESPIRATORY OUTCOMES PROGRAM

PID:__ __ __ __ __ __ __

DAILY RESPIRATORY DATA

DATE:__ __ / __ __ / __ __ __ __

WEEK __ __ | DAY 0 2

1. Did the baby receive any supplemental oxygen today? ☐0 No ☐1 Yes

   1a. If **Yes**, how long was supplemental oxygen used for?
   ☐1 12 hours or less
   ☐2 More than 12 hours

   1b. What was the concentration of supplemental oxygen at 1200 (Noon) today? __ __ __ %

2. Did the baby receive any other respiratory support today? ☐0 No ☐1 Yes

   If Questions 1 or 2 are **Yes**, answer questions 3 – 6.

3. Was Positive Airway Pressure with Endotracheal Tube used today? ☐0 No ☐1 Yes

   3a. If **Yes**, select ventilation mode and record the associated values at 1200 (Noon) or closest recorded data to 12 (Noon) if noon data are not available.

   ☐ Conventional Mechanical Ventilation (CMV):
   Mean Airway Pressure (MAP): __ __ . __ cmH_2O
   Positive End Expiratory Pressure (PEEP): __ __ cmH_2O

   ☐ High Frequency Oscillation:
   MAP: __ __ . __ cmH_2O

   ☐ High Frequency Jet Ventilation:
   MAP: __ __ . _ cmH_2O

4. Was Respiratory support without Endotracheal Tube used today? ☐0 No ☐1 Yes

   4a. If **Yes**, select ventilation mode and record the associated values at 1200 (Noon) or closest recorded data to 12 (Noon) if noon data are not available.

   ☐ Nasal Intermittent Mandatory Ventilation (NIMV):
   MAP: __ __ . __cmH_2O
   PEEP: __ __ cmH_2O

   ☐ Continuous Positive Airway Pressure (CPAP):
   CPAP: __ __ cmH_2O

   ☐ Nasal Cannula with flow rate:
   Nasal Cannula Flow: __ __ . __ __ __ __ Lpm

5. Was inhaled nitric oxide given today? ☐0 No ☐1 Yes

   5a. If **Yes**, record the concentration at 1200 (Noon) or closest recorded data to 12 (Noon) if noon data are not available. __ __ ppm

6. Was the baby reintubated today? ☐0 No ☐1 Yes

   6a. If **Yes**, indicate the primary reason why the baby was reintubated:
   ☐1 Increasing respiratory distress
   ☐2 Stridor
   ☐3 Apnea and Bradycardia
   ☐4 Suspected infection
   ☐5 For diagnostic or therapeutic procedures, including surgery
   ☐6 Unplanned extubation(s), indicate the number of occurrences this day: __
   ☐98 Other, specify: ________________________

PREMATURITY AND RESPIRATORY OUTCOMES PROGRAM

PID:__ __ __ __ __ __ __

DAILY GROWTH AND NUTRITION / DAILY MEDICATION DATA

DATE:__ __ / __ __ / __ __ __ __

WEEK __ __ | DAY 0 3

Daily Growth and Nutrition Data

For question 1 and 2: If more than one measurement is taken on the same day, enter the first value obtained.

1. What was the baby's body weight? __ __ __ __ gms ☐99 Not done
2. What was the baby's head circumference? __ __ . __cm ☐99 Not done
3. How much milk did the baby receive today?
   - ☐0 None
   - ☐1 Partial Feed
   - ☐2 Full Feed (no parenteral nutrition on this day)

   3a. If **Partial** or **Full** milk feed, indicate the type of milk provided:
   - ☐1 Human milk
   - ☐2 Formula
   - ☐3 Both

Daily Medication Data

4. Were any drugs given today? ☐0 No ☐1 Yes

   If **Yes**, indicate which of the following drugs were given today:

5. Methylxanthine drugs: ☐0 No ☐1 Yes

   5a. If **Yes**, choose one:
   - ☐1 Caffeine Citrate* Dose: _____ mg Given every __ __ hour(s) Route: ☐1 PO (oral) ☐2 IV (intravenous)
   - ☐2 Aminophylline Dose: _____ mg Given every __ __ hour(s)
   - ☐3 Theophylline Dose: _____ mg Given every __ __ hour(s)
   - ☐98 Other (*record on the Additional Medication Log*)

   * If your site calculates the dose of Anhydrous Caffeine Base rather than Caffeine Citrate, multiply the Caffeine Base dose by 2.

6. Systemic Corticosteroid drugs: ☐0 No ☐1 Yes

6a. If **Yes**, check all that apply:
- ☐ Hydrocortisone Dose: _____ mg Given every __ __ hour(s) Route: ☐1 PO (oral) ☐2 IV (intravenous)
- ☐ Dexamethasone Dose: _____ mg Given every __ __ hour(s) Route: ☐1 PO (oral) ☐2 IV (intravenous)
- ☐ Prednisone / Prednisolone Dose: _____ mg Given every __ __ hour(s)
- ☐ Methylprednisolone Dose: _____ mg Given every __ __ hour(s)
- ☐ Other (*record on the Additional Medication Log*)

NOTE: If utilizing a combination therapy drug, record the individual components in their respective drug categories. For example Symbicort (budesonide and formoterol) would be recorded as budesonide – Inhaled Steroid and formoterol – Inhaled Bronchodilator.

7. Inhaled Steroid drugs: ☐0 No ☐1 Yes

7a. If **Yes**, check all that apply:

- ☐ Budesonide (Nebulized or MDI) Dose: _____ ☐ mcg ☐ mg Given every __ __ hour(s)
- ☐ Beclomethasone (MDI) Dose: _____ mcg Given every __ __ hour(s)
- ☐ Ciclesonide (MDI) Dose: _____ mcg Given every __ __ hour(s)
- ☐ Flunisolide (MDI) Dose: _____ mcg Given every __ __ hour(s)
- ☐ Fluticasone (MDI) Dose: _____ mcg Given every __ __ hour(s)
- ☐ Mometasone (MDI) Dose: _____ mcg Given every __ __ hour(s)
- ☐ Triamcinolone (MDI) Dose: _____ mcg Given every __ __ hour(s)
- ☐ Dexamethasone (Nebulized) Dose: _____ mg Given every __ __ hour(s)
- ☐ Other (*record on the Additional Medication Log*)

If route of administration is Metered Dose Inhalator (MDI), calculate dose by multiplying the number of puffs by strength per puff.

8. Inhaled Bronchodilator drugs: ☐0 No ☐1 Yes

8a. If **Yes**, check all that apply:

- ☐ Albuterol (Nebulized or MDI) Dose: _____ ☐ mcg ☐ mg Given every __ __ hour(s)
- ☐ Levalbuterol (Nebulized or MDI) Dose: _____ ☐ mcg ☐ mg Given every __ __ hour(s)
- ☐ Ipratropium bromide (Nebulized or MDI) Dose: _____ ☐ mcg ☐ mg Given every __ __ hour(s)
- ☐ Formoterol Dose: _____ mcg Given every __ __ hour(s)
- ☐ Racemic epinephrine (Nebulized) Dose: _____ mg Given every __ __ hour(s)
- ☐ Other (*record on the Additional Medication Log*)

NOTE: If utilizing a combination therapy drug, record the individual components in their respective drug categories. For example Aldactazide (Spironolactone and Hydrochlorothiazide) would be recorded as Spironolactone and Hydrochlorothiazide.

9. Diuretic drugs: ☐0 No ☐1 Yes

9a. If **Yes**, check all that apply:

☐ Furosemide Dose: _____ mg Given every __ __ hour(s) Route: ☐1 PO (oral) ☐2 IV (intravenous)

☐ Bumetanide Dose: _____ mg Given every __ __ hour(s) Route: ☐1 PO (oral) ☐2 IV (intravenous)

☐ Chlorothiazide Dose: _____ mg Given every __ __ hour(s) Route: ☐1 PO (oral) ☐2 IV (intravenous)

☐ Hydrochlorothiazide Dose: _____ mg Given every __ __ hour(s)

☐ Spironolactone Dose: _____ mg Given every __ __ hour(s)

☐ Metolazone Dose: _____ mg Given every __ __ hour(s)

☐ Other (*record on the Additional Medication Log*)

10. Cardiovascular drugs: ☐0 No ☐1 Yes

10a. If **Yes**, check all that apply:

☐ Epinephrine Infusion ☐ Vasopressin

☐ Dopamine Infusion ☐ Norepinephrine

☐ Dobutamine Infusion

11. Other Cardio/Respiratory drugs: ☐0 No ☐1 Yes

11a. If **Yes**, check all that apply:

☐ Vitamin A IM or Oral (for prevention of BPD)

☐ Other pulmonary vasodilators for treatment of pulmonary hypertension other than Nitric Oxide

☐ Surfactant ☐ Mucolytic

☐ Sildenafil ☐ Dornase alfa

☐ Milrinone

12. Neuro-Muscular Blocking Agent: ☐0 No ☐1 Yes

13. Antimicrobial drugs and other agents to prevent infections: ☐0 No ☐1 Yes

13a. If **Yes**, check all that apply:

☐ Antibacterial

☐ Antifungal

☐ Antiviral
☐ Palivizumab
☐ Probiotic

14. Anxiolytic, Anticonvulsant, and Narcotic Analgesic drugs: ☐$_0$ No ☐$_1$ Yes

14a. If **Yes**, check all that apply:
☐ Benzodiazepines
☐ Phenobarbital
☐ Phenytoin
☐ Levetiracetam
☐ Morphine
☐ Fentanyl
☐ Methadone
☐ Dexmedetomidine

15. Anti-Gastroesophageal Reflux drugs: ☐$_0$ No ☐$_1$ Yes

15a. If **Yes**, check all that apply:
☐ Proton Pump Inhibitors
☐ H_2 Receptor Antagonists
☐ Motility agents
☐ Other

16. Use of blood products and Hematologic Supplements: ☐$_0$ No ☐$_1$ Yes

16a. If **Yes**, check all that apply:
☐ Erythropoietin
☐ Red Blood Cell Transfusion
☐ Platelets
☐ Iron Supplements

17. Oral Vitamins and Electrolyte Supplements: ☐$_0$ No ☐$_1$ Yes

17a. If **Yes**, check all that apply:
☐ Vitamin D
☐ Vitamin E
☐ Multivitamin
☐ Potassium Supplement
☐ Sodium Supplement

PREMATURITY AND RESPIRATORY OUTCOMES PROGRAM PID:__ __ __ __ __ __ __

DAILY RESPIRATORY DATA DATE:__ __ / __ __ / __ __ __ __

WEEK __ __ | *DAY* 0 3

1. Did the baby receive any supplemental oxygen today? ☐0 No ☐1 Yes

   1a. If **Yes**, how long was supplemental oxygen used for?
   ☐1 12 hours or less
   ☐2 More than 12 hours

   1b. What was the concentration of supplemental oxygen at 1200 (Noon) today? __ __ __ %

2. Did the baby receive any other respiratory support today? ☐0 No ☐1 Yes

   If Questions 1 or 2 are **Yes**, answer questions 3 – 6.

3. Was Positive Airway Pressure with Endotracheal Tube used today? ☐0 No ☐1 Yes

   3a. If **Yes**, select ventilation mode and record the associated values at 1200 (Noon) or closest recorded data to 12 (Noon) if noon data are not available.

   ☐ Conventional Mechanical Ventilation (CMV):
   Mean Airway Pressure (MAP): __ __ . __ cmH_2O
   Positive End Expiratory Pressure (PEEP): __ __ cmH_2O

   ☐ High Frequency Oscillation:
   MAP: __ __ . __ cmH_2O

   ☐ High Frequency Jet Ventilation:
   MAP: __ __ . _ cmH_2O

4. Was Respiratory support without Endotracheal Tube used today? ☐0 No ☐1 Yes

   4a. If **Yes**, select ventilation mode and record the associated values at 1200 (Noon) or closest recorded data to 12 (Noon) if noon data are not available.

   ☐ Nasal Intermittent Mandatory Ventilation (NIMV):
   MAP: __ __ . __cmH_2O
   PEEP: __ __ cmH_2O

   ☐ Continuous Positive Airway Pressure (CPAP):
   CPAP: __ __ cmH_2O

   ☐ Nasal Cannula with flow rate:
   Nasal Cannula Flow: __ __ . __ __ __ __ Lpm

5. Was inhaled nitric oxide given today? ☐0 No ☐1 Yes

   5a. If **Yes**, record the concentration at 1200 (Noon) or closest recorded data to 12 (Noon) if noon data are not available. __ __ ppm

6. Was the baby reintubated today? ☐0 No ☐1 Yes

   6a. If **Yes**, indicate the primary reason why the baby was reintubated:
   ☐1 Increasing respiratory distress
   ☐2 Stridor
   ☐3 Apnea and Bradycardia
   ☐4 Suspected infection
   ☐5 For diagnostic or therapeutic procedures, including surgery
   ☐6 Unplanned extubation(s), indicate the number of occurrences this day: __
   ☐98 Other, specify: ________________________

Daily Growth and Nutrition Data

For question 1 and 2: If more than one measurement is taken on the same day, enter the first value obtained.

1. What was the baby's body weight? __ __ __ __ gms ☐99 Not done
2. What was the baby's head circumference? __ __ . __cm ☐99 Not done
3. How much milk did the baby receive today?
   - ☐0 None
   - ☐1 Partial Feed
   - ☐2 Full Feed (no parenteral nutrition on this day)

   3a. If **Partial** or **Full** milk feed, indicate the type of milk provided:
   - ☐1 Human milk
   - ☐2 Formula
   - ☐3 Both

Daily Medication Data

4. Were any drugs given today? ☐0 No ☐1 Yes

   If **Yes**, indicate which of the following drugs were given today:

5. Methylxanthine drugs: ☐0 No ☐1 Yes

   5a. If **Yes**, choose one:

   ☐1 Caffeine Citrate* Dose: _____ mg Given every __ __ hour(s) Route: ☐1 PO (oral) ☐2 IV (intravenous)

   ☐2 Aminophylline Dose: _____ mg Given every __ __ hour(s)

   ☐3 Theophylline Dose: _____ mg Given every __ __ hour(s)

   ☐98 Other (*record on the Additional Medication Log*)

   * If your site calculates the dose of Anhydrous Caffeine Base rather than Caffeine Citrate, multiply the Caffeine Base dose by 2.

6. Systemic Corticosteroid drugs: ☐0 No ☐1 Yes

6a. If **Yes**, check all that apply:

☐ Hydrocortisone Dose: _____ mg Given every __ __ hour(s) Route: ☐1 PO (oral) ☐2 IV (intravenous)

☐ Dexamethasone Dose: _____ mg Given every __ __ hour(s) Route: ☐1 PO (oral) ☐2 IV (intravenous)

☐ Prednisone / Prednisolone Dose: _____ mg Given every __ __ hour(s)

☐ Methylprednisolone Dose: _____ mg Given every __ __ hour(s)

☐ Other (*record on the Additional Medication Log*)

NOTE: If utilizing a combination therapy drug, record the individual components in their respective drug categories. For example Symbicort (budesonide and formoterol) would be recorded as budesonide – Inhaled Steroid and formoterol – Inhaled Bronchodilator.

7. Inhaled Steroid drugs: ☐ $_0$ No ☐ $_1$ Yes

7a. If **Yes**, check all that apply:

- ☐ Budesonide (Nebulized or MDI) Dose: _____ ☐ mcg ☐ mg Given every __ __ hour(s)
- ☐ Beclomethasone (MDI) Dose: _____ mcg Given every __ __ hour(s)
- ☐ Ciclesonide (MDI) Dose: _____ mcg Given every __ __ hour(s)
- ☐ Flunisolide (MDI) Dose: _____ mcg Given every __ __ hour(s)
- ☐ Fluticasone (MDI) Dose: _____ mcg Given every __ __ hour(s)
- ☐ Mometasone (MDI) Dose: _____ mcg Given every __ __ hour(s)
- ☐ Triamcinolone (MDI) Dose: _____ mcg Given every __ __ hour(s)
- ☐ Dexamethasone (Nebulized) Dose: _____ mg Given every __ __ hour(s)
- ☐ Other (*record on the Additional Medication Log*)

If route of administration is Metered Dose Inhalator (MDI), calculate dose by multiplying the number of puffs by strength per puff.

8. Inhaled Bronchodilator drugs: ☐ $_0$ No ☐ $_1$ Yes

8a. If **Yes**, check all that apply:

- ☐ Albuterol (Nebulized or MDI) Dose: _____ ☐ mcg ☐ mg Given every __ __ hour(s)
- ☐ Levalbuterol (Nebulized or MDI) Dose: _____ ☐ mcg ☐ mg Given every __ __ hour(s)
- ☐ Ipratropium bromide (Nebulized or MDI) Dose: _____ ☐ mcg ☐ mg Given every __ __ hour(s)
- ☐ Formoterol Dose: _____ mcg Given every __ __ hour(s)
- ☐ Racemic epinephrine (Nebulized) Dose: _____ mg Given every __ __ hour(s)
- ☐ Other (*record on the Additional Medication Log*)

NOTE: If utilizing a combination therapy drug, record the individual components in their respective drug categories. For example Aldactazide (Spironolactone and Hydrochlorothiazide) would be recorded as Spironolactone and Hydrochlorothiazide.

9. Diuretic drugs: ☐0 No ☐1 Yes

9a. If **Yes**, check all that apply:

☐ Furosemide Dose: _____ mg Given every __ __ hour(s) Route: ☐1 PO (oral) ☐2 IV (intravenous)

☐ Bumetanide Dose: _____ mg Given every __ __ hour(s) Route: ☐1 PO (oral) ☐2 IV (intravenous)

☐ Chlorothiazide Dose: _____ mg Given every __ __ hour(s) Route: ☐1 PO (oral) ☐2 IV (intravenous)

☐ Hydrochlorothiazide Dose: _____ mg Given every __ __ hour(s)

☐ Spironolactone Dose: _____ mg Given every __ __ hour(s)

☐ Metolazone Dose: _____ mg Given every __ __ hour(s)

☐ Other (*record on the Additional Medication Log*)

10. Cardiovascular drugs: ☐0 No ☐1 Yes

10a. If **Yes**, check all that apply:

☐ Epinephrine Infusion ☐ Vasopressin

☐ Dopamine Infusion ☐ Norepinephrine

☐ Dobutamine Infusion

11. Other Cardio/Respiratory drugs: ☐0 No ☐1 Yes

11a. If **Yes**, check all that apply:

☐ Vitamin A IM or Oral (for prevention of BPD)

☐ Other pulmonary vasodilators for treatment of pulmonary hypertension other than Nitric Oxide

☐ Surfactant ☐ Mucolytic

☐ Sildenafil ☐ Dornase alfa

☐ Milrinone

12. Neuro-Muscular Blocking Agent: ☐0 No ☐1 Yes

13. Antimicrobial drugs and other agents to prevent infections: ☐0 No ☐1 Yes

13a. If **Yes**, check all that apply:

☐ Antibacterial

☐ Antifungal

☐ Antiviral
☐ Palivizumab
☐ Probiotic

14. Anxiolytic, Anticonvulsant, and Narcotic Analgesic drugs: ☐0 No ☐1 Yes

14a. If **Yes**, check all that apply:
☐ Benzodiazepines
☐ Phenobarbital
☐ Phenytoin
☐ Levetiracetam
☐ Morphine
☐ Fentanyl
☐ Methadone
☐ Dexmedetomidine

15. Anti-Gastroesophageal Reflux drugs: ☐0 No ☐1 Yes

15a. If **Yes**, check all that apply:
☐ Proton Pump Inhibitors
☐ H_2 Receptor Antagonists
☐ Motility agents
☐ Other

16. Use of blood products and Hematologic Supplements: ☐0 No ☐1 Yes

16a. If **Yes**, check all that apply:
☐ Erythropoietin
☐ Red Blood Cell Transfusion
☐ Platelets
☐ Iron Supplements

17. Oral Vitamins and Electrolyte Supplements: ☐0 No ☐1 Yes

17a. If **Yes**, check all that apply:
☐ Vitamin D
☐ Vitamin E
☐ Multivitamin
☐ Potassium Supplement
☐ Sodium Supplement

1. Did the baby receive any supplemental oxygen today? ☐0 No ☐1 Yes

   1a. If **Yes**, how long was supplemental oxygen used for? ☐1 12 hours or less ☐2 More than 12 hours

   1b. What was the concentration of supplemental oxygen at 1200 (Noon) today? __ __ __ %

2. Did the baby receive any other respiratory support today? ☐0 No ☐1 Yes

   If Questions 1 or 2 are **Yes**, answer questions 3 – 6.

3. Was Positive Airway Pressure with Endotracheal Tube used today? ☐0 No ☐1 Yes
   3a. If **Yes**, select ventilation mode and record the associated values at 1200 (Noon) or closest recorded data to 12 (Noon) if noon data are not available.

   ☐ Conventional Mechanical Ventilation (CMV):
   Mean Airway Pressure (MAP): __ __ . __ cmH_2O
   Positive End Expiratory Pressure (PEEP): __ __ cmH_2O

   ☐ High Frequency Oscillation:
   MAP: __ __ . __ cmH_2O

   ☐ High Frequency Jet Ventilation:
   MAP: __ __ . _ cmH_2O

4. Was Respiratory support without Endotracheal Tube used today? ☐0 No ☐1 Yes
   4a. If **Yes**, select ventilation mode and record the associated values at 1200 (Noon) or closest recorded data to 12 (Noon) if noon data are not available.

   ☐ Nasal Intermittent Mandatory Ventilation (NIMV):
   MAP: __ __ . __cmH_2O
   PEEP: __ __ cmH_2O

   ☐ Continuous Positive Airway Pressure (CPAP):
   CPAP: __ __ cmH_2O

   ☐ Nasal Cannula with flow rate:
   Nasal Cannula Flow: __ __ . __ __ __ __ Lpm

5. Was inhaled nitric oxide given today? ☐0 No ☐1 Yes
   5a. If **Yes**, record the concentration at 1200 (Noon) or closest recorded data to 12 (Noon) if noon data are not available. __ __ ppm

6. Was the baby reintubated today? ☐0 No ☐1 Yes

   6a. If **Yes**, indicate the primary reason why the baby was reintubated:
   ☐1 Increasing respiratory distress
   ☐2 Stridor
   ☐3 Apnea and Bradycardia
   ☐4 Suspected infection
   ☐5 For diagnostic or therapeutic procedures, including surgery
   ☐6 Unplanned extubation(s), indicate the number of occurrences this day: __
   ☐98 Other, specify: ________________________

Daily Growth and Nutrition Data

For question 1 and 2: If more than one measurement is taken on the same day, enter the first value obtained.

1. What was the baby's body weight? __ __ __ __ gms ☐99 Not done
2. What was the baby's head circumference? __ __ . __cm ☐99 Not done
3. How much milk did the baby receive today?
   - ☐0 None
   - ☐1 Partial Feed
   - ☐2 Full Feed (no parenteral nutrition on this day)

   3a. If **Partial** or **Full** milk feed, indicate the type of milk provided:
   - ☐1 Human milk
   - ☐2 Formula
   - ☐3 Both

Daily Medication Data

4. Were any drugs given today? ☐0 No ☐1 Yes

   If **Yes**, indicate which of the following drugs were given today:

5. Methylxanthine drugs: ☐0 No ☐1 Yes

   5a. If **Yes**, choose one:
   - ☐1 Caffeine Citrate* Dose: _____ mg Given every __ __ hour(s) Route: ☐1 PO (oral) ☐2 IV (intravenous)
   - ☐2 Aminophylline Dose: _____ mg Given every __ __ hour(s)
   - ☐3 Theophylline Dose: _____ mg Given every __ __ hour(s)
   - ☐98 Other (*record on the Additional Medication Log*)

   * If your site calculates the dose of Anhydrous Caffeine Base rather than Caffeine Citrate, multiply the Caffeine Base dose by 2.

6. Systemic Corticosteroid drugs: ☐0 No ☐1 Yes

6a. If **Yes**, check all that apply:
- ☐ Hydrocortisone Dose: _____ mg Given every __ __ hour(s) Route: ☐1 PO (oral) ☐2 IV (intravenous)
- ☐ Dexamethasone Dose: _____ mg Given every __ __ hour(s) Route: ☐1 PO (oral) ☐2 IV (intravenous)
- ☐ Prednisone / Prednisolone Dose: _____ mg Given every __ __ hour(s)
- ☐ Methylprednisolone Dose: _____ mg Given every __ __ hour(s)
- ☐ Other (*record on the Additional Medication Log*)

NOTE: If utilizing a combination therapy drug, record the individual components in their respective drug categories. For example Symbicort (budesonide and formoterol) would be recorded as budesonide – Inhaled Steroid and formoterol – Inhaled Bronchodilator.

7. Inhaled Steroid drugs: ☐$_0$ No ☐$_1$ Yes

7a. If **Yes**, check all that apply:

☐ Budesonide (Nebulized or MDI)	Dose: _____ ☐ mcg ☐ mg	Given every __ __ hour(s)
☐ Beclomethasone (MDI)	Dose: _____ mcg	Given every __ __ hour(s)
☐ Ciclesonide (MDI)	Dose: _____ mcg	Given every __ __ hour(s)
☐ Flunisolide (MDI)	Dose: _____ mcg	Given every __ __ hour(s)
☐ Fluticasone (MDI)	Dose: _____ mcg	Given every __ __ hour(s)
☐ Mometasone (MDI)	Dose: _____ mcg	Given every __ __ hour(s)
☐ Triamcinolone (MDI)	Dose: _____ mcg	Given every __ __ hour(s)
☐ Dexamethasone (Nebulized)	Dose: _____ mg	Given every __ __ hour(s)
☐ Other (*record on the Additional Medication Log*)		

If route of administration is Metered Dose Inhalator (MDI), calculate dose by multiplying the number of puffs by strength per puff.

8. Inhaled Bronchodilator drugs: ☐$_0$ No ☐$_1$ Yes

8a. If **Yes**, check all that apply:

☐ Albuterol (Nebulized or MDI)	Dose: _____ ☐ mcg ☐ mg	Given every __ __ hour(s)
☐ Levalbuterol (Nebulized or MDI)	Dose: _____ ☐ mcg ☐ mg	Given every __ __ hour(s)
☐ Ipratropium bromide (Nebulized or MDI)	Dose: _____ ☐ mcg ☐ mg	Given every __ __ hour(s)
☐ Formoterol	Dose: _____ mcg	Given every __ __ hour(s)
☐ Racemic epinephrine (Nebulized)	Dose: _____ mg	Given every __ __ hour(s)
☐ Other (*record on the Additional Medication Log*)		

> **NOTE**: If utilizing a combination therapy drug, record the individual components in their respective drug categories. For example Aldactazide (Spironolactone and Hydrochlorothiazide) would be recorded as Spironolactone and Hydrochlorothiazide.

9. Diuretic drugs: ☐0 No ☐1 Yes

9a.If **Yes**, check all that apply:

☐ Furosemide Dose: _____ mg Given every __ __ hour(s) Route: ☐1 PO (oral) ☐2 IV (intravenous)

☐ Bumetanide Dose: _____ mg Given every __ __ hour(s) Route: ☐1 PO (oral) ☐2 IV (intravenous)

☐ Chlorothiazide Dose: _____ mg Given every __ __ hour(s) Route: ☐1 PO (oral) ☐2 IV (intravenous)

☐ Hydrochlorothiazide Dose: _____ mg Given every __ __ hour(s)

☐ Spironolactone Dose: _____ mg Given every __ __ hour(s)

☐ Metolazone Dose: _____ mg Given every __ __ hour(s)

☐ Other (*record on the Additional Medication Log*)

10. Cardiovascular drugs: ☐0 No ☐1 Yes

10a. If **Yes**, check all that apply:

☐ Epinephrine Infusion ☐ Vasopressin

☐ Dopamine Infusion ☐ Norepinephrine

☐ Dobutamine Infusion

11. Other Cardio/Respiratory drugs: ☐0 No ☐1 Yes

11a. If **Yes**, check all that apply:

☐ Vitamin A IM or Oral (for prevention of BPD)

☐ Other pulmonary vasodilators for treatment of pulmonary hypertension other than Nitric Oxide

☐ Surfactant ☐ Mucolytic

☐ Sildenafil ☐ Dornase alfa

☐ Milrinone

12. Neuro-Muscular Blocking Agent: ☐0 No ☐1 Yes

13. Antimicrobial drugs and other agents to prevent infections: ☐0 No ☐1 Yes

13a. If **Yes**, check all that apply:

☐ Antibacterial

☐ Antifungal

- ☐ Antiviral
- ☐ Palivizumab
- ☐ Probiotic

14. Anxiolytic, Anticonvulsant, and Narcotic Analgesic drugs: ☐$_0$ No ☐$_1$ Yes

14a. If **Yes**, check all that apply:
- ☐ Benzodiazepines
- ☐ Phenobarbital
- ☐ Phenytoin
- ☐ Levetiracetam
- ☐ Morphine
- ☐ Fentanyl
- ☐ Methadone
- ☐ Dexmedetomidine

15. Anti-Gastroesophageal Reflux drugs: ☐$_0$ No ☐$_1$ Yes

15a. If **Yes**, check all that apply:
- ☐ Proton Pump Inhibitors
- ☐ H_2 Receptor Antagonists
- ☐ Motility agents
- ☐ Other

16. Use of blood products and Hematologic Supplements: ☐$_0$ No ☐$_1$ Yes

16a. If **Yes**, check all that apply:
- ☐ Erythropoietin
- ☐ Red Blood Cell Transfusion
- ☐ Platelets
- ☐ Iron Supplements

17. Oral Vitamins and Electrolyte Supplements: ☐$_0$ No ☐$_1$ Yes

17a. If **Yes**, check all that apply:
- ☐ Vitamin D
- ☐ Vitamin E
- ☐ Multivitamin
- ☐ Potassium Supplement
- ☐ Sodium Supplement

DAILY RESPIRATORY DATA

DATE:__ __ / __ __ / __ __ __ __

WEEK __ __ | DAY 0 5

1. Did the baby receive any supplemental oxygen today? ☐0 No ☐1 Yes

   1a. If **Yes**, how long was supplemental oxygen used for?
   ☐1 12 hours or less
   ☐2 More than 12 hours

   1b. What was the concentration of supplemental oxygen at 1200 (Noon) today? __ __ __ %

2. Did the baby receive any other respiratory support today? ☐0 No ☐1 Yes

   If Questions 1 or 2 are **Yes**, answer questions 3 – 6.

3. Was Positive Airway Pressure with Endotracheal Tube used today? ☐0 No ☐1 Yes

   3a. If **Yes**, select ventilation mode and record the associated values at 1200 (Noon) or closest recorded data to 12 (Noon) if noon data are not available.

   ☐ Conventional Mechanical Ventilation (CMV):
   Mean Airway Pressure (MAP): __ __ . __ cmH_2O
   Positive End Expiratory Pressure (PEEP): __ __ cmH_2O

   ☐ High Frequency Oscillation:
   MAP: __ __ . __ cmH_2O

   ☐ High Frequency Jet Ventilation:
   MAP: __ __ . _ cmH_2O

4. Was Respiratory support without Endotracheal Tube used today? ☐0 No ☐1 Yes

   4a. If **Yes**, select ventilation mode and record the associated values at 1200 (Noon) or closest recorded data to 12 (Noon) if noon data are not available.

   ☐ Nasal Intermittent Mandatory Ventilation (NIMV):
   MAP: __ __ . __cmH_2O
   PEEP: __ __ cmH_2O

   ☐ Continuous Positive Airway Pressure (CPAP):
   CPAP: __ __ cmH_2O

   ☐ Nasal Cannula with flow rate:
   Nasal Cannula Flow: __ __ . __ __ __ __ Lpm

5. Was inhaled nitric oxide given today? ☐0 No ☐1 Yes

   5a. If **Yes**, record the concentration at 1200 (Noon) or closest recorded data to 12 (Noon) if noon data are not available. __ __ ppm

6. Was the baby reintubated today? ☐0 No ☐1 Yes

   6a. If **Yes**, indicate the primary reason why the baby was reintubated:
   ☐1 Increasing respiratory distress
   ☐2 Stridor
   ☐3 Apnea and Bradycardia
   ☐4 Suspected infection
   ☐5 For diagnostic or therapeutic procedures, including surgery
   ☐6 Unplanned extubation(s), indicate the number of occurrences this day: __
   ☐98 Other, specify: ________________________

Daily Growth and Nutrition Data

For question 1 and 2: If more than one measurement is taken on the same day, enter the first value obtained.

1. What was the baby's body weight? __ __ __ __ gms ☐99 Not done
2. What was the baby's head circumference? __ __ . __cm ☐99 Not done
3. How much milk did the baby receive today?
   - ☐0 None
   - ☐1 Partial Feed
   - ☐2 Full Feed (no parenteral nutrition on this day)

   3a. If **Partial** or **Full** milk feed, indicate the type of milk provided:
   - ☐1 Human milk
   - ☐2 Formula
   - ☐3 Both

Daily Medication Data

4. Were any drugs given today? ☐0 No ☐1 Yes

   If **Yes**, indicate which of the following drugs were given today:

5. Methylxanthine drugs: ☐0 No ☐1 Yes

   5a. If **Yes**, choose one:
   - ☐1 Caffeine Citrate* Dose: _____ mg Given every __ __ hour(s) Route: ☐1 PO (oral) ☐2 IV (intravenous)
   - ☐2 Aminophylline Dose: _____ mg Given every __ __ hour(s)
   - ☐3 Theophylline Dose: _____ mg Given every __ __ hour(s)
   - ☐98 Other (*record on the Additional Medication Log*)

   * If your site calculates the dose of Anhydrous Caffeine Base rather than Caffeine Citrate, multiply the Caffeine Base dose by 2.

6. Systemic Corticosteroid drugs: ☐0 No ☐1 Yes

6a. If **Yes**, check all that apply:
- ☐ Hydrocortisone Dose: _____ mg Given every __ __ hour(s) Route: ☐1 PO (oral) ☐2 IV (intravenous)
- ☐ Dexamethasone Dose: _____ mg Given every __ __ hour(s) Route: ☐1 PO (oral) ☐2 IV (intravenous)
- ☐ Prednisone / Prednisolone Dose: _____ mg Given every __ __ hour(s)
- ☐ Methylprednisolone Dose: _____ mg Given every __ __ hour(s)
- ☐ Other (*record on the Additional Medication Log*)

NOTE: If utilizing a combination therapy drug, record the individual components in their respective drug categories. For example Symbicort (budesonide and formoterol) would be recorded as budesonide – Inhaled Steroid and formoterol – Inhaled Bronchodilator.

7. Inhaled Steroid drugs: ☐0 No ☐1 Yes

7a. If **Yes**, check all that apply:

☐ Budesonide (Nebulized or MDI)	Dose: _____ ☐ mcg ☐ mg	Given every __ __ hour(s)
☐ Beclomethasone (MDI)	Dose: _____ mcg	Given every __ __ hour(s)
☐ Ciclesonide (MDI)	Dose: _____ mcg	Given every __ __ hour(s)
☐ Flunisolide (MDI)	Dose: _____ mcg	Given every __ __ hour(s)
☐ Fluticasone (MDI)	Dose: _____ mcg	Given every __ __ hour(s)
☐ Mometasone (MDI)	Dose: _____ mcg	Given every __ __ hour(s)
☐ Triamcinolone (MDI)	Dose: _____ mcg	Given every __ __ hour(s)
☐ Dexamethasone (Nebulized)	Dose: _____ mg	Given every __ __ hour(s)
☐ Other (*record on the Additional Medication Log*)		

If route of administration is Metered Dose Inhalator (MDI), calculate dose by multiplying the number of puffs by strength per puff.

8. Inhaled Bronchodilator drugs: ☐0 No ☐1 Yes

8a. If **Yes**, check all that apply:

☐ Albuterol (Nebulized or MDI)	Dose: _____ ☐ mcg ☐ mg	Given every __ __ hour(s)
☐ Levalbuterol (Nebulized or MDI)	Dose: _____ ☐ mcg ☐ mg	Given every __ __ hour(s)
☐ Ipratropium bromide (Nebulized or MDI)	Dose: _____ ☐ mcg ☐ mg	Given every __ __ hour(s)
☐ Formoterol	Dose: _____ mcg	Given every __ __ hour(s)
☐ Racemic epinephrine (Nebulized)	Dose: _____ mg	Given every __ __ hour(s)
☐ Other (*record on the Additional Medication Log*)		

NOTE: If utilizing a combination therapy drug, record the individual components in their respective drug categories. For example Aldactazide (Spironolactone and Hydrochlorothiazide) would be recorded as Spironolactone and Hydrochlorothiazide.

9. Diuretic drugs: ☐0 No ☐1 Yes

9a. If **Yes**, check all that apply:

☐ Furosemide Dose: _____ mg Given every __ __ hour(s) Route: ☐1 PO (oral) ☐2 IV (intravenous)

☐ Bumetanide Dose: _____ mg Given every __ __ hour(s) Route: ☐1 PO (oral) ☐2 IV (intravenous)

☐ Chlorothiazide Dose: _____ mg Given every __ __ hour(s) Route: ☐1 PO (oral) ☐2 IV (intravenous)

☐ Hydrochlorothiazide Dose: _____ mg Given every __ __ hour(s)

☐ Spironolactone Dose: _____ mg Given every __ __ hour(s)

☐ Metolazone Dose: _____ mg Given every __ __ hour(s)

☐ Other (*record on the Additional Medication Log*)

10. Cardiovascular drugs: ☐0 No ☐1 Yes

10a. If **Yes**, check all that apply:

☐ Epinephrine Infusion ☐ Vasopressin

☐ Dopamine Infusion ☐ Norepinephrine

☐ Dobutamine Infusion

11. Other Cardio/Respiratory drugs: ☐0 No ☐1 Yes

11a. If **Yes**, check all that apply:

☐ Vitamin A IM or Oral (for prevention of BPD)

☐ Other pulmonary vasodilators for treatment of pulmonary hypertension other than Nitric Oxide

☐ Surfactant ☐ Mucolytic

☐ Sildenafil ☐ Dornase alfa

☐ Milrinone

12. Neuro-Muscular Blocking Agent: ☐0 No ☐1 Yes

13. Antimicrobial drugs and other agents to prevent infections: ☐0 No ☐1 Yes

13a. If **Yes**, check all that apply:

☐ Antibacterial

☐ Antifungal

☐ Antiviral
☐ Palivizumab
☐ Probiotic

14. Anxiolytic, Anticonvulsant, and Narcotic Analgesic drugs: ☐$_0$ No ☐$_1$ Yes

14a. If **Yes**, check all that apply:
☐ Benzodiazepines
☐ Phenobarbital
☐ Phenytoin
☐ Levetiracetam
☐ Morphine
☐ Fentanyl
☐ Methadone
☐ Dexmedetomidine

15. Anti-Gastroesophageal Reflux drugs: ☐$_0$ No ☐$_1$ Yes

15a. If **Yes**, check all that apply:
☐ Proton Pump Inhibitors
☐ H_2 Receptor Antagonists
☐ Motility agents
☐ Other

16. Use of blood products and Hematologic Supplements: ☐$_0$ No ☐$_1$ Yes

16a. If **Yes**, check all that apply:
☐ Erythropoietin
☐ Red Blood Cell Transfusion
☐ Platelets
☐ Iron Supplements

17. Oral Vitamins and Electrolyte Supplements: ☐$_0$ No ☐$_1$ Yes

17a. If **Yes**, check all that apply:
☐ Vitamin D
☐ Vitamin E
☐ Multivitamin
☐ Potassium Supplement
☐ Sodium Supplement

PREMATURITY AND RESPIRATORY OUTCOMES PROGRAM PID:__ __ __ __ __ __ __

DAILY RESPIRATORY DATA DATE:__ __ / __ __ / __ __ __ __

WEEK __ __ | *DAY* 0 6

1. Did the baby receive any supplemental oxygen today? ☐0 No ☐1 Yes

   1a. If **Yes**, how long was supplemental oxygen used for? ☐1 12 hours or less ☐2 More than 12 hours

   1b. What was the concentration of supplemental oxygen at 1200 (Noon) today? __ __ __ %

2. Did the baby receive any other respiratory support today? ☐0 No ☐1 Yes

   If Questions 1 or 2 are **Yes**, answer questions 3 – 6.

3. Was Positive Airway Pressure with Endotracheal Tube used today? ☐0 No ☐1 Yes

   3a. If **Yes**, select ventilation mode and record the associated values at 1200 (Noon) or closest recorded data to 12 (Noon) if noon data are not available.

   ☐ Conventional Mechanical Ventilation (CMV):
   Mean Airway Pressure (MAP): __ __ . __ cmH_2O
   Positive End Expiratory Pressure (PEEP): __ __ cmH_2O

   ☐ High Frequency Oscillation:
   MAP: __ __ . __ cmH_2O

   ☐ High Frequency Jet Ventilation:
   MAP: __ __ . _ cmH_2O

4. Was Respiratory support without Endotracheal Tube used today? ☐0 No ☐1 Yes

   4a. If **Yes**, select ventilation mode and record the associated values at 1200 (Noon) or closest recorded data to 12 (Noon) if noon data are not available.

   ☐ Nasal Intermittent Mandatory Ventilation (NIMV):
   MAP: __ __ . __cmH_2O
   PEEP: __ __ cmH_2O

   ☐ Continuous Positive Airway Pressure (CPAP):
   CPAP: __ __ cmH_2O

   ☐ Nasal Cannula with flow rate:
   Nasal Cannula Flow: __ __ . __ __ __ __ Lpm

5. Was inhaled nitric oxide given today? ☐0 No ☐1 Yes

   5a. If **Yes**, record the concentration at 1200 (Noon) or closest recorded data to 12 (Noon) if noon data are not available. __ __ ppm

6. Was the baby reintubated today? ☐0 No ☐1 Yes

   6a. If **Yes**, indicate the primary reason why the baby was reintubated:
   ☐1 Increasing respiratory distress
   ☐2 Stridor
   ☐3 Apnea and Bradycardia
   ☐4 Suspected infection
   ☐5 For diagnostic or therapeutic procedures, including surgery
   ☐6 Unplanned extubation(s), indicate the number of occurrences this day: __
   ☐98 Other, specify: ________________________

Daily Growth and Nutrition Data

For question 1 and 2: If more than one measurement is taken on the same day, enter the first value obtained.

1. What was the baby's body weight? __ __ __ __ gms ☐99 Not done
2. What was the baby's head circumference? __ __ . __cm ☐99 Not done
3. How much milk did the baby receive today?
   - ☐0 None
   - ☐1 Partial Feed
   - ☐2 Full Feed (no parenteral nutrition on this day)

   3a. If **Partial** or **Full** milk feed, indicate the type of milk provided:
   - ☐1 Human milk
   - ☐2 Formula
   - ☐3 Both

Daily Medication Data

4. Were any drugs given today? ☐0 No ☐1 Yes

   If **Yes**, indicate which of the following drugs were given today:

5. Methylxanthine drugs: ☐0 No ☐1 Yes

   5a. If **Yes**, choose one:
   - ☐1 Caffeine Citrate* Dose: _____ mg Given every __ __ hour(s) Route: ☐1 PO (oral) ☐2 IV (intravenous)
   - ☐2 Aminophylline Dose: _____ mg Given every __ __ hour(s)
   - ☐3 Theophylline Dose: _____ mg Given every __ __ hour(s)
   - ☐98 Other (*record on the Additional Medication Log*)

   * If your site calculates the dose of Anhydrous Caffeine Base rather than Caffeine Citrate, multiply the Caffeine Base dose by 2.

6. Systemic Corticosteroid drugs: ☐0 No ☐1 Yes

6a. If **Yes**, check all that apply:
- ☐ Hydrocortisone Dose: _____ mg Given every __ __ hour(s) Route: ☐1 PO (oral) ☐2 IV (intravenous)
- ☐ Dexamethasone Dose: _____ mg Given every __ __ hour(s) Route: ☐1 PO (oral) ☐2 IV (intravenous)
- ☐ Prednisone / Prednisolone Dose: _____ mg Given every __ __ hour(s)
- ☐ Methylprednisolone Dose: _____ mg Given every __ __ hour(s)
- ☐ Other (*record on the Additional Medication Log*)

NOTE: If utilizing a combination therapy drug, record the individual components in their respective drug categories. For example Symbicort (budesonide and formoterol) would be recorded as budesonide – Inhaled Steroid and formoterol – Inhaled Bronchodilator.

7. Inhaled Steroid drugs: ☐0 No ☐1 Yes

7a. If **Yes**, check all that apply:

- ☐ Budesonide (Nebulized or MDI) Dose: _____ ☐ mcg ☐ mg Given every __ __ hour(s)
- ☐ Beclomethasone (MDI) Dose: _____ mcg Given every __ __ hour(s)
- ☐ Ciclesonide (MDI) Dose: _____ mcg Given every __ __ hour(s)
- ☐ Flunisolide (MDI) Dose: _____ mcg Given every __ __ hour(s)
- ☐ Fluticasone (MDI) Dose: _____ mcg Given every __ __ hour(s)
- ☐ Mometasone (MDI) Dose: _____ mcg Given every __ __ hour(s)
- ☐ Triamcinolone (MDI) Dose: _____ mcg Given every __ __ hour(s)
- ☐ Dexamethasone (Nebulized) Dose: _____ mg Given every __ __ hour(s)
- ☐ Other (*record on the Additional Medication Log*)

If route of administration is Metered Dose Inhalator (MDI), calculate dose by multiplying the number of puffs by strength per puff.

8. Inhaled Bronchodilator drugs: ☐0 No ☐1 Yes

8a. If **Yes**, check all that apply:

- ☐ Albuterol (Nebulized or MDI) Dose: _____ ☐ mcg ☐ mg Given every __ __ hour(s)
- ☐ Levalbuterol (Nebulized or MDI) Dose: _____ ☐ mcg ☐ mg Given every __ __ hour(s)
- ☐ Ipratropium bromide (Nebulized or MDI) Dose: _____ ☐ mcg ☐ mg Given every __ __ hour(s)
- ☐ Formoterol Dose: _____ mcg Given every __ __ hour(s)
- ☐ Racemic epinephrine (Nebulized) Dose: _____ mg Given every __ __ hour(s)
- ☐ Other (*record on the Additional Medication Log*)

NOTE: If utilizing a combination therapy drug, record the individual components in their respective drug categories. For example Aldactazide (Spironolactone and Hydrochlorothiazide) would be recorded as Spironolactone and Hydrochlorothiazide.

9. Diuretic drugs: ☐₀ No ☐₁ Yes

9a. If **Yes**, check all that apply:

☐ Furosemide Dose: _____ mg Given every __ __ hour(s) Route: ☐1 PO (oral) ☐2 IV (intravenous)

☐ Bumetanide Dose: _____ mg Given every __ __ hour(s) Route: ☐1 PO (oral) ☐2 IV (intravenous)

☐ Chlorothiazide Dose: _____ mg Given every __ __ hour(s) Route: ☐1 PO (oral) ☐2 IV (intravenous)

☐ Hydrochlorothiazide Dose: _____ mg Given every __ __ hour(s)

☐ Spironolactone Dose: _____ mg Given every __ __ hour(s)

☐ Metolazone Dose: _____ mg Given every __ __ hour(s)

☐ Other (*record on the Additional Medication Log*)

10. Cardiovascular drugs: ☐0 No ☐1 Yes

10a. If **Yes**, check all that apply:

☐ Epinephrine Infusion ☐ Vasopressin

☐ Dopamine Infusion ☐ Norepinephrine

☐ Dobutamine Infusion

11. Other Cardio/Respiratory drugs: ☐0 No ☐1 Yes

11a. If **Yes**, check all that apply:

☐ Vitamin A IM or Oral (for prevention of BPD)

☐ Other pulmonary vasodilators for treatment of pulmonary hypertension other than Nitric Oxide

☐ Surfactant ☐ Mucolytic

☐ Sildenafil ☐ Dornase alfa

☐ Milrinone

12. Neuro-Muscular Blocking Agent: ☐0 No ☐1 Yes

13. Antimicrobial drugs and other agents to prevent infections: ☐0 No ☐1 Yes

13a. If **Yes**, check all that apply:

☐ Antibacterial

☐ Antifungal

☐ Antiviral
☐ Palivizumab
☐ Probiotic

14. Anxiolytic, Anticonvulsant, and Narcotic Analgesic drugs: ☐0 No ☐1 Yes

14a. If **Yes**, check all that apply:
☐ Benzodiazepines
☐ Phenobarbital
☐ Phenytoin
☐ Levetiracetam
☐ Morphine
☐ Fentanyl
☐ Methadone
☐ Dexmedetomidine

15. Anti-Gastroesophageal Reflux drugs: ☐0 No ☐1 Yes

15a. If **Yes**, check all that apply:
☐ Proton Pump Inhibitors
☐ H_2 Receptor Antagonists
☐ Motility agents
☐ Other

16. Use of blood products and Hematologic Supplements: ☐0 No ☐1 Yes

16a. If **Yes**, check all that apply:
☐ Erythropoietin
☐ Red Blood Cell Transfusion
☐ Platelets
☐ Iron Supplements

17. Oral Vitamins and Electrolyte Supplements: ☐0 No ☐1 Yes

17a. If **Yes**, check all that apply:
☐ Vitamin D
☐ Vitamin E
☐ Multivitamin
☐ Potassium Supplement
☐ Sodium Supplement

PREMATURITY AND RESPIRATORY OUTCOMES PROGRAM PID:__ __ __ __ __ __ __

DAILY RESPIRATORY DATA DATE:__ __ / __ __ / __ __ __ __

WEEK __ __ | *DAY* 0 7

1. Did the baby receive any supplemental oxygen today? ☐₀ No ☐₁ Yes

   1a. If **Yes**, how long was supplemental oxygen used for? ☐₁ 12 hours or less ☐₂ More than 12 hours

   1b. What was the concentration of supplemental oxygen at 1200 (Noon) today? __ __ __ %

2. Did the baby receive any other respiratory support today? ☐₀ No ☐₁ Yes

   If Questions 1 or 2 are **Yes**, answer questions 3 – 6.

3. Was Positive Airway Pressure with Endotracheal Tube used today? ☐₀ No ☐₁ Yes

   3a. If **Yes**, select ventilation mode and record the associated values at 1200 (Noon) or closest recorded data to 12 (Noon) if noon data are not available.

   ☐ Conventional Mechanical Ventilation (CMV):
   Mean Airway Pressure (MAP): __ __ . __ cmH_2O
   Positive End Expiratory Pressure (PEEP): __ __ cmH_2O

   ☐ High Frequency Oscillation:
   MAP: __ __ . __ cmH_2O

   ☐ High Frequency Jet Ventilation:
   MAP: __ __ . _ cmH_2O

4. Was Respiratory support without Endotracheal Tube used today? ☐₀ No ☐₁ Yes

   4a. If **Yes**, select ventilation mode and record the associated values at 1200 (Noon) or closest recorded data to 12 (Noon) if noon data are not available.

   ☐ Nasal Intermittent Mandatory Ventilation (NIMV):
   MAP: __ __ . __cmH_2O
   PEEP: __ __ cmH_2O

   ☐ Continuous Positive Airway Pressure (CPAP):
   CPAP: __ __ cmH_2O

   ☐ Nasal Cannula with flow rate:
   Nasal Cannula Flow: __ __ . __ __ __ __ Lpm

5. Was inhaled nitric oxide given today? ☐₀ No ☐₁ Yes

   5a. If **Yes**, record the concentration at 1200 (Noon) or closest recorded data to 12 (Noon) if noon data are not available. __ __ ppm

6. Was the baby reintubated today? ☐₀ No ☐₁ Yes

   6a. If **Yes**, indicate the primary reason why the baby was reintubated:
   ☐₁ Increasing respiratory distress
   ☐₂ Stridor
   ☐₃ Apnea and Bradycardia
   ☐₄ Suspected infection
   ☐₅ For diagnostic or therapeutic procedures, including surgery
   ☐₆ Unplanned extubation(s), indicate the number of occurrences this day: __
   ☐₉₈ Other, specify: _________________________

PREMATURITY AND RESPIRATORY OUTCOMES PROGRAM

SPECIMEN COLLECTION FORM

1. Collection Date ____/____/______ [mm/dd/yyyy]
2. Collection Time (if applicable) ___ ___ ___ ___ [24 hour clock]
3. Type of Specimen
   - ☐1 Infant Tracheal Aspirate
   - ☐2 Infant Urine
   - ☐3 Infant Saliva for DNA
   - ☐4 Mother Saliva for DNA
   - ☐5 Father Saliva for DNA
4. Laboratory Accession Number (from TA or UR vial label) __ __ __ (Site #) __ __ (Sample Type) __ __ __ __ (Sample #)

   4a. Enter Tracheal Aspirate **CL** Lab Accession Number __ __ __ (Site #) __ __ (Sample Type) __ __ __ __ (Sample #)
5. Number of Aliquots (for Tracheal Aspirate Supernatant or Urine) ____

   5a. Date Tracheal Aspirate or Urine Specimen Frozen ____/____/______ [mm/dd/yyyy]

   5b. Time Tracheal Aspirate or Urine Specimen Frozen: ___ ___ ___ ___ [24 hour clock]
6. Date sample shipped to Core Lab: ____/____/______ [mm/dd/yyyy]

*Sample Type

TA	Tracheal Aspirate Supernatant; send to UCSF Tracheal Aspirate Core Lab
CL	Tracheal Aspirate Cell Pellet; send to UCSF Tracheal Aspirate Core Lab
UR	Urine; send to Vanderbilt Urine Core Lab
DN	DNA (Saliva); send to Vanderbilt DNA Core Lab.
