# Supplementary material for: Prematurity and respiratory outcomes program (PROP): study protocol of a prospective multicenter study of respiratory outcomes of preterm infants in the United States
Source: BMC Pediatr. 2015 Apr 10;15:37. doi: 10.1186/s12887-015-0346-3 (PMC4407843; doi:10.1186/s12887-015-0346-3)
Supplement: Additional file 4: — Prematurity and respiratory outcomes program. Comorbidities of prematurity. [file 12887_2015_346_MOESM4_ESM.pdf]

Cardio-Pulmonary

1. Did the baby have any of the following types of air leaks? ☐0 No ☐1 Yes

   If **Yes**, indicate the type(s) of air leak by answering Questions 1a-1d:

   1a. Pneumothorax: ☐0 No ☐1 Yes
   If Yes, complete $1a_1$ and $1a_2$
   $1a_1$. Was a chest tube placed? ☐0 No ☐1 Yes

   $1a_2$. Has there been evidence of a bronchopleural fistula? ☐0 No ☐1 Yes

   1b. Pulmonary Interstitial Emphysema (PIE): ☐0 No ☐1 Yes

   1c. Pneumomediastinum: ☐0 No ☐1 Yes

   1d. Pneumopericardium: ☐0 No ☐1 Yes

2. Did the baby have any pulmonary hemorrhages? ☐0 No ☐1 Yes

   If **Yes**, complete 2a and 2b.

   2a. Did these hemorrhages require transfusion of blood products? ☐0 No ☐1 Yes

   2b. Did these hemorrhages require increased concentrations of supplemental oxygen and / or ventilator support? ☐0 No ☐1 Yes

3. Did the baby have a clinical diagnosis of Patent Ductus Arteriosus (PDA)? ☐0 No ☐1 Yes

   3a. Was the PDA confirmed by echocardiogram? ☐0 No ☐1 Yes

   Indicate if any of the following treatments were used to treat the suspected or confirmed PDA:

   3b. Indomethacin (do not report any prophylactic Indomethacin given within the first 24 hours of life): ☐0 No ☐1 Yes

   If **Yes**, indicate the first date of each distinct course of Indomethacin:

First Course	Second Course	Third Course
__ __ / __ __ / __ __ __ __ (Month / Day / Year)	__ __ / __ __ / __ __ __ __ (Month / Day / Year)	__ __ / __ __ / __ __ __ __ (Month / Day / Year)

   3c. Ibuprofen: ☐0 No ☐1 Yes

   If **Yes**, indicate the first date of each distinct course of Ibuprofen:

First Course	Second Course	Third Course
__ __ / __ __ / __ __ __ __ (Month / Day / Year)	__ __ / __ __ / __ __ __ __ (Month / Day / Year)	__ __ / __ __ / __ __ __ __ (Month / Day / Year)

3d. Surgical Ligation: ☐0 No ☐1 Yes

If **Yes**, date of surgery: __ __ / __ __ / __ __ __ __ (Month / Day / Year)

4. Was a diagnosis of pulmonary hypertension made by a pediatric cardiologist? ☐0 No ☐1 Yes

4a. If **Yes**, was this diagnosis based on (Check all that apply)

☐ Echocardiogram __ __ / __ __ / __ __ __ __ (Month / Day / Year)
Date of Diagnosis

☐ Cardiac catheterization __ __ / __ __ / __ __ __ __ (Month / Day / Year)
Date of Diagnosis

5. Was airway endoscopy performed by an ENT surgeon or pediatric pulmonologist? ☐0 No ☐1 Yes

5a. If **Yes**, indicate clinical findings (check all that apply):

☐ No abnormality noted

	1st Procedure	2nd Procedure
☐ Tracheomalacia	__ __ / __ __ / __ __ __ __ (Month / Day / Year)	__ __ / __ __ / __ __ __ __ (Month / Day / Year)
☐ Laryngomalacia	__ __ / __ __ / __ __ __ __ (Month / Day / Year)	__ __ / __ __ / __ __ __ __ (Month / Day / Year)
☐ Subglottic stenosis	__ __ / __ __ / __ __ __ __ (Month / Day / Year)	__ __ / __ __ / __ __ __ __ (Month / Day / Year)
☐ Vocal cord paralysis (unilateral)	__ __ / __ __ / __ __ __ __ (Month / Day / Year)	__ __ / __ __ / __ __ __ __ (Month / Day / Year)
☐ Vocal cord paralysis (bilateral)	__ __ / __ __ / __ __ __ __ (Month / Day / Year)	__ __ / __ __ / __ __ __ __ (Month / Day / Year)
☐ Other, specify: ____________________	__ __ / __ __ / __ __ __ __ (Month / Day / Year)	__ __ / __ __ / __ __ __ __ (Month / Day / Year)

6. Did the baby have a tracheotomy? ☐0 No ☐1 Yes

6a. If **Yes,** indicate the procedure date: __ __ / __ __ / __ __ __ __ (Month / Day / Year)

7. Was Ventilator-Associated Pneumonia (VAP) suspected in this baby? ☐$_0$ No ☐$_1$ Yes

If **Yes** to 7, answer 7a – 7c.

7a. Did the baby have worsening gas exchange (e.g. O_2 desaturations, Increased oxygen requirements, or increased ventilator demand)? ☐$_0$ No ☐$_1$ Yes

7b. Did the baby have any of the following associated conditions (check all that apply)?
- ☐ Temperature instability with no other recognized cause
- ☐ Leukopenia (<4,000 WBC/mm^3)
- ☐ Leukocytosis (≥ 15,000 WBC/mm^3) and lef shift (≥ 10% band forms)
- ☐ New onset of purulent sputum
- ☐ Change in character of sputum
- ☐ Increased respiratory secretions
- ☐ Increased suctioning requirements
- ☐ Apnea
- ☐ Tachypnea
- ☐ Nasal flaring with retraction of chest wall or grunting
- ☐ Wheezing
- ☐ Rales
- ☐ Rhonchi
- ☐ Cough
- ☐ Bradycardia (< 100 beats per minute)
- ☐ Tachycardia (> 170 beats per minute)
- ☐ None of the above

7c. Did the baby have serial chest radiographs with any of the following (check all that apply)?
- ☐ New infiltrate
- ☐ Progressive and persistent infiltrate
- ☐ Consolidation
- ☐ Cavitation
- ☐ Pneumatoceles
- ☐ None of the above

Infection

8. Did the baby have any blood culture-proven Sepsis? ☐$_0$ No ☐$_1$ Yes

8a. If **Yes**, indicate the type(s) of Sepsis:
- ☐ Bacterial Number of distinct episodes: ___
- ☐ Fungal Number of distinct episodes: ___
- ☐ Viral Number of distinct episodes: ___

8b. Presumed, but not culture-proven Sepsis? ☐$_0$ No ☐$_1$ Yes

If **Yes**, number of distinct episodes: ___

9. Did the baby have any culture-proven Meningitis? ☐$_0$ No ☐$_1$ Yes

9a. If **Yes**, indicate the type(s) of Meningitis:
- ☐ Bacterial Number of distinct episodes: ___
- ☐ Fungal Number of distinct episodes: ___
- ☐ Viral Number of distinct episodes: ___

9b. Presumed, but not culture-proven Meningitis? ☐$_0$ No ☐$_1$ Yes

If **Yes**, number of distinct episodes: ___

10. Did the baby have upper respiratory tract infection of confirmed viral etiology? ☐$_0$ No ☐$_1$ Yes

10a. If **Yes**, indicate the confirmed viral etiologies (check all that apply):

	1st Diagnosis	2nd Diagnosis
☐ Influenza	__ __ / __ __ / __ __ __ __ (Month / Day / Year)	__ __ / __ __ / __ __ __ __ (Month / Day / Year)
☐ Para-influenza	__ __ / __ __ / __ __ __ __ (Month / Day / Year)	__ __ / __ __ / __ __ __ __ (Month / Day / Year)
☐ Rhinovirus	__ __ / __ __ / __ __ __ __ (Month / Day / Year)	__ __ / __ __ / __ __ __ __ (Month / Day / Year)
☐ Respiratory syncytial virus (RSV)	__ __ / __ __ / __ __ __ __ (Month / Day / Year)	__ __ / __ __ / __ __ __ __ (Month / Day / Year)
☐ Other, specify ____________________	__ __ / __ __ / __ __ __ __ (Month / Day / Year)	__ __ / __ __ / __ __ __ __ (Month / Day / Year)

11. Did the baby have any other infections? ☐$_0$ No ☐$_1$ Yes

11a. If **Yes**, indicate the infections (check all that apply):

	1st Diagnosis	2nd Diagnosis
☐ Urinary tract infection	__ __ / __ __ / __ __ __ __ (Month / Day / Year)	__ __ / __ __ / __ __ __ __ (Month / Day / Year)
☐ Cellulitis	__ __ / __ __ / __ __ __ __ (Month / Day / Year)	__ __ / __ __ / __ __ __ __ (Month / Day / Year)
☐ Osteomyilitis	__ __ / __ __ / __ __ __ __ (Month / Day / Year)	__ __ / __ __ / __ __ __ __ (Month / Day / Year)
☐ Cytomegalovirus (CMV)	__ __ / __ __ / __ __ __ __ (Month / Day / Year)	__ __ / __ __ / __ __ __ __ (Month / Day / Year)
☐ Surgical wound infection	__ __ / __ __ / __ __ __ __ (Month / Day / Year)	__ __ / __ __ / __ __ __ __ (Month / Day / Year)
☐ Other, specify ____________________	__ __ / __ __ / __ __ __ __ (Month / Day / Year)	__ __ / __ __ / __ __ __ __ (Month / Day / Year)

Gastro Intestinal

12. Did the baby have Necrotizing Enterocolitis (NEC) Bell stage 2 or 3? ☐0 No ☐1 Yes

12a. If **Yes**, date of first medical diagnosis: __ __ / __ __ / __ __ __ __ (Month / Day / Year)

12b. Were there any bowel perforations? ☐0 No ☐1 Yes

12c. Did the baby have surgery for NEC? ☐0 No ☐1 Yes

If **Yes**, indicate the surgical procedure(s) performed (check all that apply) and provide the date(s) of surgery:

Procedure	1st Surgery	2nd Surgery
☐ Peritoneal drain	__ __ / __ __ / __ __ __ __ (Month / Day / Year)	__ __ / __ __ / __ __ __ __ (Month / Day / Year)
☐ Laparotomy	__ __ / __ __ / __ __ __ __ (Month / Day / Year)	__ __ / __ __ / __ __ __ __ (Month / Day / Year)
☐ Bowel resection	__ __ / __ __ / __ __ __ __ (Month / Day / Year)	__ __ / __ __ / __ __ __ __ (Month / Day / Year)
☐ Repair of Adhesion / Strictures	__ __ / __ __ / __ __ __ __ (Month / Day / Year)	__ __ / __ __ / __ __ __ __ (Month / Day / Year)

13. Did the baby have any Isolated Bowel Perforations not considered to be associated with NEC? ☐0 No ☐1 Yes

13a. If **Yes**, date of first medical diagnosis: __ __ / __ __ / __ __ __ __ (Month / Day / Year)

13b. Did the baby receive any surgery for Isolated Bowel Perforations not considered to be associated with NEC? ☐0 No ☐1 Yes

If **Yes**, indicate the surgical procedure(s) performed (check all that apply) and provide the date(s) of surgery:

Procedure	1st Surgery	2nd Surgery
☐ Peritoneal drain	__ __ / __ __ / __ __ __ __ (Month / Day / Year)	__ __ / __ __ / __ __ __ __ (Month / Day / Year)
☐ Laparotomy	__ __ / __ __ / __ __ __ __ (Month / Day / Year)	__ __ / __ __ / __ __ __ __ (Month / Day / Year)

Ophthalmologic

14. Were any Retinopathy of Prematurity (ROP) examinations performed prior to discharge from the PROP study center? ☐0 No ☐1 Yes

15. Was this baby diagnosed with ROP? ☐0 No ☐1 Yes

If **Yes**, answer the following questions:

15a. What was the worst stage ever reported in any zone?
__ Left Eye (1-5)
__ Right Eye (1-5)

15b. Did the baby undergo laser or cryo-surgery?

Left Eye: ☐0 No ☐1 Yes Date of procedure: __ __ / __ __ / __ __ __ __ (Month / Day / Year)

Right Eye: ☐0 No ☐1 Yes Date of procedure: __ __ / __ __ / __ __ __ __ (Month / Day / Year)

15c. Did the baby undergo Bevacizumab (Avastin) treatment?

Left Eye: ☐0 No ☐1 Yes Date of procedure: __ __ / __ __ / __ __ __ __ (Month / Day / Year)

Right Eye: ☐0 No ☐1 Yes Date of procedure: __ __ / __ __ / __ __ __ __ (Month / Day / Year)

15d. Did the baby undergo vitrectomy?

Left Eye: ☐0 No ☐1 Yes Date of procedure: __ __ / __ __ / __ __ __ __ (Month / Day / Year)

Right Eye: ☐0 No ☐1 Yes Date of procedure: __ __ / __ __ / __ __ __ __ (Month / Day / Year)

Neurologic

16. Did the baby receive a ventricular shunt? ☐0 No ☐1 Yes

16a. If **Yes**, provide the date of first shunt placement: __ __ / __ __ / __ __ __ __ (Month / Day / Year)

Other Surgeries (excluding PDA ligation, surgery for NEC or Bowel Perforation, all surgeries for ROP, ventriculoperitoneal shunt placement, and tracheotomy)

1. Type of surgery: ______________________ Date of surgery: __ __ / __ __ / __ __ __ __ (Month / Day / Year)
2. Type of surgery: ______________________ Date of surgery: __ __ / __ __ / __ __ __ __ (Month / Day / Year)
3. Type of surgery: ______________________ Date of surgery: __ __ / __ __ / __ __ __ __ (Month / Day / Year)
4. Type of surgery: ______________________ Date of surgery: __ __ / __ __ / __ __ __ __ (Month / Day / Year)
5. Type of surgery: ______________________ Date of surgery: __ __ / __ __ / __ __ __ __ (Month / Day / Year)

1. What was the baby's discharge date? __ __ / __ __ / __ __ __ __ (Month / Day / Year)

2. Where was the baby discharged to?
   - ☐1 Home
   - ☐2 Transfer to another hospital
   - ☐3 Baby died at study center*
   - ☐98 Other, specify: ______________________

NOTE: If the child dies at the study center, record date of discharge as date of death and complete a Death Form. All remaining information on this form should reflect information collected up to the time of death.

3. Was this baby enrolled in TOLSURF? ☐0 No ☐1 Yes
   3a. If **Yes**, provide study specific Participant ID ______________________

4. Was this baby enrolled in the NRN Hydrocortisone for Extubation Trial? ☐0 No ☐1 Yes
   4a. If **Yes**, provide study specific Participant ID ______________________

5. Was this baby enrolled in the NRN Generic database? ☐0 No ☐1 Yes
   5a. If **Yes**, provide study specific Participant ID ______________________

6. Was this baby enrolled in any other randomized clinical treatment trials? ☐0 No ☐1 Yes
   6a. If **Yes**, provide clinical trial name(s): ______________________

7. Was this baby enrolled in any other long term follow-up studies? ☐0 No ☐1 Yes
   7a. If **Yes**, provide study name(s): ______________________

8. How many people normally live in your home including your baby (for at least 6 months of the year)? (Please select one)
   - ☐1 2-3
   - ☐2 4-6
   - ☐3 7-10
   - ☐4 >10

   8a. How many other children under 5 years old live in <baby's name>'s home? ___ ___ children

   8b. How many children between ages 5-12 years old live in <baby's name>'s home? ___ ___ children

9. Is baby exposed to dogs, cats, or other furry animals at home? ☐0 No ☐1 Yes

10. Will your baby receive any care outside of the home in the next year? ☐$_0$ No ☐$_1$ Yes ☐$_{88}$ Unknown

11. Which one of the following three statements best describes smoking in <baby's name>'s home?

☐$_1$Smoking is allowed anywhere in the home
☐$_2$Smoking is limited to part of the house where <baby's name> rarely goes
☐$_3$Smoking is not allowed inside the home at all

12. Which one of the following five statements best describes smoking in the car?

☐$_0$Child rarely travels by car.
☐$_1$There is no smoking inside the car
☐$_2$Smoking occurs in the car only when <baby's name> is not inside
☐$_3$Smoking is sometimes allowed in the car
☐$_4$ Smoking is usually or always allowed in the car

13. Please tell us what breathing and allergy problems run in the family (Check all that apply)

Symptoms	None/ Not applicable	Biological Siblings (any)	Biological Parents (one or both)
a. Asthma/Recurrent lung infections	☐$_0$	☐$_1$	☐$_2$
b. Allergies/Hayfever	☐$_0$	☐$_1$	☐$_2$
c. Eczema	☐$_0$	☐$_1$	☐$_2$

14. How will <baby's name>'s health care be paid for primarily: (select only one)

☐$_0$ No Insurance (self pay)
☐$_1$ Private Insurance
☐$_2$ Medicaid/Public Insurance

Please Note: This is an Administrative Form. All personal information provided will be held in confidence and will not be entered into the Data Management System Database.

1. Child's name: ______________ First ______________ Last
   a. Child's nickname (if any) ______________
   b. Child's sex ☐ Male ☐ Female
2. Child's birth date: __ __ / __ __ / __ __ __ __ (Month / Day / Year)
3. Telephone Number: ______ - ______ - ______
4. Address: ______________

____

____

Primary Contact Information

5. Name: ______________ First ______________ Last
6. Telephone Number: ______ - ______ - ______
7. Cell Phone Number: ______ - ______ - ______
8. Address: ______________

____

____

9. Email address: ______________

Mother's Contact Information

10. Is this the Primary Contact? ☐ No (Complete contact information) ☐ Yes (Skip to next section)
11. Name: ______________ First ______________ Last
12. Telephone Number: ______ - ______ - ______
13. Cell Phone Number: ______ - ______ - ______
14. Address: ______________

____

____

15. Email address: ______________

Spouse/Partner's Contact Information

16. Is this the Primary Contact? ☐ No (Complete contact information) ☐ Yes (Skip to next section)
17. Name: ______________ First ______________ Last
18. Telephone Number: ______ - ______ - ______
19. Cell Phone Number: ______ - ______ - ______
20. Address: ______________________________
____
____
21. Email address: ______________________________

Please provide the name and contact information of someone who is most likely to have your address in case we lose contact with you. It is helpful if this person does not reside with your child.

22. Name: ______________ First ______________ Last
23. Telephone Number: ______ - ______ - ______
24. Cell Phone Number: ______ - ______ - ______
25. Address: ______________________________
____
____
26. Email address: ______________________________

Please provide the name and contact information of an additional person who is most likely to have your address in case we lose contact with you.

27. Name: ______________ First ______________ Last
28. Telephone Number: ______ - ______ - ______
29. Cell Phone Number: ______ - ______ - ______
30. Address: ______________________________
____
____
31. Email address: ______________________________

Pediatrician Contact Information

32. Name: ______________________ ______________________
First Last

33. Telephone Number: ______ - ______ - ______

34. Cell Phone Number: ______ - ______ - ______

35. Address: ______________________________
____
____

36. Email address: ______________________________

Other Health Professional's Contact Information

37. Name: ______________________ ______________________
First Last

38. Telephone Number: ______ - ______ - ______

39. Cell Phone Number: ______ - ______ - ______

40. Address: ______________________________
____
____

41. Email address: ______________________________
