# Supplementary material for: Prematurity and respiratory outcomes program (PROP): study protocol of a prospective multicenter study of respiratory outcomes of preterm infants in the United States
Source: BMC Pediatr. 2015 Apr 10;15:37. doi: 10.1186/s12887-015-0346-3 (PMC4407843; doi:10.1186/s12887-015-0346-3)
Supplement: Additional file 5: — Prematurity and respiratory outcomes program. Standard visit and follow up surveys. [file 12887_2015_346_MOESM5_ESM.pdf]

** Please Note: All efforts should be made to conduct the interview with the child's primary caregiver or the individual who is able to provide information about the child's health since the <last contact>. **

1. Was this interview conducted? ☐0 No (Skip to 1c) ☐1 Yes (Continue with 1a)

   If Yes, answer questions 1a and 1b.

   a. Visit month ☐1 Month 3 ☐2 Month 6 ☐3 Month 9 ☐4 Month 12

   b. Date of Interview: __ __ / __ __ / __ __ __ __ (Month / Day / Year)

   c. If no, indicate reason why interview was not conducted (Please select only one)
      - ☐1 Unable to contact
      - ☐2 Refused interview
      - ☐3 Child died (Please complete Record of Death form)
      - ☐98 Other, specify ______________________

2. Initials of person completing this form ___ ___ ___

PID: __ __ __ __ __ __ __

DATE: __ __ / __ __ / __ __ __ __

1. Please indicate how interview was conducted. — ☐1 Over the phone ☐2 In person ☐3 In hospital

Section I: Hospitalizations and Urgent Care Visits
Since our <last contact> with you about <baby's name>…

2. How many times has <baby's name> been admitted to a hospital for one or more nights in a row outside of the Emergency Room? — __ __ times ☐88 Don't Know
   a. How many times were because of wheezing, breathing problems or a change in his/her breathing? — __ __ times ☐88 Don't Know
   b. Did any of these times require admission to an intensive care unit (NICU, PICU, or Critical Care Unit)? — ☐0 No ☐1 Yes ☐88 Don't Know
   c. Were any of the admissions due to Respiratory Syncytial Virus (RSV)? — ☐0 No ☐1 Yes ☐88 Don't Know

3. How many times has <baby's name> had a sick visit to a doctor's office, clinic or Emergency Room? — __ __ times ☐88 Don't Know
   a. How many times were because of wheezing, breathing problems or a change in his/her breathing? — __ __ times ☐88 Don't Know

Section II: Breathing, Wheezing, and Coughing Assessment

4. Has <baby's name>'s chest sounded wheezy or whistling? — ☐0 No ☐1 Yes ☐88 Don't Know

   If Yes, answer questions a-d.
   a. Has this occurred with colds? — ☐0 No ☐1 Yes ☐88 Don't Know
   b. Has <baby's name>'s chest sounded wheezy or whistling apart from colds? — ☐0 No ☐1 Yes ☐88 Don't Know
   c. How often has <baby's name>'s chest sounded wheezy during the day time?
      - ☐0 Less than once per week
      - ☐1 1-2 times per week
      - ☐2 3-6 times per week
      - ☐3 Daily, but not all the time
      - ☐4 Daily, all the time
   d. How often has <baby's name>'s chest sounded wheezy during the night time?
      - ☐0 Less than once per week
      - ☐1 1-2 times per week
      - ☐2 3-6 times per week
      - ☐3 Daily, but not all the time
      - ☐4 Daily, all the time

5. Since our <last contact> has <baby's name> been diagnosed with wheezing by a doctor? — ☐0 No ☐1 Yes ☐88 Don't Know

Since our <last contact> with you about <baby's name>…

6. Has <baby's name> had a cough without a cold? ☐$_0$ No ☐$_1$ Yes ☐$_{88}$ Don't Know

If Yes, answer questions a-b.

a. How often has <baby's name> had coughing during the day time?
- ☐$_0$ Less than once per week
- ☐$_1$ 1-2 times per week
- ☐$_2$ 3-6 times per week
- ☐$_3$ Daily, but not all the time
- ☐$_4$ Daily, all the time

b. How often has <baby's name> had coughing during the night time?
- ☐$_0$ Less than once per week
- ☐$_1$ 1-2 times per week
- ☐$_2$ 3-6 times per week
- ☐$_3$ Daily, but not all the time
- ☐$_4$ Daily, all the time

Since our <last contact> with you about <baby's name>…

7. How many head colds (common colds) has <baby's name> had?
- ☐$_0$ 0
- ☐$_1$ 1
- ☐$_2$ 2
- ☐$_3$ 3
- ☐$_4$ 4 or more

Section III: Respiratory Medications

[Please ask parent to collect all medications that the baby is currently using or has used since [discharge or the last contact] and identify them by reading the medication label or package. Record the medication name (generic or brand name) in the Respiratory Medication worksheet. There is no need to record dose, unit, frequency or route of administration. Use the Respiratory Medication worksheet as a guide to determine which medications should be recorded in the database.

Please complete the Follow Up Medication Log (MEDLOG) in the database.]

Section IV: Home Technology Dependence

8. Since our <last contact> has <baby's name> used any medical equipment or any of the following in the home?

a. Breathing and Heart Rate monitor	☐$_0$ No	☐$_1$ Yes
b. Oxygen therapy	☐$_0$ No	☐$_1$ Yes
c. CPAP or BIPAP	☐$_0$ No	☐$_1$ Yes
d. Ventilator	☐$_0$ No	☐$_1$ Yes
e. Trach or breathing tube	☐$_0$ No	☐$_1$ Yes
f. Feeding Tube in nose	☐$_0$ No	☐$_1$ Yes
g. Feeding Tube in stomach	☐$_0$ No	☐$_1$ Yes
h. Other ______________________________	☐$_0$ No	☐$_1$ Yes

NOTE – The Month 3 and Month 9 questionnaires end here. Complete the entire form at Months 6 and 12.

Section V: Nutrition

9. Since our <last contact>, did <baby's name> receive mother's breast milk, either at breast, from a bottle or through a tube? ☐$_0$ No ☐$_1$ Yes ☐$_{88}$ Don't Know
   a. If Yes, for how many months did <baby's name> receive any breast milk for more than half of the feedings?
      ☐$_0$ Less than 1
      ☐$_1$ 1
      ☐$_2$ 2
      ☐$_3$ 3

Section VI: Exposure to Tobacco products and Respiratory Irritants

10. How often has the mother or primary caregiver smoked in the in the last 6 months?
    ☐$_0$ Never
    ☐$_1$ Monthly
    ☐$_2$ Weekly
    ☐$_3$ Daily
11. How many people who live in <baby's name>'s home smoke? ___ ___ people
12. Which one of the following three statements best describes smoking in <baby's name>'s home?
    ☐$_1$ Smoking is allowed anywhere in the home
    ☐$_2$ Smoking is limited to part of the house where <baby's name> rarely goes
    ☐$_3$ Smoking is not allowed inside the home at all
13. Which one of the following five statements best describes smoking in the car?
    ☐$_0$ Child rarely travels by car
    ☐$_1$ There is no smoking inside the car
    ☐$_2$ Smoking occurs in the car only when <baby's name> is not inside
    ☐$_3$ Smoking is sometimes allowed in the car
    ☐$_4$ Smoking is usually or always allowed in the car
14. a. How many other children under 5 years old live in <baby's name>'s home? ___ ___ children
    b. How many children between ages 5-12 years old live in <baby's name>'s home? ___ ___ children

Please indicate if <baby's name> is exposed to the following at HOME

15. Smoke from cigarettes or other tobacco products? ☐$_0$ No ☐$_1$ Yes
    a. If yes, how often is it used?
       ☐$_1$ Daily
       ☐$_2$ Weekly
       ☐$_3$ Monthly
16. Kerosene heater, wood burning stove, or fireplace ☐$_0$ No ☐$_1$ Yes
    a. If yes, how often is it used?
       ☐$_1$ Daily
       ☐$_2$ Weekly
       ☐$_3$ Monthly
17. Dogs, cats, and other furry animals ☐$_0$ No ☐$_1$ Yes
    a. If yes, how many pets? __ __ pets

18. Does your child receive regular care (at least once a week) outside the home? ☐0 No ☐1 Yes
    a. If Yes, are there children that are not siblings also present at the outside care site? ☐0 No ☐1 Yes

If response to Question 18 is Yes and <baby's name> attends DAY CARE, please answer questions 19-21, if not, please skip to question 22. Please indicate if <baby's name> is exposed to the following at DAY CARE

19. Smoke from cigarettes or other tobacco products? ☐0 No ☐1 Yes ☐99 N/A
    a. If yes, how often is it used? ☐1 Daily ☐2 Weekly ☐3 Monthly

20. Kerosene heater, wood burning stove, or fireplace ☐0 No ☐1 Yes ☐99 N/A
    a. If yes, how often is it used? ☐1 Daily ☐2 Weekly ☐3 Monthly

21. Dogs, cats, and other furry animals ☐0 No ☐1 Yes ☐99 N/A
    a. If yes, how many pets? __ __ pets

If response to Question 18 is Yes and <baby's name> goes to the HOME of a Babysitter or Other Regular Caregiver, please answer questions 22-24, if not, please skip to question 25.
Please indicate if <baby's name> is exposed to the following at the HOME of BABYSITTER or Other REGULAR CAREGIVER

22. Smoke from cigarettes or other tobacco products? ☐0 No ☐1 Yes ☐99 N/A
    a. If yes, how often is it used? ☐1 Daily ☐2 Weekly ☐3 Monthly

23. Kerosene heater, wood burning stove, or fireplace ☐0 No ☐1 Yes ☐99 N/A
    a. If yes, how often is it used? ☐1 Daily ☐2 Weekly ☐3 Monthly

24. Dogs, cats, and other furry animals ☐0 No ☐1 Yes ☐99 N/A
    a. If yes, how many pets? __ __ pets

Section VII: Respiratory Treatments

25. Has <baby's name> had regular shots to prevent Respiratory Syncytial Virus (RSV)? ☐0 No ☐1 Yes ☐88 Don't Know

26. Has <baby's name> had a flu shot? ☐0 No ☐1 Yes ☐88 Don't Know
☐99 N/A (for children < 6 months chronological age)

27. Are <baby's name>'s immunizations up to date? ☐0 No ☐1 Yes ☐88 Don't Know
   a. If not, why not?
   ☐ Illness
   ☐ Refused
   ☐ Other

28. How much does <baby's name>'s health and healthcare needs disrupt your own lifestyle or other planned activities?
(For example: ability to attend work or school)
☐0 Not at all
☐1 Infrequently
☐2 Half the time
☐3 Frequently
☐4 All of the time

Section VIII: Infant Gastroesophageal Reflux Questionnaire (I-GERQ-R)
Please complete the Gastroesophageal Reflux Questionnaire

Section IX: Atopy and Allergy Assessment (Completed at Month 12 ONLY)

29. Has <baby's name> had a new diagnosis of asthma or reactive airways disease diagnosed by a doctor? ☐0 No ☐1 Yes ☐88 Don't Know

30. Has <baby's name> had a runny, stuffy, or itchy nose, or watery eyes apart from a cold? ☐0 No ☐1 Yes ☐88 Don't Know

31. Has <baby's name> ever been allergic to any food? ☐0 No ☐1 Yes ☐88 Don't Know

32. Has <baby's name> been diagnosed with eczema (allergic skin rash)? ☐0 No ☐1 Yes ☐88 Don't Know

PREMATURITY AND RESPIRATORY OUTCOMES PROGRAM

PID: __ __ __ __ __ __ __

INFANT GASTROESOPHAGEAL REFLUX QUESTIONNAIRE (I-GERQ) [1]

DATE: __ __ / __ __ / __ __ __ __

1. During the past week, how often did the baby spit-up (anything coming out of the mouth) during a 24-hour period?
   - ☐ Less than once
   - ☐ 1 to 3 times
   - ☐ 4 to 6 times
   - ☐ More than 6 times

2. During the past week, how much did the baby spit-up (anything coming out of the mouth) during a typical episode?
   - ☐ Did not spit up
   - ☐ Less than 1 tablespoonful
   - ☐ 1 tablespoonful to 2 ounces
   - ☐ More than 2 ounces to half the feeding
   - ☐ More than half the feeding

3. During the past week, how often did spitting up (anything coming out of the mouth) seem to be uncomfortable for the baby, for example, crying, fussing, irritability, etc.?
   - ☐ Never
   - ☐ Rarely
   - ☐ Sometimes
   - ☐ Often
   - ☐ Always

4. During the past week, how often did the baby refuse a feeding even when hungry?
   - ☐ Never
   - ☐ Rarely
   - ☐ Sometimes
   - ☐ Often
   - ☐ Always

5. During the past week, how often did the baby stop eating soon after starting even when hungry?
   - ☐ Never
   - ☐ Rarely
   - ☐ Sometimes
   - ☐ Often
   - ☐ Always

6. During the past week, did the baby cry a lot during or within 1 hour after feedings?
   - ☐ Never
   - ☐ Rarely
   - ☐ Sometimes
   - ☐ Often
   - ☐ Always

7. During the past week, did the baby cry or fuss more than usual?
   - ☐ Never
   - ☐ Rarely
   - ☐ Sometimes
   - ☐ Often
   - ☐ Always

8. During the past week, on average how long did the baby cry or fuss during a 24 hour period?

- ☐ Less than 10 minutes
- ☐ 10 minutes to 1 hour
- ☐ More than 1 hour but less than 3 hours
- ☐ 3 or more hours

9. During the past week, how often did the baby have hiccups?

- ☐ Never
- ☐ Rarely
- ☐ Sometimes
- ☐ Often
- ☐ Always

10. During the past week, how often did the baby have episodes of arching back?

- ☐ Never
- ☐ Rarely
- ☐ Sometimes
- ☐ Often
- ☐ Always

11. During the past week, has the baby stopped breathing while awake or struggled to breathe?

- ☐ No
- ☐ Yes

12. During the past week, has the baby turned blue or purple?

- ☐ No
- ☐ Yes

[1] Translated versions of the I-GERQ-R are available on request from Dr. Susan Orenstein at the University of Pittsburgh (Susan.Orenstein@chp.edu)

Infant Gastroesophageal Reflux Questionnaire Revised (I-GERQ-R, Author I-GERQ, Susan Orenstein, MD, © 2004, University of Pittsburgh)

**Please ask parent to collect all medications that the baby is currently using or has used since [discharge or the last contact] and identify them by reading the medication label or package. Record the medication name (generic or brand name) on this Respiratory Medication worksheet. There is no need to record dose, unit, frequency or route of administration. Use the Respiratory Medication worksheet as a guide to determine which medications should be recorded in the database. Please complete the Follow Up Medication Log (MEDLOG) in the database. **

Respiratory Medication	Notes

Table 1. Respiratory medications with standardized drug names and codes for DMS

Generic Name	Commonly used Brand Names	Category	Drug Code
Albuterol (Nebulized or MDI)	Ventolin, Ventolin HFA, Proventil, Proventil HFA, Accuneb, Proair	Inhaled Bronchodilator	2073
Aminophylline	Aminophylline	Methylxanthine	37
Beclomethasone (MDI)	Beclomethasone Dipropionate, QVAR	Inhaled Steroid	70
Bosentan	Bosentan, Tracleer	Pulmonary Vasodilator	22990
Budesonide (Nebulized or MDI)	Pulmicort	Inhaled Steroid	6545
Bumetanide	Bumex	Diuretic	3664
Caffeine Citrate	Caffeine Citrate/ Cafcit Caffeine Solution	Methylxanthine	11
Chlorothiazide	Diuril	Diuretic	3646
Ciclesonide (MDI)	Alvesco	Inhaled Steroid	32691
Dexamethasone	Dexamethasone Intensol, Baycadron	Systemic Corticosteroid	2889
Dexamethasone (Nebulized)	Dexamethasone (Nebulized)	Inhaled Steroid	38645
Epoprostenol Sodium	Epoprostenol, Flolan	Pulmonary Vasodilator	37760
Flunisolide (MDI)	Flunisolide, Aerobid	Inhaled Steroid	72
Fluticasone (MDI)	Fluticasone Propionate, Flovent HFA	Inhaled Steroid	7873
Furosemide	Lasix	Diuretic	3660
Hydrochlorothiazide	Microzide	Diuretic	3649
Hydrocortisone	Cortef Hydrocortisone	Systemic Corticosteroid	2866
Iloprost Tromethamine	Iloprost, Ventavis Inhalation solution	Pulmonary Vasodilator	26287
Ipratropium bromide	Atrovent, Atrovent HFA,	Inhaled Bronchodilator	57
Levalbuterol (Nebulized or MDI)	Xopenex, Xopenex HFA, Xopenex Pediatric	Inhaled Bronchodilator	19858
Methylprednisolone	Depo-medrol, Solu-medrol, Medrol	Systemic Corticosteroid	2877
Metolazone	Zaroxolyn	Diuretic	3663
Mometasone (MDI)	Asmanex, Mometasone Furoate	Inhaled Steroid	3329
Montelukast	Montelukast, Singulair	Leukotriene receptor antagonist (LTRA)	16911

Table 1. Respiratory medications with standardized drug names and codes for DMS (continued)

Generic Name	Commonly used Brand Names	Category	Drug Code
Prednisone / Prednisolone	Prednisol, Flo-Pred, Millipred, Orapred, Orapred ODT, Pediapred, Prelone	Systemic Corticosteroid	2879
Racemic epinephrine (Nebulized)	Racepinephrine	Inhaled Bronchodilator	25002
Sildenafil	Sildenafil Citrate, Viagra, Revatio	Pulmonary Vasodilator	18084
Spironolactone	Aldactone	Diuretic	2901
Terbutaline	Terbutaline Sulfate	Bronchodilator	2071
Theophylline	Quibron-T, Theo-Dur, Theo-24, uniphyl, Theochron, Theolair, TheoCap	Methylxanthine	25
Treprostinil	Remodulin (parenteral)	Pulmonary Vasodilator	2360
Treprostinil	Tyvaso (inhaled)	Pulmonary Vasodilator	36541
Triamcinolone (MDI)	Azmacort	Inhaled Steroid	2891

*NOTE: Nasal formulations, topical ointments/creams and ophthalmologic drops or solutions should NOT be recorded in the database.

Table 2. DMS entry for combination medications

Drug Name	Standardized Drug Name	Drug Code
Aldactazide	Hydrochlorothiazide	3649
	Spironolactone	2901
Duoneb/Combivent	Albuterol	2073
	Ipratropium	57
Symbicort	Budesonide	6545
	Formoterol	10747
Dulera	Mometasone	3329
	Formoterol	10747
Advair	Fluticasone	7873
	Salmeterol	19963**

**Note: Code 19963 is a code for Advair as Salmeterol and Fluticasone. If Advair was given, please record Fluticasone (Code 7873) and Salmeterol (Code 19963) as separate rows.

PREMATURITY AND RESPIRATORY OUTCOMES PROGRAM

FOLLOW UP MEDICATION LOG

PID: __ __ __ __ __ __ __

DATE: __ __ / __ __ / __ __ __ __

Medication Sequence #	Drug Code	Drug Name (Brand Name or Generic)
