# Supplementary material for: Prematurity and respiratory outcomes program (PROP): study protocol of a prospective multicenter study of respiratory outcomes of preterm infants in the United States
Source: BMC Pediatr. 2015 Apr 10;15:37. doi: 10.1186/s12887-015-0346-3 (PMC4407843; doi:10.1186/s12887-015-0346-3)
Supplement: Additional file 6: — Prematurity and respiratory outcomes program. Additional medication log and miscellaneous forms. [file 12887_2015_346_MOESM6_ESM.pdf]

PREMATURITY AND RESPIRATORY OUTCOMES PROGRAM

ADDITIONAL MEDICATION LOG

PID: __ __ __ __ __ __ __

DATE: __ __ / __ __ / __ __ __ __

Record any "other" medication administered to the infant during hospitalization.

Medication Sequence Number	Drug Code	Drug Name

PREMATURITY AND RESPIRATORY OUTCOMES PROGRAM

ADVERSE EVENT LOG

PID: __ __ __ __ __ __ __

DATE: __ __ / __ __ / __ __ __ __

Please Note: Record Adverse Events associated with the respiratory tests performed specifically for the PROP study. AEs *recorded on other Case Report Forms should not be recorded in this Log.*

Adverse Event Sequence Number	Adverse Event Code	Adverse Event Description (Use for AE Codes 5 or 9)	Date of Onset (MM-DD-YYYY)	Outcome
			_ _/_ _/_ _ _ _	
			_ _/_ _/_ _ _ _	
			_ _/_ _/_ _ _ _	
			_ _/_ _/_ _ _ _	
			_ _/_ _/_ _ _ _	
			_ _/_ _/_ _ _ _	
			_ _/_ _/_ _ _ _	
			_ _/_ _/_ _ _ _	
			_ _/_ _/_ _ _ _	
			_ _/_ _/_ _ _ _	
			_ _/_ _/_ _ _ _	
			_ _/_ _/_ _ _ _	
			_ _/_ _/_ _ _ _	
			_ _/_ _/_ _ _ _	
			_ _/_ _/_ _ _ _	
			_ _/_ _/_ _ _ _	
			_ _/_ _/_ _ _ _	

<table>
<tr><th colspan="2">Adverse Event Codelist</th></tr>
<tr><td>Adverse Event Codes (AE)
1= Administration of supplemental oxygen of $FiO_2 \geq 0.10$ above baseline persisting for > 1 hour after test is concluded or terminated
2= Tachycardia (defined as Heart Rate > 200 beats per minute) persisting for > 1 hour after test is concluded or terminated.
3= Apnea: Cessation of breathing > 20 secs
4= Bradycardia: HR< 80 bpm >10 secs
5= Any additional event possibly or probably related to study procedure.</td><td rowspan="2">Outcome
1 = Recovered
2 = Recovered with Sequelae
3 = Not Recovered
4 = Fatal (Complete Record of Death)</td></tr>
<tr><td>Serious Adverse Event Codes (SAE)
6= Any need for CPR within 12 hours after the test is concluded or terminated.
7= Addition of mechanical ventilation within 12 hours after the test is concluded or terminated
8= Supplemental oxygen of $FiO_2 \geq 0.10$ above baseline persisting for ≥ 12 hours after test is concluded or terminated
9= Any additional event deemed to be serious by the site Principal Investigator and possibly or probably related to study procedure.
10= Death</td></tr>
</table>

Note: Apnea and bradycardia as defined here were reported in the initial study of RAC-type procedure for determination of BPD (Walsh 2003, 2004).
Also, persistent apnea despite stimulation or bradycardia (as defined above) are defined as reasons for failure of HCT, in addition to desaturation.

NOTE: Please indicate date of brain imaging exam in date field

1. Was brain imaging performed? ☐0 No ☐1 Yes

   1a. If **Yes**, indicate at which time:
   - ☐1 Within 7 days +/- 1 week after baby's birth
   - ☐2 Within 30 days +/- 1 week after baby's birth
   - ☐3 Between 34 Weeks and 40 Weeks Post-Menstrual Age

2. What imaging technique was used?
   - ☐1 Head Ultra Sound (HUS)
   - ☐2 Magnetic Resonance Imaging (MRI)

3. What were the results of the brain imaging, either the Worst HUS or MRI for this imaging? Check ALL that apply.
   - ☐ Normal
   - ☐ Subependymal hemorrhage (Grade 1 hemorrhage)
   - ☐ IVH without ventricular dilation (Grade 2 hemorrhage)
   - ☐ IVH distending at least one lateral ventricle (Grade 3 hemorrhage)
   - ☐ Intraparenchymal echodense lesion (Grade 4 hemorrhage)
   - ☐ Cystic Periventricular Leucomalacia (PVL)
   - ☐ Porencephalic cyst
   - ☐ Ventriculomegaly (with or without resolving IVH)
   - ☐ Cortical atrophy
   - ☐ Cerebellar hemorrhage
   - ☐ Other, specify*: ______________________

   ** Do NOT report normal variants. Examples of normal variants include: Cavum septi pellucidum, connatal cysts, isolated choroid plexus cysts.*

1. What was the baby's date of death? __ __ / __ __ / __ __ __ __ (Month / Day / Year)

2. What was the baby's primary cause of death?
(Specify cause of death from the death certificate*) ______________________________

3. Was an autopsy performed? ☐0 No ☐1 Yes ☐88 Unknown

3a. If **Yes**, what were the findings: ____________________________________

*If a death certificate is not available, please contact the primary care physician for a description of the events leading to death.

---

4. Please provide a short description of the infant's underlying condition prior to the events leading to the infant's death and the circumstances leading to the infant's death.

____

____

____

____

____

____

5. Do you think that a cardiopulmonary disorder contributed to the death of this infant? ☐0 No ☐1 Yes ☐88 Cannot make this determination

Signature of Principal Investigator: ______________________________ Date of Signature: __________

1. Collection Date ____/____/______ [mm/dd/yyyy]
2. Collection Time (if applicable) ___ ___ ___ ___ [24 hour clock]
3. Type of Specimen
   - ☐1 Infant Tracheal Aspirate
   - ☐2 Infant Urine
   - ☐3 Infant Saliva for DNA
   - ☐4 Mother Saliva for DNA
   - ☐5 Father Saliva for DNA
4. Laboratory Accession Number (from TA or UR vial label) __ __ __ (Site #) __ __ (Sample Type) __ __ __ __ (Sample #)

   4a. Enter Tracheal Aspirate **CL** Lab Accession Number __ __ __ (Site #) __ __ (Sample Type) __ __ __ __ (Sample #)
5. Number of Aliquots (for Tracheal Aspirate Supernatant or Urine) ____

   5a. Date Tracheal Aspirate or Urine Specimen Frozen ____/____/______ [mm/dd/yyyy]

   5b. Time Tracheal Aspirate or Urine Specimen Frozen: ___ ___ ___ ___ [24 hour clock]
6. Date sample shipped to Core Lab: ____/____/______ [mm/dd/yyyy]

*Sample Type

TA	Tracheal Aspirate Supernatant; send to UCSF Tracheal Aspirate Core Lab
CL	Tracheal Aspirate Cell Pellet; send to UCSF Tracheal Aspirate Core Lab
UR	Urine; send to Vanderbilt Urine Core Lab
DN	DNA (Saliva); send to Vanderbilt DNA Core Lab.

PID:__ __ __ __ __ __ __

STUDY STATUS

Date:__ __ / __ __ / __ __ __ __

NOTE: This form must be completed when the infant's participation in the study ends early.

1. Date of last contact? __ __ / __ __ / __ __ __ __ (Month / Day / Year)

2. Indicate the primary reason participation stopped:
   - ☐1 Unable to contact parents/caregivers
   - ☐2 Parents/Caregivers refuse further participation
   - ☐98 Other, specify ____________________________

PID: __ __ __ __ __ __ __

DATE: __ __ / __ __ / __ __ __ __

*****Please Note: This is an Administrative Form. All personal information provided will be held in confidence and will not be entered into the Data Management System Database.*****

1. Child's Name: ______________ First ______________ Last
2. Mother's Full Name ______________ First ______________ Last
3. Father's Full Name ______________ First ______________ Last
4. Infant Date of Birth __ __ / __ __ / __ __ __ __ (Month / Day / Year)
5. Date of relocation: __ __ / __ __ / __ __ __ __ (Month / Day / Year)
6. New Clinical Center ______________

   ☐ PROP Site OR ☐ Non-PROP Site
7. New Address: ______________

   ____

   ____
8. New Telephone Number: ______ - ______ - ______

New Family Physician/ Pediatrician:

9. Name: Dr ______________
10. Address: ______________

    ____

    ____
11. Phone: ______ - ______ - ______
12. Confirm the following:

    ☐ Parents have agreed to have the child seen at the new PROP Site

    ☐ Parents have consented to release child's medical records

    ☐ Child's medical records have been sent to the new PROP Site

______________ Signature from original PROP Site ______________ Date
