# Supplementary material for: Prematurity and respiratory outcomes program (PROP): study protocol of a prospective multicenter study of respiratory outcomes of preterm infants in the United States
Source: BMC Pediatr. 2015 Apr 10;15:37. doi: 10.1186/s12887-015-0346-3 (PMC4407843; doi:10.1186/s12887-015-0346-3)
Supplement: Additional file 7: — Prematurity and respiratory outcomes program. Noninvasive respiratory assessments [NIRA]. [file 12887_2015_346_MOESM7_ESM.pdf]

NOTE: Please indicate date of assessment in date field.

BASELINE DATA

1. Were RIP bands placed on the baby? ☐0 No ☐1 Yes
   1a. If 'No', please check primary reason infant missed test
   - ☐1 Baby was ineligible
   - ☐2 Baby was eligible but discharged
   - ☐3 Staff oversight/Staff not available to perform test
   - ☐98 Other, specify: ______________________________

2. Date PO feeds started ____/____/______ mm /dd/ yyyy

3. What was the baby's most recent body weight? __ __ __ __ gms

4. Baseline Heart Rate ________ Beats per minute

5. Respiratory Status: ☐1 No Respiratory Support ☐2 Nasal cannula ≤ 1 LPM

   5a. If 'Nasal cannula' is checked, provide oxygen flow: __.__ __ LPM __ __ __. __%

6. Nasogastric Tube (NGT) placement at the start of the test ☐1 In ☐2 Out

<u>OXYGENATION WHILE FEEDING [OWF]</u>

7. Was the Oxygenation While Feeding [OWF] test performed? ☐0 No ☐1 Yes
   7a. If 'No', please check primary reason infant missed test
   - ☐1 Baby was ineligible
   - ☐2 Baby was eligible but discharged
   - ☐3 Staff oversight/Staff not available to perform test
   - ☐4 Site does not perform OWF
   - ☐98 Other, specify: ______________________________

Please Note: If your site does not perform OWF, skip to question #11.

8. Caloric density: __ __ __ __ cal/oz

9. Start volume in bottle: __ __ __ mL

10. End volume in bottle: __ __ __ mL

Event Codes		
**P**= Feeding Position	**B**= Begin Feeding	**I**= Interruption of feeding
**R**= Resumed Feeding	**E**= End of Feeding or Bronchodilator	**D**= Disconnect
Q= Infant is in quiet sleep state	A= Infant is awake or active sleep	

RESPIRATORY INDUCTIVE PLETHYSMOGRAPHY [RIP] and OXYGENATION WHILE SLEEPING [OWS]

11. Was the baby's last feed >30 minutes ago? ☐0 No ☐1 Yes
12. Was the baby in quiet sleep state at the start of the test? ☐0 No ☐1 Yes
13. Was the Respiratory Inductive Plethysmography [RIP] test performed? ☐0 No ☐1 Yes

    13a. If 'No', please check primary reason infant missed test
    - ☐1 Baby was ineligible
    - ☐2 Baby was eligible but discharged
    - ☐3 Staff oversight/Staff not available to perform test
    - ☐4 Baby not in quiet sleep
    - ☐98 Other, specify: ______________________________

14. Was the Oxygenation While Sleeping [OWS] test performed? ☐0 No ☐1 Yes

    14a. If 'No', please check primary reason infant missed test
    - ☐1 Baby was ineligible
    - ☐2 Baby was eligible but discharged
    - ☐3 Staff oversight/Staff not available to perform test
    - ☐4 Baby not in quiet sleep
    - ☐98 Other, specify: ______________________________

BRONCHODILATOR [BD] ADMINISTRATION

15. Was bronchodilator administered to the baby? ☐0 No ☐1 Yes

    15a. If 'Not Done', please check primary reason infant missed test
    - ☐1 Baby was ineligible
    - ☐2 Baby was eligible but discharged
    - ☐3 Staff oversight/Staff not available to perform test
    - ☐98 Other, specify: ______________________________

16. Was entire bronchodilator dose given? ☐0 No ☐1 Yes
17. Treatment Response Heart Rate ________ BPM

POST- BRONCHODILATOR RIP DATA

18. Was bronchodilator administration terminated early? ☐0 No ☐1 Yes

    18a. If 'Yes', check primary reason for early termination:
    - ☐1 Infant woke up
    - ☐2 Hypoxemia
    - ☐3 Respiratory Distress
    - ☐4 Heart Rate exceeded 10% above baseline
    - ☐98 Other, please specify ______________________________

    18b. Total time of bronchodilator treatment. __ __ minutes

> Please Note: If any Adverse Events occurred during the assessments, please record the details in the Adverse Event Log (AE).

NOTE: Please indicate date of assessment in date field

BASELINE DATA

1. Indicate baby's post-menstrual age at time of assessment __ __ Weeks __ __ Day
2. What is the baby's body weight? __ __ __ __ gms
3. Baseline Respiratory Rate (RR) __ __ __ Breaths per minute
4. Baseline Heart Rate __ __ __ Beats per minute
5. Respiratory Status
   - ☐1 No Continuous Respiratory Support [No BPD]
   - ☐2 Nasal Cannula [Continue to question 5a]
   - ☐3 CPAP, SiPAP or MV [BPD]

   5a. Oxygen flow (nasal cannula only): __.__ __ LPM __ __ __. __%
6. SpO_2: __ __ __. __ %
7. Most recent PCO_2 __ __ __ ☐99 NA

   7a. Date of PCO_2 ____/____/______ mm/dd/yyyy

ROOM AIR CHALLENGE [RAC]/ OXYGEN and FLOW REDUCTION to ROOM AIR

8. Was the Room Air Challenge test performed? ☐0 No ☐1 Yes

   8a. If 'No' please check primary reason infant missed test
   - ☐1 Baby was ineligible [Complete item 8b.]
   - ☐2 Baby was eligible but discharged or transferred
   - ☐3 Staff oversight/Staff or equipment not available to perform test
   - ☐98 Other, specify: ______________________________

   8b. If ineligible, please check primary reason for ineligibility
   - ☐1 Baby was off all continuous support
   - ☐2 Baby was on higher level of support than nasal cannula
   - ☐3 Unable to maintain baseline saturation SpO_2 ≥90%
   - ☐4 40 weeks only: No BPD at 36 weeks (Off continuous support or passed RAC)

9. Did Infant arouse (active sleep or awake) during the observation period? ☐0 No ☐1 Yes

10. Please indicate the result of the Room Air Challenge test: ☐1 Pass [No PBD]
☐2 Fail [BPD] Complete items 10a-c

10a. Record time to fail test: _______ minutes

10b. Please indicate primary reason for test failure:
- ☐2 Desaturation <90% for 5 consecutive minutes
- ☐3 Desaturation <80% for 15 consecutive seconds
- ☐4 Apnea
- ☐5 Bradycardia
- ☐98 Other, specify: ______________________________

10c. Respiratory Support at Failure: __.__ __ LPM __ __ __. __%

11. Final Respiratory Rate _______ Breaths/minute

12. Final Heart Rate _______ Beats/minute

13. Final SpO_2 __ __ __ %

Note: If any Adverse Events occurred during the assessment, record the details in the Adverse Event Log (AE).

Complete this form for all infants on whom you initiate a Room Air Challenge test.

Instructions: Notify physician responsible for the infant's care (attending or fellow), and obtain MD order to perform room air challenge.

- Challenge should be done with a pulse oximeter that has been getting a good signal for at least 5 minutes.
- Position infant supine, with any feedings or medications given 30 minutes prior to evaluation.
- Infant in Quiet Sleep (QS) at study initiation (Criteria: 1) Eyes closed and without movement, 2) Steady breathing, 3) Not vocalizing, and 4) No twitching of limbs (startles acceptable))
- Baseline data collection is done in infant's current oxygen
- Record oxygen saturation every 1 minute x 5 minutes
- Assess if infant is in Quiet Sleep (QS) every 5 minutes

Baseline Measurements

Heart Rate: ___ ___ ___ (bpm)
RR: ___ ___ (breaths per minute. Count for a full minute.)

Baseline Data	FiO_2: __.__ __ Liter flow: __.__					
	0 min	1 min	2 min	3 min	4 min	5 min
Time	:	:	:	:	:	:
Saturation						
QS (Y/N)						

Continue to oxygen reduction phase if all saturations are ≥ 90%

If unable to maintain saturations ≥ 90% continue to monitor for additional 10 minutes

	6 min	7 min	8 min	9 min	10 min	11 min	12 min	13 min	14 min	15 min
Time	:	:	:	:	:	:	:	:	:	:
Saturation										
QS (Y/N)										

If infant fails to proceed to Oxygen and Flow reduction phase, record Infant as **Ineligible** and indicate reason for failure:

"Unable to maintain baseline SpO_2 ≥ 90%"

PREMATURITY AND RESPIRATORY OUTCOMES PROGRAM

ROOM AIR CHALLENGE WORKSHEET

PID: __ __ __ __ __ __ __

DATE: __ __ / __ __ / __ __ __ __

Oxygen and Flow Reduction Phase:

1. Step_____ Fi0_2 __.__ __ Liter flow __.__

	Minutes									
	1	2	3	4	5	6	7	8	9	10
Time	:	:	:	:	:	:	:	:	:	:
Saturation										
QS (Y/N)										

2. Step_____ Fi0_2 __.__ __ Liter flow __.__

	Minutes									
	1	2	3	4	5	6	7	8	9	10
Time	:	:	:	:	:	:	:	:	:	:
Saturation										
QS (Y/N)										

3. Step_____ Fi0_2 __.__ __ Liter flow __.__

	Minutes									
	1	2	3	4	5	6	7	8	9	10
Time	:	:	:	:	:	:	:	:	:	:
Saturation										
QS (Y/N)										

Continue wean if all saturations are ≥ 90%. Stop Wean and record as FAIL if:

Saturation <90% for 5 consecutive minutes

OR

Saturation <80% for 15 seconds

Record time to fail test

Record any adverse event (AE) with time of event:

Apnea>20 seconds

Bradycardia HR< 80 for> 10 seconds

Wean instructions:

If the infant is receiving supplemental oxygen by cannula:

A) Wean FiO_2 by 0.20 every 5 minutes until FiO_2 0.21
B) Record saturation every minute

Then wean the flow:

A) If the flow is ≥ 1.5 LPM, reduce flow by 1LPM. Monitor infant on reduced flow for 10 minutes.
B) Record saturation every minute
C) Once flow is< 1.5LPM reduce flow by 50%. Monitor infant on reduced flow for 10 minutes.
D) Record saturation every minute.
E) Gently remove nasal cannula from nares

After the nasal cannula has been removed, record saturations every 5 minutes until 60 minutes have elapsed

4. Step_____ Fi0$_2$ __.__ __ Liter flow __.__

	Minutes									
	1	2	3	4	5	6	7	8	9	10
Time	:	:	:	:	:	:	:	:	:	:
Saturation										
QS (Y/N)										

5. Step_____ Fi0$_2$ __.__ __ Liter flow __.__

	Minutes									
	1	2	3	4	5	6	7	8	9	10
Time	:	:	:	:	:	:	:	:	:	:
Saturation										
QS (Y/N)										

6. Step_____ Fi0$_2$ __.__ __ Liter flow __.__

	Minutes									
	1	2	3	4	5	6	7	8	9	10
Time	:	:	:	:	:	:	:	:	:	:
Saturation										
QS (Y/N)										

7. Step_____ Fi0$_2$ __.__ __ Liter flow __.__

	Minutes									
	1	2	3	4	5	6	7	8	9	10
Time	:	:	:	:	:	:	:	:	:	:
Saturation										
QS (Y/N)										

Continue wean if all saturations are ≥ 90%. Stop Wean and record as FAIL if:

Saturation <90% for 5 consecutive minutes

OR

Saturation <80% for 15 seconds

Record time to fail test

Record any adverse event (AE) with time of event:

Apnea>20 seconds

Bradycardia HR< 80 for> 10 seconds

Wean instructions:

If the infant is receiving supplemental oxygen by cannula:

C) Wean FiO$_2$ by 0.20 every 5 minutes until FiO$_2$ 0.21
D) Record saturation every minute

Then wean the flow:

F) If the flow is ≥ 1.5 LPM, reduce flow by 1LPM. Monitor infant on reduced flow for 10 minutes.
G) Record saturation every minute
H) Once flow is< 1.5LPM reduce flow by 50%. Monitor infant on reduced flow for 10 minutes.
I) Record saturation every minute.
J) Gently remove nasal cannula from nares

After the nasal cannula has been removed, record saturations every 5 minutes until 60 minutes have elapsed

8. Step_____ Fi0$_2$ __.__ __ Liter flow __.__

	Minutes									
	1	2	3	4	5	6	7	8	9	10
Time	:	:	:	:	:	:	:	:	:	:
Saturation										
QS (Y/N)										

Begin Room Air Observation

PASS: **All saturations ≥ 90% for 1 hour**

FAIL: **Saturation < 90% for 5 consecutive minutes**

OR

Saturation < 80% for 15 consecutive seconds

Record saturation every 5 minutes until 60 minutes have elapsed

Return infant to previous level of supplemental oxygen and flow

Results of Room Air Challenge

Pass **Fail (note reason for failure)**

Notify clinical care team of test results

	Minutes					
	5 min	10 min	15 min	20 min	25 min	30 min
1. Time	:	:	:	:	:	:
2. Saturation						
3. QS (Y/N)						

	Minutes					
	35 min	40 min	45 min	50 min	55 min	60 min

PID: __ __ __ __ __ __ __
DATE: __ __ / __ __ / __ __ __ __

1. Time	:	:	:	:	:	:
2. Saturation						
3. QS (Y/N)						

At conclusion of test:

Heart Rate: ___ ___ ___ (bpm)

RR: ___ ___ (breaths per minute. Count for a full minute.)

If FAIL, time to failure from initiation of oxygen/flow wean: ___ ___ ___ (in minutes)

If any AE occur, please complete AE Log. Record details below, as available.

AE (from time of initiation of wean through completion of Room Air Observation phase):

1. Apnea (cessation of breathing > 20 secs). Time ___ ___ : ___ ___
2. Bradycardia (HR < 80 bpm > 10 secs). Time ___ ___ : ___ ___

PID: __ __ __ __ __ __ __

DATE: __ __ / __ __ / __ __ __ __

NOTE: Please indicate date of assessment in date field

BASELINE DATA

1. Indicate baby's post-menstrual age at time of assessment — __ __ Weeks __ __ Day
2. What is the baby's body weight? — __ __ __ __ gms
3. Baseline Respiratory Rate (RR) — __ __ __ Breaths per minute
4. Baseline Heart Rate — __ __ __ Beats per minute
5. SpO2 — __ __ __. __ %

HYPOXIC CHALLENGE TEST [HCT]/ OXYGEN REDUCTION to 15%

6. Was the Hypoxic Challenge test performed? — ☐0 No ☐1 Yes
   6a. If 'No' please check primary reason infant missed test
   - ☐1 Baby was ineligible [Complete item 6b.]
   - ☐2 Baby was eligible but discharged or transferred
   - ☐3 Staff oversight/Staff or equipment not available to perform test
   - ☐4 Baby had acute respiratory illness
   - ☐98 Other, specify: ______________________________

   6b. If ineligible, please check primary reason for ineligibility
   - ☐1 Failed RAC
   - ☐2 Not weaned off clinical respiratory support
   - ☐3 Unable to maintain baseline saturation SpO_2 ≥90%
   - ☐4 On continuous respiratory support without RAC at 36 weeks PMA

7. Did Infant arouse (active sleep or awake) during the observation period? — ☐0 No ☐1 Yes
8. Please indicate the result of the Hypoxic Challenge test: — ☐1 Pass ☐2 Fail [Complete items 8a-b]

   8a. Record time to fail test: _______ minutes

   8b. Please indicate primary reason for test failure:
   - ☐2 Desaturation <85% for 60 consecutive seconds
   - ☐3 Desaturation <80% for 15 consecutive seconds
   - ☐4 Apnea
   - ☐5 Bradycardia
   - ☐98 Other, specify: ______________________________

9. Final Respiratory Rate — _______ Breaths/minute
10. Final Heart Rate — _______ Beats/minute
11. Final SpO_2 — __ __ __ %

Note: If any Adverse Events occurred during the assessment, record the details in the Adverse Event Log (AE).

PREMATURITY AND RESPIRATORY OUTCOMES PROGRAM

HYPOXIC CHALLENGE TEST_WORKSHEET

PID: __ __ __ __ __ __ __

DATE: __ __ / __ __ / __ __ __ __

Complete this form for all infants on whom you initiate a Hypoxic Challenge Test.

Instructions: Notify physician responsible for the infant's care (attending or fellow), and obtain MD order to perform Hypoxic Challenge Test.

- Challenge should be done with a pulse oximeter that has been getting a good signal for at least 5 minutes.
- Position infant supine, with any feedings or medications given 30 minutes prior to evaluation.
- Infant in Quiet Sleep (QS) at study initiation (Criteria: 1) Eyes closed and without movement, 2) Steady breathing, 3) Not vocalizing, and 4) No twitching of limbs (startles acceptable))
- Baseline data collection is done with the infant on room air
- Record oxygen saturation every 1 minute x 20 minutes
- Assess if infant is in Quiet Sleep (QS) every 5 minutes

Baseline Measurements

Heart Rate: ___ ___ ___ (bpm)
RR: ___ ___ (breaths per minute. Count for a full minute.)

Baseline Data	0 min	1 min	2 min	3 min	4 min	5 min
Time	:	:	:	:	:	:
Saturation						
QS (Y/N)						

	6 min	7 min	8 min	9 min	10 min	11 min	12 min	13 min	14 min	15 min
Time	:	:	:	:	:	:	:	:	:	:
Saturation										
QS (Y/N)										

Continue to the oxygen reduction phase if after 15 minutes all saturations recorded are ≥ 90%. If oxygen saturations are not maintained ≥ 90%, record infant as **Ineligible** for the Hypoxic Challenge and indicate reason for failure: **"Unable to maintain baseline SpO_2 ≥ 90%"**

PID: __ __ __ __ __ __ __

DATE: __ __ / __ __ / __ __ __ __

Oxygen Reduction Phase

- Place infant in high flow oxygen hood with oxygen sensor reading 15±1%
- Ensure oxygen analyzer reading has stabilized prior to initiation of data collection
- Record FiO_2 , Time, Saturation every minute for maximum of 20 minutes
- If infant has apnea during test, provide stimulation as per routine care.
- For persistent apnea or bradycardia turn off nitrogen, remove hood, may treat with supplemental oxygen if needed.
- At conclusion of test, turn off nitrogen gas and remove oxyhood.
- Record Heart Rate and Respiratory Rate
- Observe to assure saturation level return to baseline or > 85% on room air
- Supplemental oxygen may be used for persistent desaturation, until infant recovers to saturation > 85%

	Minutes									
	1	2	3	4	5	6	7	8	9	10
FiO_2										
Time	:	:	:	:	:	:	:	:	:	:
Saturation										
QS (Y/N)										

	Minutes									
	11	12	13	14	15	16	17	18	19	20
FiO_2										
Time	:	:	:	:	:	:	:	:	:	:
Saturation										
QS (Y/N)										

Record as PASS if saturations are ≥ 85 % for 20 minutes

Stop challenge and record as FAIL if:

Saturation <85% for 60 consecutive seconds

OR

Saturation < 80% for 15 consecutive seconds

OR

Any need to prematurely terminate the test (bradycardia or persistent apnea)

Record time to fail test (in minutes)

Record any adverse event (AE) with time of event:

Apnea >20 seconds

Bradycardia HR <80 for >10 seconds

Additional AE/SAE following termination of exposure to FiO2 0.15, below

PID: __ __ __ __ __ __ __

DATE: __ __ / __ __ / __ __ __ __

Results of Hypoxic Challenge Test

Pass or **Fail (note reason for failure)**

Desaturation
Persistent apnea > 20 secs
Bradycardia < 80 bpm x 10 secs

Notify clinical care team of the test results

At conclusion of test:

Heart Rate: ___ ___ ___ (bpm)

RR: ___ ___ (breaths per minute. Count for a full minute.)

If FAIL, time to failure from initiation of exposure to FiO_2 0.15: ___ ___ (in minutes)

If any AE or SAE occur, please complete AE/SAE Log. Record details below, as available.

AE (from time of initiation of FiO_2 0.15):

1. Apnea (cessation of breathing > 20 secs). Time ___ ___ : ___ ___
2. Bradycardia (HR < 80 bpm > 10 secs). Time ___ ___ : ___ ___

AE (following completion of test):

1. Supplemental oxygen of $FiO_2 \geq 0.10$ above baseline persisting for > 1 hour after test is concluded or terminated.
2. Tachycardia (> 200 bpm) persisting for > 1 hour after test is concluded or terminated.

SAE (to be reported if possibly or probably related to study procedures):

1. Any need for CPR within 12 hours after test is concluded or terminated (*i.e.,* chest compressions or cardiac medications given for resuscitation). Date ___ ___ / ____ ___ / ___ ___ Time ___ ___ : ___ ___
2. The addition of mechanical ventilation within 12 hours after the test is concluded or terminated. Date ___ ___ / ____ ___ / ___ ___ Time ___ ___ : ___ ___
3. Supplemental oxygen of $FiO_2 \geq 0.10$ above baseline persisting for ≥ 12 hours after test is concluded or terminated. Date ___ ___ / ____ ___ / ___ ___ Time ___ ___ : ___ ___
4. Any additional event deemed to be serious by the site Principal Investigator and possibly or probably related to study procedure. Date ___ ___ / ____ ___ / ___ ___ Time ___ ___ : ___ ___
