# Supplementary material for: Prematurity and respiratory outcomes program (PROP): study protocol of a prospective multicenter study of respiratory outcomes of preterm infants in the United States
Source: BMC Pediatr. 2015 Apr 10;15:37. doi: 10.1186/s12887-015-0346-3 (PMC4407843; doi:10.1186/s12887-015-0346-3)
Supplement: Additional file 8: Table S1. — Infant pulmonary function test measurements. [file 12887_2015_346_MOESM8_ESM.docx]

**Supplemental Table 1: Infant Pulmonary Function Test Measurements**

| **Measurement** | **Method** |
| --- | --- |
| Tidal breathing analysis (which includes Tpef/Te) | Mask Pneumotachometer |
| Crs and Rrs (compliance and resistance) | Single breath occlusion |
| FRC measurement | Whole body plethysmography |
| Forced expiratory flows (FVC, FEV0.5, FEF25-75, FEF75) | Raised volume rapid thoraco-abdominal compression technique |
| Fractional lung volumes (ERV, TLC, RV/TLC, FRC/TLC) | Raised volume rapid thoraco-abdominal compression technique and plethysmography |
| Bronchodilator response (Forced expiratory flows from raised volumes) | Raised volume rapid thoraco-abdominal compression technique |
