# Supplementary material for: Prematurity and respiratory outcomes program (PROP): study protocol of a prospective multicenter study of respiratory outcomes of preterm infants in the United States
Source: BMC Pediatr. 2015 Apr 10;15:37. doi: 10.1186/s12887-015-0346-3 (PMC4407843; doi:10.1186/s12887-015-0346-3)
Supplement: Additional file 9: — PROP biospecimen access policy. [file 12887_2015_346_MOESM9_ESM.pdf]

PROP BIOSPECIMEN ACCESS POLICY
ADAPTED 12/12/13 and Revised 4/9/14

<u>Outline</u>

1. Overview/history
2. Biospecimens in repositories
3. Clinical data available
4. Functions of biorepositories
5. Maintenance of biorepositories after PROP
6. Application process for biospecimens
7. Review of applications
8. Costs of dispersing biospecimens
9. Acknowledgement of PROP for studies using biospecimens
10. Appendix

This policy statement describes the biospecimen repositories associated with the Prematurity and Respiratory Outcome (PROP) program. The repositories contain a collection of tracheal aspirate, urine and DNA samples from premature infants enrolled in the PROP observational multicenter study; each sample is linked to a substantive database of clinical information for the infants from birth through the first year after discharge. Samples are available to PROP and other investigators for approved ancillary studies of biomarkers related to the clinical course and pulmonary outcome. The following describes the policy recommended by the PROP Biorepository Subcommittee and approved by the PROP Biospecimen and Steering Committees for maintaining the repositories and dispensing samples to investigators before and after completion of PROP.

1. <u>Overview/history</u>

The tracheal aspirate (TA), urine and DNA repositories were established in 2011 in conjunction with initiation of the PROP multicenter observational study to collect clinical data and biomarkers on a cohort of 750 surviving infants <29 wk gestational age. Eligible, parentally-consented infants ≤7 days old were enrolled at 6 clinical centers (University of California San Francisco, Washington University, Cincinnati Childrens Hospital, Vanderbilt University, Universities of Rochester and Buffalo, and Duke/Indiana Universities) comprised of a total of 13 clinical sites. A total of 835 infants were enrolled in the study with an estimated 750 surviving to 36 weeks PMA (or hospital discharge). TA and urine samples were collected as soon as possible after enrollment and at 7±1, 14±1 and 28±2 days postnatal age using standardized collection kits and collection protocols. The TA repository is currently located in the P. Ballard laboratory at UCSF and urine samples are stored at in the Aschner/Fike laboratory at Vanderbilt. Buccal swab (infants) and saliva (parents) samples were collected using Oragen kits and the DNA was isolated and stored at the DNA Resources Core at the Center for Human Genetics Research at Vanderbilt University (DNA Core). Biospecimens in the repositories and linked clinical database (University of Pennsylvania) are available to support additional biomarker studies by PROP investigators and will eventually be available to non-PROP investigators. The repositories are maintained through PROP funding, which will terminate in April 2015. Further details regarding the collection and handling of specimens plus clinical information obtained are contained in the Manual of Operations (Appendix).

2. Biospecimens available

- *Tracheal aspirate.* These samples were collected from intubated infants by intratracheal instillation of 0.5 ml of normal saline and aspiration of the fluid; this procedure was repeated and the collected fluid was combined with 1 ml of saline used to rinse the suction trap. The sample was centrifuged at 300xg to yield a cell pellet and supernatant TA that was divided into 4 aliquots of 0.25 ml plus a residual aliquot. Both supernatant and cell pellet samples were frozen, shipped to UCSF, catalogued and stored at -80C. Sites performing TA biomarker studies had the option to retain 1 or 2 aliquots of TA or the cell pellet. Currently there are 3-5 TA aliquots and 1 tube of TA cells/collection for most enrolled infants stored in the repository. The total number of samples will be known in 2014.
- *Urine.* These samples were collected from infants by placing cotton balls in the diaper for 2-8 h and then expressing the urine using a syringe. The sample was divided into 4 aliquots of 0.25 ml plus a residual aliquot. Samples were frozen, shipped to Vanderbilt, catalogued and stored at -80C. Sites performing biomarker studies on urine had the option to retain 1 or 2 aliquots of urine. Currently there are 3-5 urine aliquots/collection for most enrolled infants stored in the repository. The total number of samples will be known in 2014.
- *DNA.* Mucosal cells were collected using infant and adult Oragen saliva collection kits that were then shipped to the DNA Core at Vanderbilt University (Cara Sutcliffe manager; cara.b.sutcliffe@vanderbilt.edu). DNA was isolated, quantitated (Nanodrop and picoGreen) and is stored at the DNA Core. A portion of DNA from 192 infants has been used for a whole exome sequencing project. The total number of samples will be known in 2014.

3. Clinical data available

Each sample is linked by an Accession Number to an extensive clinical database in Oracle at the DCC at the University of Pennsylvania. Investigators requesting biospecimens can contact the DCC (Melissa Fernando [fernand@mail.med.upenn.edu]) to identify a cohort of infants with the desired demographics and clinical course/outcome for their proposed studies.

4. Functions of the biorepositories

- *TA and urine*. These 2 repositories have received shipments of collected biospecimens during the enrollment period. The aliquots of samples are logged in using a bar coded system, organized in freezer boxes and stored in remote-alarmed -80C freezers. For approved biospecimen requests, repository personnel will locate aliquots of the target group of samples, prepare a shipping list, and ship the samples on dry ice for next-day delivery.
- *DNA.* The DNA Core stores DNA samples using an automated system. For approved DNA requests, Core personnel will locate samples of the target group, take the desired aliquot of samples, prepare a shipping list, and ship the samples for next day delivery to the investigator at investigator expense.

5. Maintenance of biorepositories after PROP

- *TA and urine*. Sufficient PROP funding is anticipated to maintain biorepository services through year 05 of the grant (March 31 2015). Beyond that time, it is proposed that the current laboratories maintain the repositories as long as feasible on a fee-for-service basis and funding from other sources as needed/available. When the Ballard and/or Aschner/Fike laboratories are no longer able to maintain the repositories (due to lab closure, PI retirement or relocation, etc.), other ex-PROP investigators will be approached to assume the responsibility if sufficient samples and interest remain. Alternatively, at that time, the samples can be transferred to the NIH BioLINK repository. This plan assumes that the clinical data at the DCC remain available to investigators upon request and approval.
- *DNA*. The DNA Core at Vanderbilt has agreed to maintain PROP DNA samples for the foreseeable future and to accommodate approved requests for DNA aliquots.

6. Application process for biospecimens
    - Applicants will submit an application proposal for biorepository samples. The application describes the project, provides supporting data, includes a general description of type, number and postnatal age of samples requested along with a power analysis, and states the funding source for the study (see Appendix for proposal instructions). If the request is approved, the investigator contacts the DCC to generate a list of patients and collection ages with available samples, and the biorepository personnel selects and ships the aliquots.
    - The deadlines for submitting applications for review are January 2, April 1, July 1, and October 1. Applicants are encouraged to contact the Biospecimen Committee before submitting the application to discuss the project and proposed sample request.

7. Review of applications
    - Applications for biorepository samples are reviewed by the PROP Biospecimen Committee within 4 weeks of the submission deadline. Criteria for evaluating and scoring applications are described in the Appendix. The recommendation of the Committee is then presented to the next meeting of the Steering Committee, until the PROP study has closed formally, for discussion and approval.
    - Priorities for access to samples by category of investigator are listed below. In general, samples will not be available to non-PROP investigators until 1 year after closure of the grant (including any No Cost Extensions).
        a) PROP investigators for initiating new PROP multi-center studies (eg., PROP2)
        b) PROP investigators for confirming single-site findings
        c) Investigators affiliated with PROP personnel
        d) Non-PROP investigators
    - Approved applications are forwarded to the appropriate repository and samples are retrieved and shipped to the investigator within 4 weeks.
    - Review of applications after completion of PROP funding will be done by a committee consisting of the directors of the repositories at that time, available and interested members of the current Biospecimen Committee, and a representative of the Lung Division of NHLBI.

8. Costs of dispersing biospecimens

- For PROP investigators, the costs for collecting and shipping approved urine and TA samples during years 4 and 5 of PROP will be covered by the Multicenter budgets for the respective repositories. In the event that there is an approved application by a non-PROP investigator during these years, a fee will be charged for the service of supplying samples. In year 6 of PROP and thereafter, requests for urine and TA samples will incur a cost as determined by each repository.
- For DNA samples, first time requests for an aliquot of DNA from that sample will be without charge; for second and further requests for aliquots of a DNA sample (even if the request is from a different or new investigator), the DNA Core will charge a modest per sample cost.

9. Acknowledgement of PROP for studies using biospecimens

Publications of studies using any PROP biospecimens should acknowledge the appropriate PROP Repository, PROP DCC and funding from the Prematurity and Respiratory Outcome (PROP) program (HL 101798). Manuscripts must be reviewed by the PROP Publication Committee and approved by the Steering Committee according to the current PROP Publication Policy. If a manuscript is prepared after closure of the PROP grant, it should be submitted to each of the PROP PIs for review and the PIs should be listed as authors if desired.

Appendix:

1. Applications for Biospecimens: Instructions

The cover page lists the applicants with their affiliations, PROP status and contact information along with the title of the proposed study. In ~3 additional pages, describe 1) the hypothesis and rationale, 2) background information and any preliminary data, 3) type, number and volume (urine and TA) or amount (DNA) of samples requested, including a power analysis to justify the number of samples requested, 4) methodology, 5) type of clinical data needed, and 6) funding source for the studies. After approval of the application, Repository and DCC personnel will work with the applicants to develop a list of samples and the specific, linked clinical data to be provided.

2. Biorepository Access Review Procedure and Criteria

Applications for use of the PROP Biorepository will be accepted and evaluated based upon merit. Those applications deemed to be of sufficient merit will be approved, and access to the repository will be granted, so long as sufficient samples and funding sources are available. Biospecimens are anticipated to be used for the following purposes, listed in general order of enthusiasm; 1) initiation of new PROP multicenter studies, 2) confirmation of existing single center studies by PROP investigators, 3) initiation of new single center studies by existing PROP investigator, 4) initiation of new studies by non-PROP investigators. Applications should clearly identify the nature of the biospecimen use request, and provide rationale for how the request meets this criterion. Applications from investigators not currently part of the PROP will not be considered until 1 year following completion of the multi-center studies.

The major criterion for review will be scientific merit for the proposed study. Merit will be evaluated based upon the state of the field, rationale provided by the applicant, supporting preliminary data (if available) and the presence of sufficient sample(s) available to adequately complete the proposed studies. A formal statistical assessment of the number and type of samples necessary to achieve the goals of the study is required, particularly a sample size calculation in the event of a "negative" result. In addition, applicants must clearly demonstrate the availability of existing funds to complete data collection, assay analysis and statistical analysis for the requested samples. Finally, applications will be evaluated for alignment with the original goals of the PROP; i.e. to identify biomarkers and mechanisms for chronic respiratory morbidity following premature birth, although this is not a mandatory requirement.

Applications will be accepted for review on January 2, April 1, July 1, and October 1. Under extraordinary circumstances, applications may be accepted for review outside these deadlines, if appropriate justification is provided. Applications will be appraised individually, by all active members of the PROP Biospecimen Committee, as defined by the Steering Committee. Formal Biospecimen Committee reviews will either be part of existing, scheduled Biospecimen Committee teleconferences, or will be scheduled in a timely manner (2-4 weeks after each submission date). Each application will be presented, in summary form, to the committee by an assigned Biospecimen Committee member. Following a consideration of comments by all members, applications will be scored to be either; 1) of the highest merit, 2) of potential merit, or 3) not appropriate. Those applications scored at the highest merit will be recommended to the PROP Steering Committee for approval at the next scheduled monthly meeting. Applicants for proposals scored "of potential merit" or proposals judged to need additional information will be provided comments and/or questions, and will be allowed to resubmit a revised proposal for a later cycle.

3. Current members of the Biospecimen Committee

Tom Mariani, Rochester, Tom_Mariani@urmc.rochester.edu (chair)
Judy Aschner, Vanderbilt, JASCHNER@montefiore.org (co-chair and urine repository director)
Philip L. Ballard, UCSF, ballardp@peds.ucsf.edu (tracheal aspirate repository director)
Aaron Hamvas, Washington University, hamvas@kids.wustl.edu
Rui Feng, Penn(DCC), ruifeng@mail.med.upenn.edu
Mark O'Hunt, Vanderbilt, mark.o.hunt@Vanderbilt.Edu (urine repository personnel)
Cheryl Chapin, UCSF, Cheryl.chapin@ucsf.edu (tracheal aspirate repository personnel)
Hart Horneman, UCSF, HornemanH@PEDS.UCSF.EDU (tracheal aspirate repository personnel)
Claire Chougnet, Cincinnati, claire.chougnet@cchmc.org
Cara Sutcliffe, cara.b.sutcliffe@Vanderbilt.Edu (DNA repository director)
Gloria Pryhuber, Rochester, gloria_pryhuber@urmc.rochester.edu
Carol Blaisdell, NIH/NHLBI, blaisdellcj@nhlbi.nih.gov
Lynn Taussig, lynn.taussig@du.edu
Barry Schmetter, NIH/NHLBI, Barry.Schmetter@nih.hhs.gov
Barbara Schmidt, Penn (DCC), barbara.schmidt@uphs.upenn.edu
Denise Cifelli', Penn (DCC), cifelli@mail.med.upenn.edu
Tom Ferkol, Washington University, Ferkol_T@kids.wustl.edu
Jonas Ellenberg, Penn (DCC), jellenbe@mail.med.upenn.edu

Kurt Schibler, Cincinnati, kurt.schibler@cchmc.org
Marshall Summar, Vanderbilt, Summar@CHILDRENSNATIONAL.ORG
Vasanth Kumar, Buffalo, vkumar@upa.chob.edu
Alan Jobe, Cincinnati, ALAN.JOBE@cchmc.org
Rita Ryan, Buffalo, ryanr@MUSC.EDU
